# Multiomic profiling links L1 retrotransposition to genomic instability and ecDNA in bladder cancer

**DOI:** 10.1038/s41467-026-75399-6

**Published:** 2026-07-16

**Authors:** Sophia J. Pribus, Ivana Osredek, Jan Otoničar, Milena Simovic-Lorenz, Chiara Giovenino, Anne Rademacher, Sina Jasmin Wille, Cecilia Berzain Battioni, Michael Scherer, Sergio Manzano-Sanchez, Andreas Kienzle, Urja Parekh, Laura Villacorta, Vladimir Benes, Pooja Sant, Jan-Philipp Mallm, Karsten Brand, Karsten Rippe, Angelika B. Riemer, Holger Sültmann, Christoph Plass, Mladen Stankovic, Jan O. Korbel, Tobias Rausch, Aurélie Ernst

**Affiliations:** 1 European Molecular Biology Laboratory, Genome Biology Unit, Heidelberg, Germany; 2 Stanford Cancer Institute, School of Medicine, Stanford University, Stanford, CA USA; 3 Faculty of Biosciences, Heidelberg University, Heidelberg, Germany; 4 Division of Genome Instability and German Cancer Consortium (DKTK), German Cancer Research Center (DKFZ), Heidelberg, Germany; 5 Division of Chromatin Networks, German Cancer Research Center (DKFZ) Heidelberg, Heidelberg, Germany and Center for Quantitative Analysis of Molecular and Cellular Biosystems (BioQuant), Heidelberg University, Heidelberg, Germany; 6 Division of Cancer Epigenomics, German Cancer Research Center (DKFZ), Heidelberg, Germany; 7 Division of Immunotherapy and Immunoprevention, German Cancer Research Center (DKFZ), Heidelberg, Germany; 8 Medical University of Vienna, Vienna, Austria; 9 European Molecular Biology Laboratory, Genomics Core Facility, Heidelberg, Germany; 10 Single-cell Open Lab, German Cancer Research Center (DKFZ) and Bioquant, Heidelberg, Germany; 11 MVZ of Pathology, Heidelberg, Germany; 12 German Center for Infection Research (DZIF), Partner Site Heidelberg, Molecular Vaccine Design, Heidelberg, Germany; 13 Division of Cancer Genome Research, German Cancer Consortium (DKTK) and German Cancer Research Center (DKFZ), Heidelberg, Germany; 14 Department of Urology, Salem Hospital, Academic Hospital, University of Heidelberg, Heidelberg, Germany; 15 Bridging Division Mechanisms of Genomic Variation and Data Science, German Cancer Research Center (DKFZ), Heidelberg, Germany

**Keywords:** Cancer genomics, Bladder cancer

## Abstract

Bladder cancer is frequent and highly recurrent. Despite recent advances, knowledge gaps remain in molecular mechanisms underlying disease progression. In this study, we apply integrated multi-omic analyses to a cohort of 48 bladder cancer patients to comprehensively profile genetic, epigenetic, transcriptomic, and spatial features. Combining cell-free DNA sequencing, long-read tumor DNA-sequencing, RNA-sequencing, and spatial transcriptomics, we explore molecular alterations driving bladder cancer. We find frequent somatic LINE-1 (L1) insertions, and show that these L1 insertions are active and occur early in bladder cancer development. We link somatic L1 insertion with downstream genomic rearrangements and chromosomal instability, including increased structural variant and extrachromosomal DNA (ecDNA) counts in patients with high L1 counts. We identify variable ecDNA enrichment across tissue architecture, with highest enrichment overlapping differential expression of *APOBEC3B* and immune response pathways. In summary, our results support a model whereby L1 retrotransposition triggers downstream genomic instability and viral mimicry response.

## Abstract (web summary)

Bladder cancer shows high recurrence, and its molecular drivers remain incompletely understood. Here, authors use integrated multi-omic and spatial profiling of patient tumors to show that early LINE-1 retrotransposition is associated with genomic instability, extrachromosomal DNA, and activation of immune-related pathways.

## Introduction

Bladder cancer is one of the most frequent malignancies worldwide, with an estimated 573,000 new cases and 213,000 deaths occurring globally each year^1^. It arises from the urothelial lining of the bladder and is classified into two major types: non-muscle invasive bladder cancer (NMIBC) and muscle invasive bladder cancer (MIBC)^2^. NMIBC accounts for ~70–75% of cases at diagnosis and when diagnosed early shows high rates of survival^3,4^; MIBC is more aggressive and has a worse prognosis^2,4^. Despite improvements in detection and treatment, bladder cancer remains challenging to treat due to frequent transitions from NMIBC to MIBC^5^, propensity for recurrence and metastasis^2^, and resistance to current therapies^2,6–10^. These challenges are especially pronounced in the surveillance of recurrent disease^2^, where clinical ambiguity can lead to overtreatment or delayed intervention. Overall, there is a lack of understanding of the clinical and genomic features that influence disease aggressiveness; addressing this gap would support the development of improved diagnostic and prognostic tools, and facilitate the development of more effective treatment strategies. Thus, a more comprehensive molecular characterization of bladder cancer development may pave the way to significantly improve patient outcomes. Earlier detection and improved risk stratification have the potential to significantly decrease bladder cancer-related death^11–13^. While cystoscopy and urine cytology are widely used in clinical practice, these methods are invasive, costly, and often fail to detect tumors at early stages^14^. Moreover, they lack specificity and the sensitivity to distinguish between benign and malignant lesions, or to predict which patients are at risk of disease progression^2^. A deeper understanding of the drivers of disease initiation and development through integrated clinical and genomic profiling has the potential to reduce invasiveness and improve accuracy of early detection and risk stratification for patients. In addition, the incomplete understanding of the disease hinders the development of targeted treatment approaches. While a number of molecular studies^15–21^ of bladder cancer have identified key driver genetic alterations and pathways, none to our knowledge have combined comprehensive genomic, epigenomic, transcriptomic, and spatial data to reveal novel, bladder cancer-specific disease mechanisms. Such a study could lay the basis to understand recurrence mechanisms, identify innovative therapeutic targets, and explore candidate biomarkers for personalized interventions.

The potential of advanced genomic technologies, including long-read sequencing and spatial transcriptomics, to improve our mechanistic understanding of bladder cancer progression has, to our knowledge, not been fully exploited. One such mechanism that can be explored through the use of such next-generation sequencing technologies is the somatic retrotransposition of LINE-1 (L1). Current estimates suggest that L1 sequences make up roughly one-fifth of the human genome, and while the majority of these are inactivated via epigenetic silencing, the small fraction of active L1s are hypothesized to have an impact on cancer progression^22^. This has been suggested in a bladder cancer context, where L1 elements become active via hypomethylation and contribute to genomic instability^23^. Increasing evidence indicates that the DNA damage associated with L1 insertion may influence downstream mechanisms of genomic alterations and immune response via viral mimicry^22^. However, comprehensive studies of these mechanisms are lacking in clinical samples, including in bladder cancer. Linking L1 insertions with aggressive disease characteristics such as genomic rearrangements, amplifications, ecDNA and upregulated immune response would solidify the potential of L1 as a bladder cancer biomarker.

In cell lines, L1 insertions have been shown to upregulate DNA damage mechanisms by causing double-stranded DNA breaks and have been hypothesized to contribute to chromosomal instability in cancer^22,24,25^. Importantly, pan-cancer analyses have shown that somatic L1 retrotransposition induces breakage-fusion-bridge cycles and chromothripsis^22^, known precursors to extrachromosomal DNA (ecDNA)^26^; indeed, in vitro studies are beginning to identify the presence of L1 insertions on ecDNA structures^27–29^. However, a clear link between L1 insertions and ecDNA presence has not been established in a clinical cancer context. This is especially interesting, given that L1 insertions have been shown to trigger APOBEC3B-mediated mutagenesis^20^, and APOBEC3B activity has been shown to facilitate ecDNA evolution^30^. Demonstrating a link between L1 insertion count and ecDNA presence could suggest a potential “double-hit” mechanism via which L1 insertions concurrently trigger APOBEC3B and chromosomal instability leading to ecDNA formation, and maintained APOBEC3B activity further mutates ecDNA, facilitating downstream tumor aggressiveness.

In cell lines and in tumors outside the bladder cancer context, L1 expression has been linked to immune response via “viral mimicry”, a process by which endogenous virus response mechanisms are activated by non-viral genetic material in the cytoplasm. It has been proposed that L1 insertions are a source for double-stranded DNA (dsDNA), double-stranded RNA (dsRNA), and DNA:RNA hybrids, all of which, when detected in the cytoplasm, may elicit the innate type I interferon (IFN-I) response^22,31,32^. To substantiate these claims, it is important to first establish the upregulation of such response pathways, including the cGAS-STING pathway (which responds to dsDNA and DNA:RNA hybrids) and the RIG-I and MDA5 sensors (which respond to dsRNA). This can be addressed through both RNA expression analysis and spatial transcriptomics for additional spatial resolution of response pathways.

In this work, by integrating multi-omics and spatial approaches, we aimed to gain a comprehensive view of the genetic, epigenetic, transcriptomic, and spatial characteristics of bladder tumors, alongside blood-based analysis of cfDNA. We included both NMIBC and MIBC patients across tumor grades and stages, including benign lesions, to capture the entire spectrum of bladder cancer. The combination of low-pass whole-genome sequencing of cfDNA, Oxford Nanopore long-read sequencing for tumor genomes, matched germline DNA-sequencing, RNA-sequencing, and spatial transcriptome analysis provides a holistic view of the (epi)genetic alterations and gene expression changes driving bladder cancer. In particular, we identify for the first time in a clinical bladder cancer cohort a comprehensive connection between L1 insertions, chromosomal rearrangements including ecDNA, and potential downstream viral mimicry effects. By generating comprehensive molecular insights into bladder cancer biology, this work suggests the potential of L1 elements as a biomarker in bladder cancer and elucidates possible mechanisms of disease progression via L1 insertion-mediated genomic instability.

## Results

### Patient cohort

**Fig. 1:**
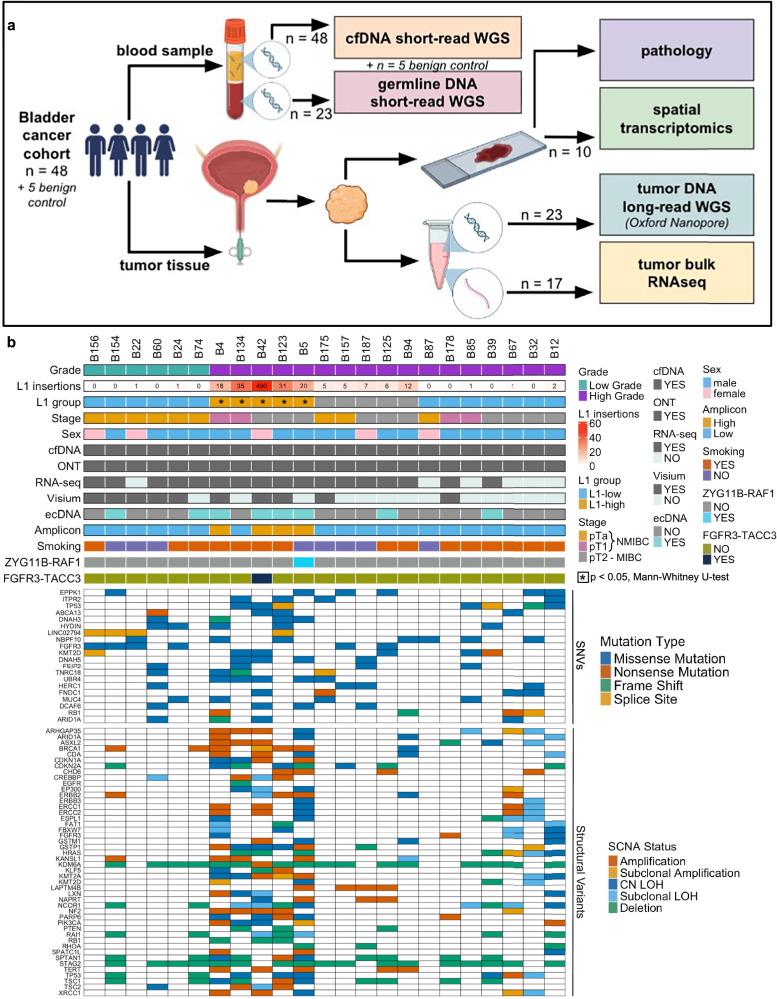
Multi-omic analysis reveals multiple forms of genome instability in bladder cancer. **a** Schematic of patient cohort and data types (created with bioRender). 48 bladder cancer patients underwent blood draws and transurethral resection of bladder tumor. Blood samples were used for cfDNA analysis with short-read WGS. Tumor samples were used for spatial transcriptomics, genomic (long-read WGS) and RNA-Seq analysis. **b** Oncoplot summarizing patient-specific tumor characteristics (stage and grade, ecDNA status, somatic L1 insertions), sex, available data types, driver gene-fusions and other alterations in key bladder cancer driver genes detected by long-read sequencing. CN = copy number, CN LOH = copy-neutral loss of heterozygosity, NMIBC = non-muscle invasive bladder cancer, MIBC = muscle-invasive bladder cancer. L1-low <5: *n* = 5, L1-high > 15: *n* = 5 (L1 count) demonstrates the correlation between the cancer grade and L1 count (Supplementary fig. 8e). Source data are provided as a Source Data file.

Understanding the molecular landscape of MIBC and NMIBC is essential to improve risk stratification and to develop early detection approaches as well as targeted treatment strategies. We present a comprehensive multi-omic characterization of tumors and blood from 48 patients with MIBC, NMIBC as well as 5 benign lesions (Fig. 1a). The tumors represent the whole disease spectrum of bladder cancer, including low-grade as well as high-grade tumors of multiple stages. A full description of the cohort is displayed in Supplementary Data 1. We analyzed blood cell-free DNA (cfDNA) using low-pass whole-genome sequencing (*n* = 48, mean coverage: 4.5x). We performed long-read whole-genome sequencing using Oxford Nanopore technology (ONT) (*n* = 23, mean coverage: 32x, N50 read length: 15.7Kbp) and short-read sequencing of matched germline DNA (*n* = 23, mean coverage: 17x). Furthermore, we conducted spatial transcriptomics analyses using the Visium platform for 10 tumors representative from the different stages and grades, and total RNA-sequencing (RNA-seq) for 17 tumors (see Supplementary Data 1 for details).

We first explored gene-level alterations that could contribute to cancer development in our cohort. Analysis of somatic copy-number alterations (SCNAs) revealed recurrent somatic alterations affecting established bladder cancer driver genes (Fig. 1b). The most frequently altered genes included *KDM6A* (altered in 73.9% of the tumors, with 94.1% showing deletions), *STAG2* (73.9%, 100% deletions), *NCOR1* (43.5% with deletions and subclonal loss of heterozygosity (LOH), each contributing 40%), *SPTAN1* (43.5%, 70% of deletions), *TP53* (43.5%, 40% LOH), *TSC1* (43.5%, 70% of deletions) and *RAI1* (39.1%, 55.6% of deletions). We also analysed single nucleotide variants (SNVs) (Fig. 1b). The most frequently altered gene was *NBPF10*, mutated in 30.4% of the tumors through missense mutations (100%). Other commonly affected genes included *EPPK1* (26.1%, 100% missense), *FGFR3* (26.1.1%, 100% missense), and *KMT2D* (26.1%, 100% splice site mutations). A full list of affected genes and the respective alterations are available in Supplementary Data 2–3.

We further identified 65 high-confidence gene fusions, supported at both the DNA (ONT reads) and RNA (RNA-seq) levels (Supplementary Data 4). The *FGFR3-TACC3* fusion (Supplementary Fig. 1a), previously reported in bladder cancer^33,34^, leads to constitutive activation of FGFR3 signaling, promoting cell proliferation and survival. The *ZYG11B-RAF1* fusion (Supplementary Fig. 1b), not previously identified in bladder cancer, potentially drives cancer development, as suggested by gene fusions involving *RAF1* identified in other cancers and by activations of *RAF1* through fusion-independent mechanisms such as amplification in approximately 12–20% of MIBC. In the germline, we also identified 12 high-impact pathogenic variants using Variant Effect Predictor (VEP)^35^, 33 variants of moderate impact and two modifier variants, respectively (see Supplementary text for details).

### Multi-omic landscape of bladder cancer: long-read, spatial, and cell-free DNA analysis enable detailed dissection of genomic instability and retrotransposition events within the tissue context

**Fig. 2:**
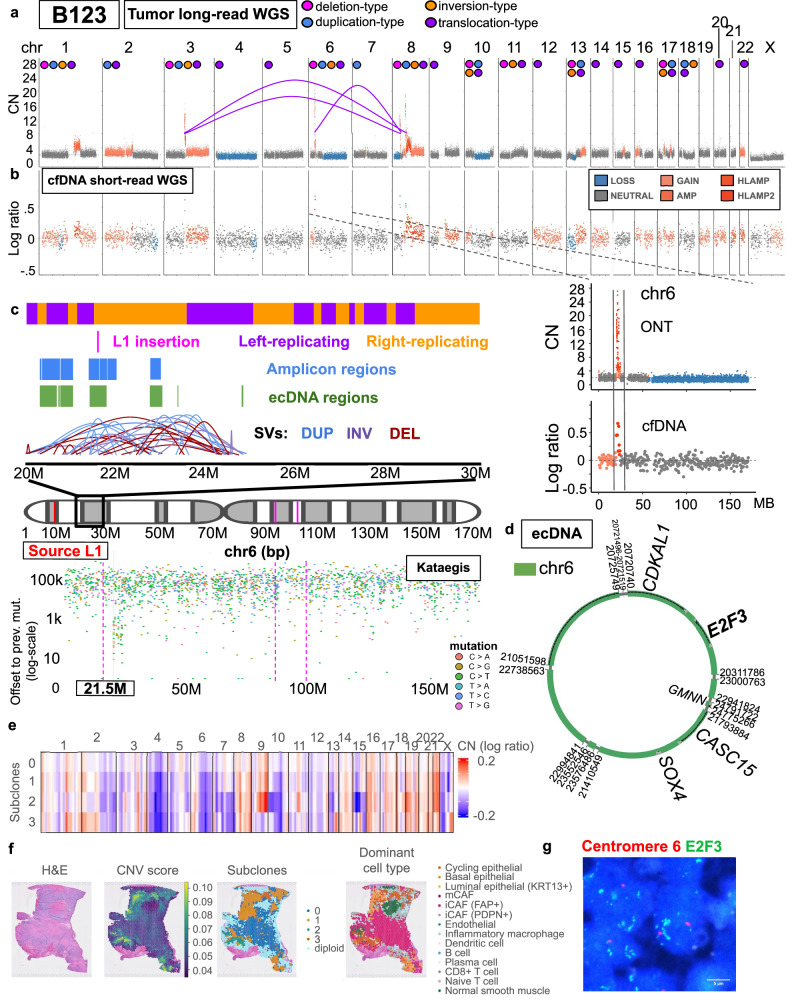
Long-read, spatial, and cell-free DNA analysis allows dissecting the landscape of tumor cell clones, extrachromosomal circular DNA and retrotransposition within the tissue architecture. **a** Long-read WGS of B123 tumor tissue reveals broad copy-number variation and sporadic high copy-number amplifications. Chromosomes are labeled with a subset of the detected structural variation events; a subset of interchromosomal translocation events where both ends border amplicon events (within 100kbp) are displayed as purple arcs. LOSS: CN < 1.5, NEUTRAL: 1.5 < = CN < 2.5, GAIN: 2.5 < = CN < 3.5, AMP: 3.5 < = CN < 4.5, HLAMP: 4.5 < = CN < 5.5, HLAMP2: CN > = 5.5 **b** cfDNA short-read WGS shows matched copy-number variations between B123 blood and tumor samples and in particular, specific amplification events on chromosomes 3, 6, and 8. LOSS: CN < 2, NEUTRAL: CN = 2, GAIN: CN = 3, AMP: CN = 4, HLAMP: CN = 5, HLAMP2: CN > = 6. **c** Schematic of focal genomic events on 6p21 in sample B123, including L1 insertions, amplicon regions, ecDNA regions, structural variations, and kataegis events (chromosome schematic from BioRender). **d** Schematic of ecDNA detected in B123 made with CycleViz, including amplifications of *E2F3*, *CDKAL1*, *GMNN*, *CASC15*, and *SOX4*. **e** Pseudobulk copy-number profiles of genetic clones inferred from Visium data (sample B123). **f** From left to right: Hematoxylin and eosin stain, CNV score from Visium, genetic subclones shown in (**e**) and the dominant cell type per spot (sample B123). **g** FISH validation of ecDNA carrying the *E2F3* oncogene (B123).

We next extended our analysis beyond the gene level by applying an integrative multi-omic profiling approach to uncover mechanisms driving cancer evolution and crosstalk with the microenvironment. For all samples in the cohort, we dissected the interplay between genome and epigenome from ONT sequencing data as well as cfDNA analysis. We further explored these relationships spatially, mapping the genetic subclone composition and spatial patterns within the tissue architecture. To demonstrate the value of this multi-omic approach, we highlight our comprehensive analysis of tumor, cfDNA and germline DNA from patient B123 in Fig. 2.

In patient B123, ONT sequencing revealed multiple focal amplifications and 137 structural variants (SVs), including 47 inter-chromosomal events (Fig. 2a). Across the whole cohort, we identified at least one somatic SV in all ONT-sequenced tumors (Supplementary Fig. 2). For patient B123, copy-number alterations in cfDNA reflected those identified using ONT sequencing in the matched tumor tissue (Fig. 2b). However, when considering the whole cohort, only a minority of cfDNA samples showed detectable copy-number changes, with evidence for enrichment in high-grade tumors (4/25 high-grade tumors, 0/23 low grade, p = 0.06, Fisher’s exact test, one-sided, Supplementary Fig. 3a–c). There was also evidence for chromothripsis in 12/23 patients (Supplementary Fig. 2), including patient B123 (Fig. 2a). We further detected ecDNA in 7/23 patients, harboring oncogenes such as *CCND1*, *MDM2* and *E2F3* (Supplementary Data 1 and 5), including B123 (Fig. 2c, d). Beyond ecDNA, we also detected indications of oncogene activation through enhancer hijacking by computational prediction with *pyjacker*^36^ (Supplementary Fig. 4a–d), identified as DNA rearrangements juxtaposing an enhancer nearby an oncogene coupled with a strong overexpression. Enhancer hijacking was frequent among ecDNA-associated overexpressed genes (Fisher test p-value < 10e-16), consistent with prior work^37^ showing that ecDNA co-occur with enhancer hijacking to express oncogenes (Supplementary Fig. 4e). Alignment to the CHM13 (T2T) assembly^38^ and calculation of a chrY expression signature^39^ further revealed loss of chromosome Y (LOY) in 23.5% of male patients (Supplementary Data 1, Supplementary Fig. 5a, b), including patient B123, and additional chromosome Y copy number variations. Importantly, these LOY patients showed significant upregulation of metastasis and tissue invasion pathways (Supplementary Fig. 5c). Finally, using the matched spatial transcriptome, we performed copy-number (CN) inference, quantified genome-wide CN score, identified up to seven genetic clones per tumor, deconvoluted cell type composition, and detected transcriptionally defined spatial clusters using SpatialDE2 (Fig. 2e, f, Supplementary Fig. 6, Supplementary Fig. 7, and Supplementary Data 6).

Interestingly, facilitated by the long ONT reads, we identified a high number of somatic L1 insertions (15/23 patients with at least one somatic L1 insertion; 635 total L1 insertions across patients). Somatic L1 insertions were also detectable in cfDNA in 2 of the 15 L1-positive cases (Supplementary Fig. 3d, e). For somatic L1 insertions with confidently identified source elements, we compared their source regions to known L1 source element loci, which identified two novel source L1 elements located on chromosomes 1 and 9, respectively (**Methods**; Supplementary Data 7). We also found 11 “hot” source L1 regions that either gave rise to multiple somatic L1s within the same patient (B42), or that were active in multiple patients (Supplementary Data 8). Nine of these “hot” source L1 regions overlapped with previously reported source L1s, whereas two were novel. We explored the association of clinical features with L1 insertions, finding a significant increase in L1 insertion count in high-grade patients (*p* = 0.020, two-sided Mann-Whitney U-test), but otherwise no significant clinical associations (Supplementary Fig. 8a-e). For comparison, we also display the proportion of other variants identified from the long ONT reads, as well as L1 insertion positioning in the genome (Supplementary Fig. 8f–i).

In patient B123, we further identified positional proximity (within 10 kbp), but not precise overlap, of an L1 insertion and ecDNA segment breakpoint with a kataegis mutational event (likely APOBEC3B-mediated, given the C > T transition), proximal to a replication origin site (Fig. 2c). We validated somatic L1 insertion activity by immunofluorescence analysis of L1 ORF1p expression and ecDNA structures by FISH and immunofluorescence analysis of oncogene expression on consecutive tissue sections, respectively (Fig. 2g, and Supplementary Fig. 9, 10). These results led us to further explore the putative role of initiating events that might contribute to L1 mobilisation.

We next investigated the potential impact of microbial species on bladder cancer initiation and progression. Using the ONT data, we identified *Cutibacterium* as a common microbial species in bladder cancer tissue, as previously described; however, as *Cutibacterium* is frequent on the skin, this may potentially be a contamination (Supplementary Fig. 11a). In patient B42, however, we additionally detected reads from *Anaerococcus* (Supplementary Fig. 11b), a Gram-positive, anaerobic bacteria that is part of the normal human microbiota, but can become an opportunistic pathogen. De novo assembly confirmed the presence of bacterial DNA in the whole-genome sequencing data of this sample, and the matched RNA sample also showed *Anaerococcus* reads. We then analysed 23 additional bladder cancer samples from PCAWG^40^ (BLCA-US) but did not identify any additional tumors with such a high *Anaerococcus* abundance. Hence, microbial infection does not seem to be a frequent driver of L1 mobilization.

### L1 elements are activated via demethylated promoters and associated with genomic instability, including ecDNAs

**Fig. 3:**
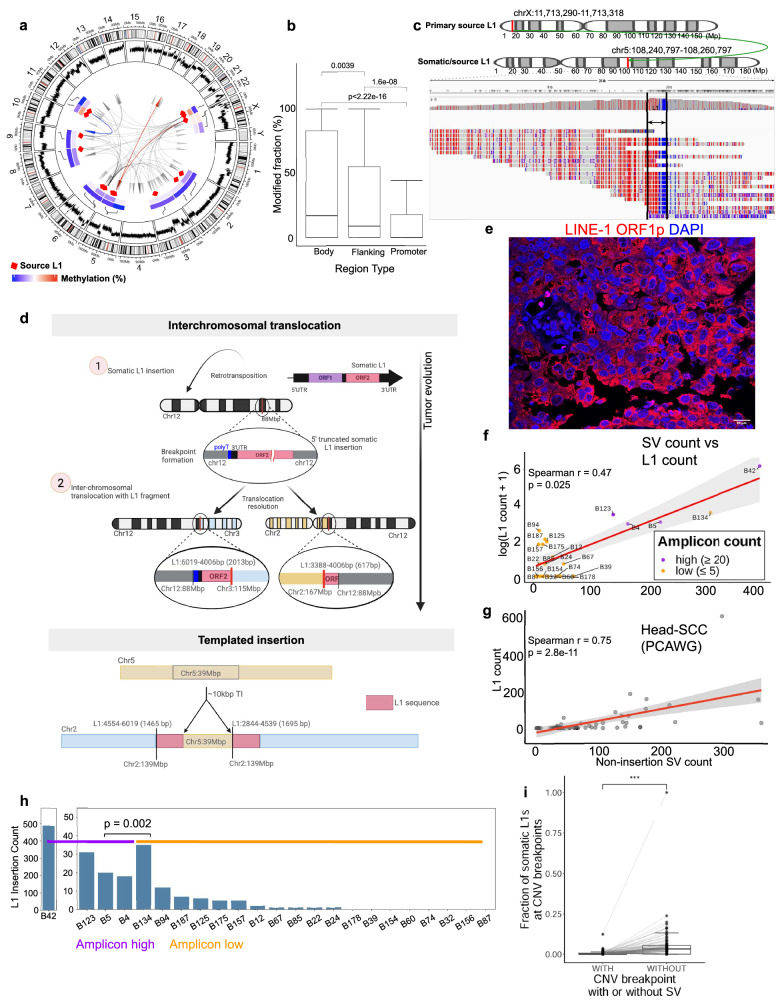
L1 element activation via demethylated promoters and association with genomic instability. **a** Circos plot of the B42 tumor. The outermost track displays genome-wide copy number alterations. The second track shows methylation levels of source L1 promoter regions and the third track methylation of their corresponding body. The innermost track highlights active L1 elements, with arrows indicating the direction of retrotransposition from the source to the target genomic location. Highlighted arrows in blue and red indicate L1 multi-jumps. **b** Methylation levels of source L1 elements across promoter (CpG *n* = 1032), body (CpG *n* = 2631), and 500 bp flanking regions (CpG *n* = 442). Box plots show the median (center line), interquartile range (box; 25th–75th percentiles), and whiskers extending to 1.5× the interquartile range; outliers beyond the whiskers are not shown. Pairwise comparisons were performed using one-sided Mann–Whitney U tests. **c** Example of a multi-jump L1 event detected in B42, visualized in IGV. The 2-color 5mC IGV mode visualizes unmethylated bases in blue and 5-Methylcytosine (5mC) in red (created with bioRender and IGV). **d** Examples of a somatic L1 insertion embedded between somatic structural variant (SV) breakpoints of interchromosomal translocation and templated insertion (created with bioRender). **e** Validation of LINE-1 expression by staining for Long interspersed element 1 (LINE-1) open reading frame 1 protein (ORF1p) on matched tissue sections. The experiment was repeated with similar results in four more patients (one section per patient). **f** Correlation between the number of L1 insertions (log-scaled) and non-insertion SVs across all samples in the bladder cancer cohort (*n* = 23 patients). Each data point shows one tumor sample, linear regression fit (red) and 95% confidence interval (gray band) are also shown. Association between variables was assessed using a two-sided Spearman rank correlation test. **g** Correlation between the number of L1 insertions and non-insertion SVs across PCAWG for Head-SCC (*n* = 57 patients). Each data point shows one tumor sample, linear regression fit (red) and 95% confidence interval (gray band) are also shown. Association between variables was assessed using a two-sided Spearman rank correlation test. **h** Counts of L1 elements across tumors. Amplicon-high tumors (*n* = 4 patients) are enriched for L1 insertions versus amplicon-low tumors (*n* = 19 patients) (*p* = 0.002, Rank-biserial effect size = 0.921, one-sided Mann-Whitney U-test). **i** Classification of copy-number variant breakpoints as SV-explained or SV-unexplained and their overlap with somatic L1 insertions in PCAWG (*p* < 0.001, paired Wilcoxon test). Source data are provided as a Source Data file.

We next turned to investigate the regulation of L1 activation and genome integration. We first analysed the ONT-derived DNA methylation data of the patient with the highest number of somatic L1 insertions, namely B42 (Fig. 3a). One mechanism for L1 activation is via promoter hypomethylation, as previously suggested in the literature^41^. We found that methylation levels were significantly lower at the L1 promoters compared to the bodies of source L1s (*p* = 4.48e-36, Rank-Biserial correlation, effect size = 0.207, one-sided Mann–Whitney U test; Fig. 3b). As a comparison, in patient B60, who showed no L1 activity, the same L1 source loci displayed methylated promoters, supporting the association between promoter hypomethylation and retrotransposition activity (Supplementary Fig. 12a). In some cases, L1 insertions transduce adjacent DNA sequences from their source element to the insertion site, allowing the origin of a somatic L1 insertion to be inferred. Using this approach and the long-read data, we performed de novo assembly for L1 elements and identified two instances where a newly inserted L1 served as the source for additional insertions. In one of these cases, the source element in GRCh38 could be clearly traced (Fig. 3c, and Supplementary Fig. 12b). This also prompted us to investigate the promoters of all full-length somatic L1 insertions, an analysis that revealed low overall methylation levels, supporting the notion that even somatic L1 insertions retain their retrotransposition competence in B42 (Supplementary Fig. 12c,** Methods**).

Somatic L1 insertions in B42 appear to be randomly distributed throughout the genome without any apparent clustering (Supplementary Fig. 12d). However, we also found evidence that a subset of somatic L1 insertions is directly associated with SV breakpoints, with L1 fragments embedded at the breakpoint junctions. These intricate SV events generate complex breakpoint patterns that are not directly mapped by conventional SV and mobile element callers; therefore, we developed an algorithm called Breaktracer to resolve such events genome-wide (**Methods**). For example, using Breaktracer, we found an inter-chromosomal translocation of chr2 and chr12 with a 617 bp long L1 fragment at the junction and another inter-chromosomal translocation of chr12 and chr3 with a 2013bp long L1 fragment - where both SVs shared the same breakpoint on chr12 (Fig. 3d). Independent local assembly and mapping the L1 fragments to a canonical L1 element revealed adjacent L1 segments (Supplementary Fig. 13a), suggesting a common somatic L1 source element on chr12 for these two L1 fragments embedded at the inter-chromosomal breakpoint junctions. Therefore, we likely observe the genomic scars of a somatic 5’-truncated L1 insertion, which subsequently mediated the occurrence of two inter-chromosomal translocations, both originating from the same somatic L1 locus on chr12. We subsequently detected a templated insertion between chr11 and chr7 in which internal L1 ORF2-derived segments likely originating from the same L1 element flanked both breakpoint junctions. Additional examples of such complex structural variants included an interchromosomal translocation between chromosomes 4 and 13 with a 450 bp L1 fragment at the breakpoint in patient B5; an inverted duplication with L1 sequences at the breakpoint on chromosome 7 in patient B123; and additional deletions, tandem duplications and templated insertions which accompanied L1 sequence fragments of varying lengths in patient B42 (Supplementary Fig. 12e, and Supplementary Fig. 13b).

We next extended our L1 analyses to the full cohort, where we detected a broad variation in the number of somatic L1 insertions between patients (0 to 490; Supplementary Data 1). To validate the L1 activity beyond the promoter methylation status, we stained for L1 open reading frame 1 protein (ORF1p) on matched tissue sections (Fig. 3e). We observed a correlation between ORF1p protein expression (signal intensity) and the number of L1 insertions detected using ONT (*p* = 0.05, r = 0.81, Spearman correlation; Supplementary Fig. 14a), in agreement with occasionally extensive somatic L1 activity in bladder cancer samples. Finally, we applied the L1EM tool^42^ (LINE-1 Expectation–Maximization, software tool designed to quantify expression of individual L1 retrotransposon loci from RNA-seq data) and found that samples with extensive somatic L1 insertions also display pronounced transcriptional activation of L1 elements (Supplementary Fig. 14b, c).

As we identified somatic L1 insertions at SV breakpoints in B42, we wanted to explore whether L1s were significantly associated with markers of genomic instability across the cohort. We found a linear relationship between the count of non-insertion structural variants and somatic L1 insertions across tumors (*p* = 0.025, r = 0.47, Spearman correlation; Fig. 3f). Notably, this relationship was also evident in the PCAWG cohort, where several tumor types showed strong associations between L1 insertions and structural variants. Head-SCC exhibited the strongest correlation (r = 0.75, p = 2.8e-11, Spearman correlation; Fig. 3g), followed by other tumor types such as Prost-AdenoCA (r = 0.42, *p* = 1.2e-09, Spearman correlation; Supplementary Fig. 14d, i). Finally, we showed a significant enrichment of somatic L1 insertions near copy-number variant (CNV) breakpoints lacking a corresponding somatic SV, as compared with breakpoints that have a matching SV in PCAWG (Fig. 3i), implying that somatic L1 fragments positioned at CNV breakpoints hinder short-read sequencing from resolving such complex rearrangements (Supplementary Fig. 14e). This pattern is consistent across multiple cancer types beyond bladder cancer in PCAWG (Supplementary Fig. 14j), indicating that somatic L1 activity contributes to shaping the structural variation landscape of cancer genomes.

Furthermore, tumors with high amplicon counts (≥ 20 amplicon regions) showed frequent somatic L1 insertions (Fig. 3h, *p* = 0.002, Rank-Biserial correlation (effect size) = 0.921, one-sided Mann-Whitney U-test). This trend was also observed in tumors with ecDNA, although without statistical significance, likely due to limited power from small sample size (p = 0.0538, Rank-Biserial correlation (effect size) = 0.429, one-sided Mann-Whitney U-test; Supplementary Fig. 14f). We further investigated this association in the TCGA cohort, using public TCGA L1 expression data^43^ and ecDNA status assignments^44^. We identified a positive correlation between patient amplicon count and L1 retrotranscriptional burden, a correlation that was modestly stronger in ecDNA-positive patients (ecDNA-positive: *ρ* = 0.63, *p* = 2.59e-4, ecDNA-negative: *ρ* = 0.34, *p* = 6.88e-3; Supplementary Fig. 14g). This pattern was exemplified by patient B123, which harbored high numbers of L1 insertions, amplicons, and multiple distinct ecDNA structures. Immunofluorescence analysis identified cells exhibiting both high *E2F3* (an oncogene present on ecDNA in B123) and L1 ORF1p expression (Supplementary Fig. 9g, and Supplementary Fig. 10). We also observed a modest enrichment of active L1 loci expression in ecDNA-positive TCGA patients, though this trend was also statistically insignificant (p = 0.087, one-sided Mann-Whitney U-test; Supplementary Fig. 14h).

Previously, it has been suggested that L1s may become transcriptionally active when present on ecDNA structures^28^. To assess whether the L1-ecDNA trends we observed might be due to the presence of L1 insertions on ecDNA structures, we explored insertions that overlapped with ecDNA regions: we found one such example in sample B42 (chr1:152430087) and one in sample B123 (chr6:21593295). We searched for highly amplified read support of these insertions, which would suggest their presence on ecDNA (**Methods**). In sample B42, the insertion support ratio was 5:23 (i.e., 5 reads supporting the insertion and 23 not supporting the insertion). In B123, the insertion support ratio was 7:81 (i.e., 7 reads supporting the insertion and 81 reads not supporting the insertion). Thus, we concluded there were no detectable L1 insertions on ecDNA in our samples, or that any such events occurred late in ecDNA evolution as subclonal somatic insertions.

Finally, we performed an integrated haplotype-resolved analysis of promoter methylation, SNVs, and RNA expression and identified two samples (B94 and B187) in which variant-containing haplotypes showed significant promoter hypomethylation (q <.05) and associated changes in gene expression (Supplementary Fig. 15). Thus, L1 insertions in our cohort as well as in external bladder and pan-cancer datasets were significantly associated with global genomic instability (i.e., complex SV rearrangements, high counts of SVs, amplicons and ecDNA presence). To further explore this relationship, we next performed a detailed spatial analysis of the most genomically unstable samples - those with ecDNA.

### Identification of ecDNA-enriched spatial regions reveals ecDNA-specific transcriptional patterns

**Fig. 4:**
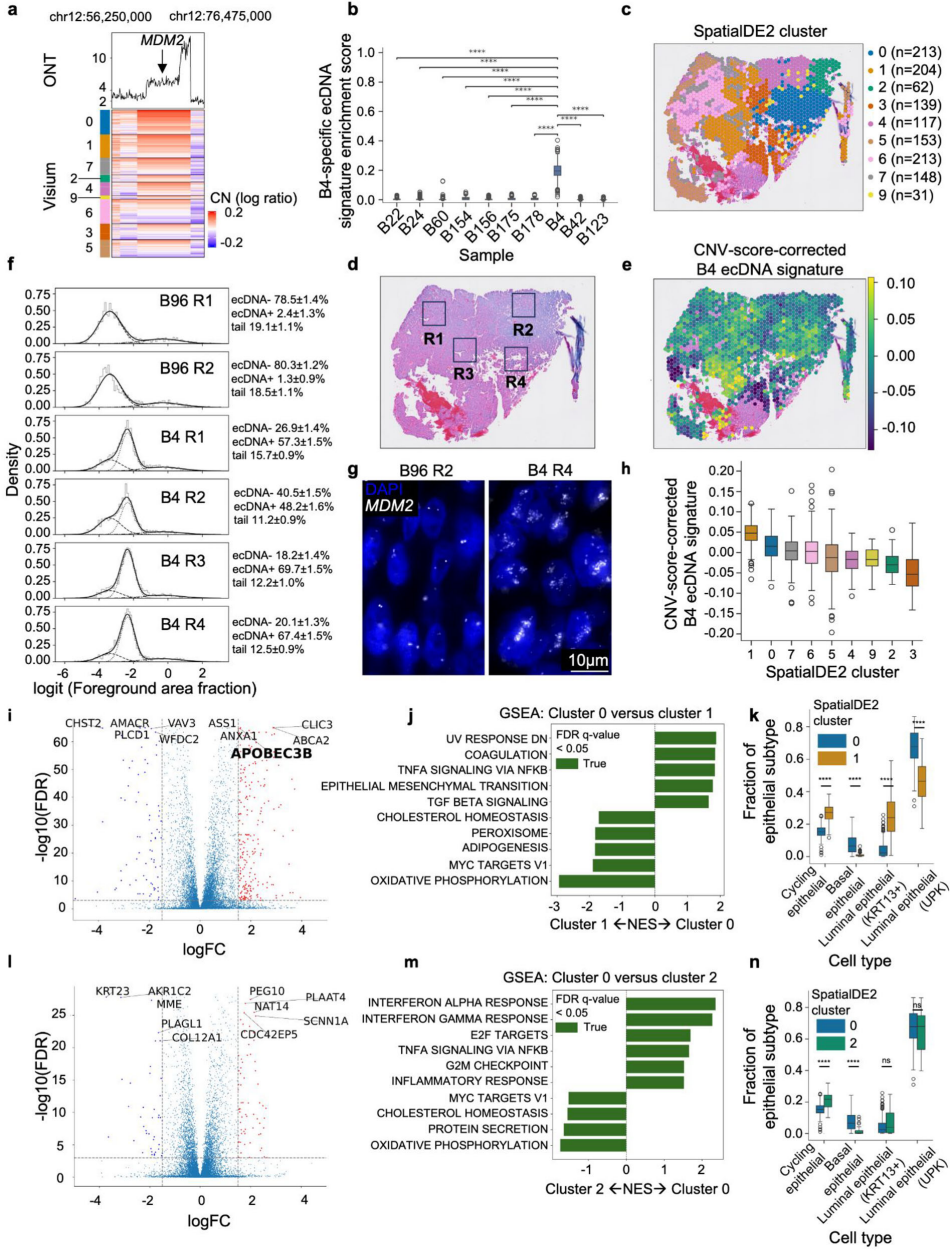
Identification of ecDNA-enriched spatial regions reveals variable APOBEC3B dosage. **a** Detection of ecDNA-associated amplicons using CNAs inferred from ONT (top) and Visium (tumor B4) for the genomic region chr12:56,250,000–76,475,000 (including *MDM2*). The left annotation shows transcriptionally defined spatial clusters from SpatialDE2 (see **c**). **b** AUC enrichment scores of the B4-specific gene set consisting of highly expressed genes located on ecDNA, computed per aneuploid spot across all 10 Visium samples. Statistical testing: Kruskal–Wallis followed by Dunn’s test with Benjamini–Hochberg correction. Aneuploid spot counts — B22: 504; B24: 1,171; B60: 505; B154: 2,525; B156: 1,826; B175: 1,102; B178: 1,256; B4: 1,284; B42: 1,037; B123: 1,507. **c** SpatialDE2 spatial clusters representing transcriptionally similar regions (spot counts indicated). **d** H&E-stained section of B4 with four annotated regions used for ecDNA quantification by FISH on an adjacent section. **e** B4 ecDNA signature enrichment score in space (CNV-score-corrected). **f** *MDM2* FISH signal quantification showing logit-transformed foreground area fraction distributions for negative control B96 (R1, R2) and the four B4 regions from (**d**) with fitted Gaussian mixture model (GMM). Components correspond to ecDNA-negative nuclei, ecDNA-positive nuclei, and a tail corresponding to thresholding artefacts. Mixture proportions (mean ± SD across 100 bootstrap iterations) are shown per region. **g** Representative FISH images for B96 (R2) and B4 (R4). **h** B4 ecDNA signature enrichment scores (CNV-score-corrected) across aneuploid spots in B4, stratified by SpatialDE2 cluster and ordered by median score (spot counts indicated in **c**). **i** Volcano plot of differentially expressed genes (DEGs) between clusters 0 and 1 from **c**. **j** GSEA on hallmark gene sets based on DEGs from **i**. **k** Epithelial cell subtype abundance comparison between ecDNA-enriched clusters 0 (*n* = 213 spots) and 1 (*n* = 204 spots); two-sided Mann–Whitney U-test with Benjamini–Hochberg correction. **l** Volcano plot of DEGs between clusters 0 and 2. **m** GSEA on hallmark gene sets based on DEGs from **l**. **n** Epithelial cell subtype abundance comparison between clusters 0 (n = 213 spots) and 2 (*n* = 62 spots); same statistical approach as (**k**). All boxplots show median, interquartile range (IQR), and whiskers extending to 1.5× IQR.

We observed evidence for greater genomic instability in ecDNA-positive samples also in the Visium data, including elevated CNV scores (*p* < 0.0001, Rank-Biserial correlation (effect size) = 0.6, two-sided Mann-Whitney U-test; Supplementary Fig. 6b) and higher counts of genetic clones (*p* = 0.12, Rank-Biserial correlation (effect size) = 0.714, two-sided Mann-Whitney U-test; Supplementary Fig. 6c). Using ONT-defined ecDNA structures as ground truth (initially focusing on patient B4 due to its high tumor content), we confirmed that the corresponding genomic loci could be detected in Visium data using CopyKAT, providing an orthogonal estimate of ecDNA-associated copy-number alterations (Fig. 4a). We observed that transcriptional signature-based estimates of ecDNA enrichment were more robust across samples and spatial regions compared to CNV inference (data not shown). We therefore developed an approach to identify ecDNA-enriched regions within the tissue architecture in spatial transcriptomics data (see **Methods** and Supplementary Fig. 16a), by establishing patient- and ecDNA-specific transcriptional signatures to estimate ecDNA enrichment from Visium data (Fig. 4b). Applying the ecDNA detection workflow to tumors B4 (Fig. 4, and Supplementary Figs. 16, 17), B42 (Supplementary Fig. 18), and B123 (Supplementary Fig. 19), which all harbored ecDNAs, revealed that sample-matched ecDNA signatures (e.g., B4 signature in sample B4) enable detection of ecDNA-associated transcriptional signals in each individual sample. Hence, beyond bladder cancer, this new approach will help understanding ecDNA biology and studying ecDNAs in their spatial context, by linking ecDNA levels across subsets of tumor and non-tumor cells as well as across distinct spatial clusters.

In our cohort, applying this method for detailed spatial analysis of ecDNA in B4 revealed notable patterns to potentially explain the link between L1 insertions and ecDNA. SpatialDE2-defined clusters showed a broad range of ecDNA enrichment scores, reflecting spatial variation in the fraction of ecDNA-positive cells, as confirmed by FISH (Fig. 4c-h, Supplementary Fig. 16b-g). CNV scores, used here as a proxy for tumor purity, also varied across clusters, with clusters 0 and 2 exhibiting the highest CNV scores, consistent with a high tumor cell fraction, while cluster 9 showed the lowest CNV score (Supplementary Fig. 16h). Cell type deconvolution further supported these differences in CNV scores, as most clusters were composed of more than 90% epithelial cells, while cluster 9 displayed an increased abundance of fibroblasts and immune cells (Supplementary Fig. 17d). Between the spatial areas with the highest enrichment of ecDNAs (clusters 0 and 1 for patient B4, respectively), we detected significant differences in the fractions of cell types and in the respective transcriptomes, with *APOBEC3B* as one of the most differentially expressed genes between these two ecDNA-high clusters (Fig. 4i–k, and Supplementary Fig. 17d-e). Notably, *APOBEC3B* expression was the highest in cluster 0 compared to all other SpatialDE2 clusters, and overall, sample B4 showed the highest *APOBEC3B* expression among all Visium samples, highlighting its cluster- and sample-specific upregulation (Supplementary Fig. 17a, b). GSEA analysis on hallmark gene sets identified TGF-alpha signaling, epithelial mesenchymal transition, and TGF-beta signaling as upregulated in cluster 0 (*APOBEC3B*-enriched) and oxidative phosphorylation and MYC targets as upregulated in cluster 1 (*APOBEC3B*-relatively reduced), respectively (Fig. 4j). Importantly, APOBEC3B has previously been reported to be activated in the presence of L1 insertions to serve as an inhibitor of L1 retrotransposition^22,45^, and its function as an antiviral cytidine deaminase suggests a viral mimicry response mechanism. We hypothesize that enriched L1 retrotransposition activity within the *APOBEC3B*-enriched ecDNA-positive cluster 0 cells versus the *APOBEC3B*-relatively reduced ecDNA-positive cluster 1 cells might explain the differential expression of TNF-alpha and TGF-beta signaling.

More broadly, we also identified marked differences in cell type composition and biological processes when comparing ecDNA-enriched versus ecDNA-depleted clusters (Fig. 4l–n). Here, we again saw the upregulation of immune pathways including interferon alpha and interferon gamma response in the ecDNA-high cluster 0 versus ecDNA-low cluster 2. This upregulation of interferon-related pathways was also observed when comparing cluster 0 to other ecDNA-low clusters (clusters 3 and 4), indicating that cluster 0 consistently shows elevated immune pathway activity compared to ecDNA-low clusters (Supplementary Fig. 17c). Importantly, ecDNAs have been associated with a dampened immune response, suggesting an alternative mechanism responsible for activating an immune response. We hypothesize that enriched L1 retrotransposition activity within the ecDNA-high cluster 0 cells versus the ecDNA-low cluster 2 cells could potentially contribute to this re-activated immune response.

Thus, from our spatial data we detected significant genomic instability via ecDNA linked to immune response, which was further associated with APOBEC3B activity. To explore whether this result could potentially be mediated by L1s, we next sought to temporally relate L1s with genomic instability, and explore a viral mimicry response in our L1-high samples.

### Early L1 insertions associated with genomic instability, viral mimicry response, and epigenetic dysregulation

**Fig. 5:**
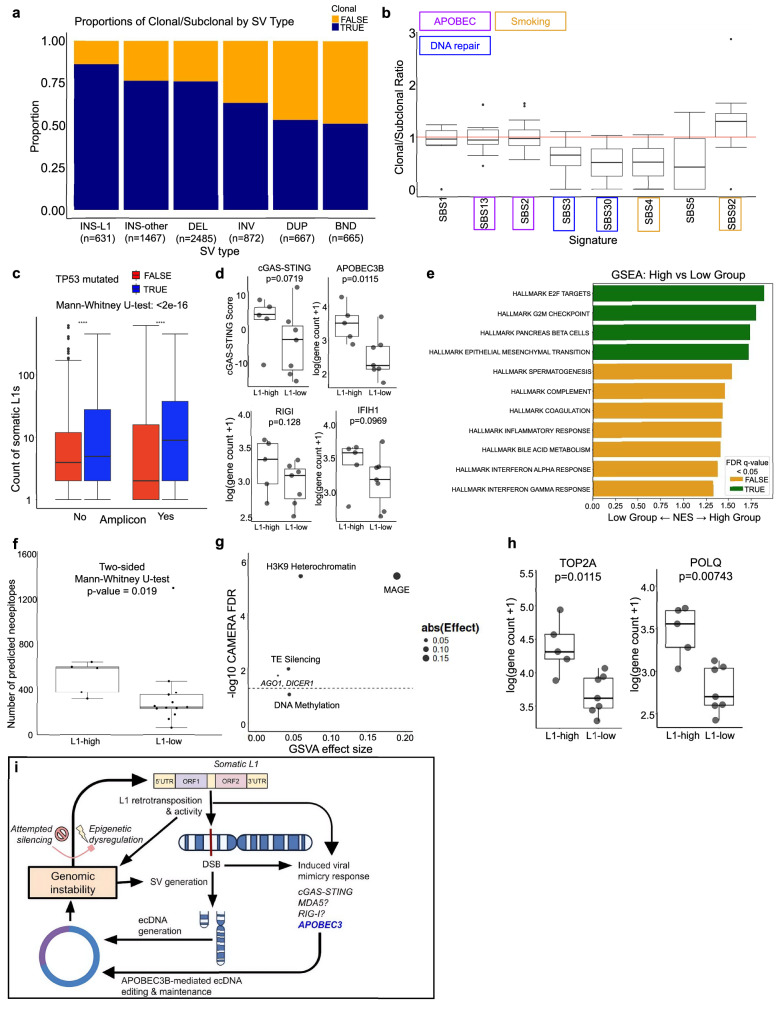
L1 insertions are clonal and correlated with mutational and DNA repair processes. **a** Proportion of clonal structural variations (VAF > 0.3) across the full cohort, separated by SV type (ALU and SVA insertion types are excluded). **b** Ratio of clonal (n = 233,373) to subclonal (n = 253,821) mutations grouped by mutational signature across the cohort. Signatures with a ratio below the red line (h = 1) suggest late (subclonal) mutational events, while those above the line indicate early (clonal) events. **c** Log-scaled count of L1 insertions in the PCAWG dataset, stratified by amplicon status (Yes, *n* = 1753; No, *n* = 826) and *TP53* mutation status (True, *n* = 293; False, *n* = 2,286). Box plots show the median (center line), interquartile range (box; 25th–75th percentiles), and whiskers extending to 1.5× the interquartile range; outliers beyond the whiskers are shown as points. Statistical comparisons were performed using two-sided Mann–Whitney U test; significance levels are indicated in the figure. **d** Expression of viral mimicry response pathway genes in L1-high versus L1-low samples, defined by insertion count thresholds (L1-high = insertion count > 15: *n* = 5 patients, L1-low = insertion count <5: *n* = 7 patients). Significance assessed with one-sided Mann-Whitney U-test; see text for Rank-Biserial coefficients (effect sizes). **e** Gene set enrichment analysis (GSEA) on hallmark gene sets of L1-high versus L1-low samples, defined by insertion count thresholds (L1-high = insertion count > 15: n = 5 patients, L1-low = insertion count <5: *n* = 7 patients). Sets with nominal *p* value < 0.05 are displayed on the plot. Significance assessed by FDR q-value < 0.05 (green). **f** Predicted neoepitope counts from single nucleotide variants and small insertions/deletions from tumor samples classified as L1-high (insertion count > 15, *n* = 5 patients) and L1-low (insertion count <5, *n* = 13 patients). Statistical significance was evaluated using a two-sided Mann-Whitney U-test. All boxplots in Fig. 5 show the median, interquartile range (IQR), and whiskers extending to 1.5× IQR. **g** Integrated gene set enrichment analysis comparing gene sets between L1-high and L1-low samples. Gene Set Variation Analysis (GSVA) was used to quantify gene set activity and CAMERA to evaluate statistical significance, showing effect size estimates from GSVA on the x-axis and -log10 transformed FDR estimates from CAMERA on the y-axis between the L1-high and L1-low groups. **h** Expression of ecDNA-replication-induced double strand break response genes in L1-high versus L1-low samples, defined by insertion count thresholds (L1-high = insertion count > 15: *n* = 5 patients, L1-low = insertion count <= 5: *n* = 7 patients). Significance assessed with one-sided Mann-Whitney U-test; see text for Rank-Biserial coefficients (effect sizes). **i** Graphical abstract of results and hypotheses (chromosome and ecDNA schematic images from bioRender). Source data are provided as a Source Data file.

To get insights into the temporal genomic evolution of the tumors, we first dissected clonal and subclonal events (**Methods**). Somatic L1 insertions were mostly clonal in this cohort (Fig. 5a), as were the clock-like mutational signatures (Fig. 5b). In contrast, signatures of DNA repair (SBS3, SBS30) were largely subclonal. We also detected the tobacco-smoking–associated mutational signature SBS4 predominantly in subclonal mutations, whereas another smoking-associated signature, SBS92, appeared largely clonal. (Fig. 5b, and Supplementary Fig. 20a, b). Given the reported association between *TP53* mutations and somatic L1 insertions^46,47^, including via an enhanced viral mimicry response pathway^48^, we also sought to explore possible relative timing between the events. As expected, *TP53*-mutant tumors showed significantly higher numbers of L1 insertions as compared to *TP53*-wild-type tumors (p < 2e-16, Rank-Biserial correlation (effect size) = 0.119, one-sided Mann-Whitney U-test, Fig. 5c). Of the 9 *TP53* mutation events identified, 7/9 were clonal (~78%, Supplementary Data 9). These results indicate *TP53* mutation and L1 insertion as an early, often clonal event, followed by other mutational processes including DNA repair and APOBEC-mediated editing, and a potentially enhanced viral mimicry response.

To further explore the viral mimicry reaction suggested by our spatial transcriptome analyses, we next compared the expression of viral infection response pathway genes between our L1-high (L1 insertion count > 15, *n* = 5 with RNAseq data; B42, B123, B134, B4, B5) and L1-low samples (L1 insertion count <5, *n* = 7 with RNAseq data; B24, B154, B178, B39, B60, B156, B74) (Fig. 5d). The cGAS-STING score, a marker for viral infection response to cytosolic dsDNA, was increased in L1-high patients, although the difference did not achieve statistical significance with the available cohort size (*p* = 0.0719, Rank-Biserial correlation (effect size) = 0.543, one-sided Mann-Whitney U-test). *APOBEC3B* expression was significantly increased in the L1-high patients (*p* = 0.0115, Rank-Biserial correlation (effect size) = 0.829, one-sided Mann-Whitney U-test), supporting our previous spatial results. *RIG-I* and *MDA5* (*IFIH1*) expression—markers of the viral response to cytosolic dsRNA—were both increased in L1-high patients; even though the differences were not statistically significant at this sample size (*p* = 0.128, Rank-Biserial correlation (effect size) = 0.429, and *p* = 0.0969, Rank-Biserial correlation (effect size) = 0.486, respectively; one-sided Mann-Whitney U-test). To support these findings, we ran GSEA on the L1-high versus L1-low samples (Fig. 5e). We found L1-high enrichments of immune response pathways (inflammatory response, complement signaling, Interferon gamma response, Interferon alpha response) and cell growth/cell cycle progression gene sets (E2F targets, G2M checkpoint, epithelial-to-mesenchymal transition). In line with this, we also detected significantly more (*p* = 0.019, two-sided Mann-Whitney U-test) predicted neoepitopes in L1-high as compared to L1-low patients (Fig. 5f). Taken together, these results suggest an immune response to early, clonal L1 insertions.

Further gene set enrichment analysis between our L1-high (L1 insertion count > 15, *n* = 5 with RNAseq data; B42, B123, B134, B4, B5) and L1-low samples (L1 insertion count <5, *n* = 7 with RNAseq data; B24, B154, B178, B39, B60, B156, B74) showed an up-regulation of gene sets associated with H3K9 heterochromatin formation and transposable element silencing, including elevated expression of the H3K9 methyltransferases SUV39H1 and SUV39H2 (Fig. 5g, and Supplementary Fig. 20c–e). These results are consistent with engagement of compensatory epigenetic defense mechanisms in response to the previously identified increased retrotransposon activity (Supplementary Fig. 14b, c). Despite this response, canonical heterochromatin-repressed loci, most notably MAGE family cancer–testis antigens, were also robustly derepressed in L1-high samples, indicating functional insufficiency of H3K9-mediated silencing (Fig. 5g, Supplementary Fig. 20c, and Supplementary Fig. 20f). We also detected increased expression of *APOBEC3B*, a cytidine deaminase induced by DNA damage and replication stress and implicated in the mutational diversification of ecDNA. We further note trends toward increased expression of *AGO1* and *DICER1* (Supplementary Fig. 20c), suggesting partial engagement of RNA-mediated surveillance pathways in response to abundant repeat and retrotransposon transcripts. These findings support a model in which EMT-associated chromatin relaxation permits elevated L1 transcription, leading to increased DNA damage and APOBEC3B activation, thereby promoting genomic fragmentation and circularization events that underlie ecDNA formation. Indeed, evidence of the downstream impacts of ecDNA also exists in our cohort; we detected significant L1-high-enrichment of a topoisomerase (*TOP2A*) previously identified to support ecDNA replication-induced DNA double-strand breaks^49^ (p = 0.0115, Rank-Biserial correlation (effect size) = 0.8286, Fig. 5h). We further found significant L1-high-enrichment of the *POLQ* gene implicated in the repair of these double-stranded breaks through the alternative non-homologous end joining (alt-NHEJ) pathway^49^ (p = 0.0074, Rank-Biserial correlation (effect size) = 0.8857, Fig. 5h). The simultaneous induction, but apparent functional inadequacy of multiple epigenetic and RNA-based silencing mechanisms, highlights a state of persistent genomic stress linking L1 activity, epigenetic dysregulation, and ecDNA biology (Fig. 5i).

## Discussion

Our comprehensive multi-omic profiling of 48 bladder cancers revealed diverse patterns of genomic instability. Integrated analysis of copy-number and single-nucleotide variants revealed recurrent alterations in established bladder cancer driver genes, including deletions in *KDM6A* and *STAG2*, as well as high-frequency missense mutations in *NBPF10*. The long-read sequencing approach pursued in our study furthermore identified widespread structural variants and ecDNA, and particularly facilitated the discovery of somatic L1 insertion events across samples. These results were validated and further explored through spatial transcriptomics analysis, which identified ecDNA-specific transcriptional signatures within the tissue architecture and marked differences between ecDNA-enriched tumor regions, potentially linked with differential APOBEC activity. Timing analysis identified L1 insertions as an early, clonal event possibly driven by an EMT-associated relaxed chromatin state, and our integrated analysis suggested genomic instability and viral mimicry response as downstream results of L1 activity.

In agreement with previous literature, clonal and subclonal mutation timing analyses showed L1 insertions to be mostly clonal^50^ and associated with *TP53* mutations^22^. Using long-read sequencing and de novo assembly, we identified active source elements and multi-jump L1 insertions, suggesting ongoing retrotransposition activity in tumors with hypomethylated L1 promoters. In contrast, the most confident L1-silent tumor in the cohort displayed methylated promoter regions at the same loci, reinforcing the role of epigenetic regulation. While L1 insertion length did not correlate with methylation status, we observed inter-patient variability in insertion burden, which aligned with ORF1p protein expression in tissues. Samples with extensive L1 insertions also showed strong transcriptional activity of L1 elements. These results confirmed the early and active nature of L1 insertions in our samples.

We first explored whether this activity was caused by microbial infection, however, this does not seem to be a frequent driver of L1 mobilization. Nevertheless, our data suggest the possibility of a putative role for microbial infection as a potential driver of L1 mobilization. In line with this, Sheng et al.,^51^ showed that *Anaerococcus* - among other genera - is enriched in the urine of bladder cancer patients as compared to controls. Even though not a frequent event, *Anaerococcus* infection may contribute to carcinogenesis by inducing reactive oxygen species (ROS) production^52^. These reactive oxygen species (ROS) can cause hypomethylation of L1 elements in bladder cells^53^, potentially triggering the large number of somatic L1 insertions (490) in B42.

We next identified multiple potential consequences of L1 retrotransposition in our patients. First, we showed an association between L1 insertions and the presence of genomic instability markers (including structural variants, amplicons, and ecDNA) in bladder cancer. Although direct evidence between L1 insertions and structural variant formation, especially ecDNA formation, is currently limited, several observations imply possible connections. First, L1 retrotransposition can cause genomic instability through insertions promoting double-stranded DNA breaks^54^. This instability might facilitate formation of SVs and downstream ecDNAs; indeed, we observed evidence of several L1 fragments inserted at an SV breakpoint, and additional examples may potentially exist that are not yet detectable due to current sequencing technology and algorithmic limitations. While we did not find evidence for L1 insertions at ecDNA breakpoints, we identified a significant association between L1 count and ecDNA presence across multiple cohorts. L1-high samples also showed a significant enrichment in genes known to support ecDNA replication, suggesting the downstream activity of ecDNA in L1-high samples. Indeed, both L1 and ecDNA activity are expected in genomically unstable tumors, but a causative relationship between L1 insertion and ecDNA formation is conceivable and should be explored in more detail. For example, a relationship between L1 insertions and the formation of breakage-fusion-bridge cycles, a common precursor to ecDNA, has been established in the PCAWG cohort^54^. In addition, ecDNA has been shown to function as mobile enhancers, interacting with chromosomal DNA to amplify gene expression^55^. These interactions could potentially include genes affected by L1 insertions, as previously described in a cancer context^41^, leading to coordinated regulation and amplification of specific genomic regions. Our results, combined with previous findings, suggest a model whereby L1 insertions and ecDNA may arise independently as simultaneous results of epigenetic dysregulation and genomic instability, but may also interact with one another, with L1 insertions potentially resulting in precursor DNA rearrangements needed for ecDNA formation.

A second possible consequence of L1 retrotransposition is an effect on APOBEC3B activity. APOBEC3B has been previously shown to be activated in response to L1 retrotransposition as part of an innate viral response^45^. In our dataset, *APOBEC3B* expression was significantly elevated in tumors with high L1 insertion counts. Based on this, we hypothesize that APOBEC3B might serve as a mechanistic link between L1s and ecDNAs. Supporting this hypothesis, a previous study identified co-occurrence of APOBEC3-associated kataegis and ecDNA - referred to as *kyklonas* - in 31% of tumors with ecDNA^30^. Notably, *kyklonas* were observed at a particularly high frequency in patients with Urothelial Bladder Carcinoma^56^. While this would suggest that *APOBEC3B* might be uniformly expressed across ecDNA positive spatial regions, our analysis revealed that *APOBEC* was among the most differentially expressed genes between regions positive for ecDNA. One possibility to explain differences in *APOBEC* expression may be mediated by differential L1 retrotransposition activity. In particular, because we identified *APOBEC*-related signatures in our cohort, we propose a mechanism whereby early L1 insertions trigger an APOBEC3B response, which in turn support the editing and maintenance of ecDNA (the existence of which may potentially also be linked to L1 activity, as previously described in this discussion, and by other studies^22–29^). We note that this mechanism is distinct from the previously observed APOBEC-ecDNA axis, whereby APOBEC3B functions after ecDNA formation to support ecDNA mutagenesis and evolution^30^. We hypothesize that these mechanisms may exist independently or synergistically, varying by cancer type and sample; both should be considered and studied in further detail to understand whether APOBEC may act as both an instigating event in ecDNA formation and a post-formation mechanism for ecDNA maintenance.

Finally, we found that enriched *APOBEC* expression was just one component of a third potential consequence of L1 retrotransposition; an induced viral mimicry response. Significant overexpression of the *APOBEC3B* gene, alongside the cGAS-STING pathway^32^ and *RIG-I* and *MDA5* genes^31^, corroborates the viral mimicry response taking place in the L1-high samples. This is further supported by GSEA results, which show the significant overexpression of the interferon-alpha response pathway in L1-high samples. Hence, we suggest a link between L1 retrotransposition activity, genomic instability and the viral mimicry response, highlighting the value of L1s as a potential biomarker for bladder cancers with complex genomes.

These hypotheses of the downstream effects of L1 insertion should be further explored in future studies to clarify mechanistic relationships. Importantly, alternative explanations unrelated to L1 such as chronic inflammation, bacterial contamination, or APOBEC-driven dsDNA release should also be considered. We hypothesize the following model (Fig. 5i), focusing on genomic instability: L1s become demethylated and thus activated through a state of genomic instability, possibly driven by EMT-associated chromatin relaxation. L1 insertions then trigger downstream events including DSBs, APOBEC3B activity, and a viral mimicry response. DSBs further contribute to genomic instability by leading to SV generation and ecDNA formation. APOBEC3B activation may result in further maintenance and editing of ecDNA in these samples. The viral mimicry response may also be further amplified by recognition of ecDNA replication-associated DNA damage. Each of these responses in turn amplifies the state of genomic instability, as do unsuccessful attempts at epigenetic and RNA-based silencing mechanisms.

To the best of our knowledge, this is the first study of bladder cancer that integrates long-read DNA sequencing of tumors, short-read DNA sequencing of blood-based cfDNA, RNA sequencing, and spatial transcriptomics. This characterization of the genetic, epigenetic, transcriptomic, and spatial features of bladder cancer identified critical factors contributing to disease aggressiveness and suggested potential disease progression mechanisms, which will need further research to ultimately improve diagnosis, prognosis, and treatment for bladder cancer. In particular, we revealed evidence for previously suggested downstream effects of L1 retrotransposition. Our work highlights the value of L1 elements as a putative biomarker in bladder cancer and offers evidence for potential mechanisms of disease progression via L1 insertion-mediated genomic instability.

### Limitations and future work

Several aspects can be explored deeper in the future. Further mechanistic experiments including time-course studies and modulation of L1 activity will be required to formally address causality and to study the role of heterochromatin, epigenetic dysregulation and viral mimicry. Our framework for inferring ecDNA enrichment in space cannot formally distinguish between different underlying cellular configurations, such as a larger fraction of cells carrying ecDNA versus a smaller fraction of cells harboring higher ecDNA copy number. Moreover, when CNV score correction substantially alters the ecDNA enrichment pattern, spatial comparisons require explicit consideration of local tumor content. In addition, transcriptome-based enrichment alone cannot differentiate between transcriptional signals originating from ecDNA and those arising from intrachromosomal amplification affecting the same genomic region without orthogonal validation. As an important side note, our analysis demonstrated important advantages of ONT sequencing for L1 discovery and epigenetic characterization, particularly in the context of complex SVs, where reads must span the SV junction and the embedded L1 fragment. However, we also frequently encountered mapping artifacts when L1s were inserted into the genome and long reads did not cover the entire insertion. This causes read aligners to map these fragments to alternative L1HS sequences present in the human genome, leading to false positive SV signatures. Therefore, the development and extension of SV discovery methods is crucial in this context to safeguard long-read SV callers against such mapping artifacts and to identify the genuine retrotransposon-linked SVs. More broadly, continued optimization of variant-calling pipelines for long-read data will be important, given that the higher Nanopore sequencing error rate and the presence of chimeric reads at low frequency can affect the accuracy of SNV, InDel and SV discovery. Despite these limitations, our work highlights the important role of L1 elements in the formation of complex SVs, underscoring the need for future studies to dissect their mechanistic contribution to these events.

## Methods

### Material and data collection

All experiments in this study involving human tissue or data were conducted in accordance with the Declaration of Helsinki. Clinical data and tissue of all patients in the study were collected after receiving written informed consent from the respective patients or their legal representatives and after approval by the ethics committee of Heidelberg University. Participants did not receive compensation and were informed of this prior to enrolment in the study. Sex and/or gender was not considered in the study design and sex and/or gender of participants was determined based on self-report. Collection of blood and tumor material from patients with bladder carcinoma at diagnosis was done in collaboration with Dr. Mladen Stankovic and his team at the Salem hospital (Heidelberg).

#### Blood collection

Blood was collected in cf-DNA/cf-RNA preservative tubes (Norgen Biotek) and mixed according to the manufacturer’s instructions. Blood tubes were collected from the Salem hospital and blood was processed immediately upon arrival at the laboratory. To separate plasma, blood tubes were centrifuged at 425xg for 20 minutes at room temperature. The upper plasma layer was carefully transferred by pipetting to clean Eppendorf tubes. Plasma aliquots were stored at −80 °C until extraction of cfDNA. The rest of the blood pellet was saved and used for isolation of white blood cells (WBC) from which germline DNA was extracted.

#### Extraction of cell-free DNA

Cell-free DNA extraction was done using Quick-cfDNA/cfRNA Serum & Plasma Kit (Cat#R1072, Zymo Research), following Parallel Purification protocol provided by the company. The standard input amount of plasma used for extraction was 2 ml. Lower amounts of plasma in exceptional cases of limited plasma material. The concentration of extracted cfDNA was measured with Qubit dsDNA High Sensitivity Assay Kit and length of cfDNA was determined by Bioanalyzer using Agilent High Sensitivity DNA Assay Kit.

#### Library construction for cell-free DNA sequencing

Library construction was performed using KAPA HyperPrep Kit (Cat#7962363001, Roche), following manufacturer’s instructions. The standard cfDNA input amounts for library construction was 2.5–5.0 ng, depending on the quantity available. The order of steps followed from the manufacturer’s protocol was the following: End Repair and A-tailing, Adapter Ligation (Adapters used: KAPA Unique Dual-Indexed Adapter Kit; adapter concentration: 750 nM; end step incubation overnight at 16 °C), Post-ligation Cleanup (step 3.14: 55 µl of Nuclease-free water used instead of the Elution buffer), Double-sided Size Selection (Appendix A1., using Ampure Xp beads Cat#A63881, instead of KAPA cleanup beads), Library Amplification, Post-amplification Cleanup. Final concentration of the libraries was checked with Qubit dsDNA High Sensitivity Assay Kit. Libraries were multiplexed and submitted for sequencing at the DKFZ NGS Core Facility. Whole-genome sequencing of cfDNA was done on Illumina NovaSeq 6000 S4 sequencing platform (100 bp, paired-end).

#### Cell-free DNA sequencing data analysis

FASTQ files were aligned to the GRCh38 reference genome with BWA-MEM (version 0.7.15)^57^. Duplicates were marked with picard tools (https://broadinstitute.github.io/picard/, version 2.25.1), which were removed in the next step in addition to low-quality reads, secondary alignments and supplementary alignments using SAMtools^58^. To enrich the tumor DNA, we only kept short fragments (90-150 bp) as reported in previous studies^59^. Next, read counts were extracted using hmmcopy_utils (https://github.com/shahcompbio/hmmcopy_utils) for 500 kb bin size. Finally, ichorCNA^60^ was used with default parameters to infer copy-number alterations and estimate the tumor fraction. One benign sample from our cohort was used as a reference. Only samples with the estimated tumor fraction above 0.03 were classified as positive.

#### Tumor tissue collection

Tumor tissue (bladder carcinoma) was collected at diagnosis of each patient, during a procedure called Transurethral Resection of Bladder Tumor (TURBT). Removed tumor material was placed in a Falcon tube containing 5 ml of RPMI medium and transported on ice to the laboratory. The material was then frozen by dipping into pre-cooled 2-methylbutan for one minute, transferred into pre-cooled 1.8 mL Thermo Scientific CryoTube Vials and stored at −80 °C until further cryosectioning.

#### Cryosectioning and tumor content estimation

In order to confirm the presence of the tumor and estimate tumor content, we cryosectioned each tumor piece and performed Hematoxylin and Eosin staining, using standard procedures. If the tumor content was ≥ 70%, further cryosections were made for DNA extraction.

#### Short-read whole genome sequencing

Extraction of DNA from cryosectioned tissue (five benign lesions and two tumor samples) was done using the DNeasy Blood and Tissue Kit (Cat#69506, Qiagen) according to the manufacturer’s instructions. Sequencing libraries were prepared using the Watchmaker DNA library preparation workflow and sequenced on an Illumina NovaSeq X platform (paired-end 150 bp reads; NovaSeq X Plus PE150) to a target depth of approximately 25 billion reads for ultra–low-input samples.

#### Extraction of DNA for long-read sequencing

Extraction of DNA from cryosectioned tumor tissue was done using Gentra Puregene Tissue Kit (Cat#158063, Qiagen), following protocol for DNA purification from tissue using the Puregene Tissue Kit. In most cases, the amount of cryosectioned tumor material was between 5–30 mg tissue, therefore we followed the extraction protocol for low input amounts. For the elution step, a low EDTA buffer was used instead of the DNA hydration solution provided in the kit. The concentration of extracted tumor DNA was measured with Qubit dsDNA High Sensitivity Assay Kit and purity is analysed with NanoDrop. DNA was stored at −20 °C until further use.

#### Fragmentation of DNA

In order to obtain optimal DNA fragment length for long-read sequencing (10-30 kb), we performed the shearing of extracted high molecular weight DNA with the Megaruptor 3 system (EMBL, GeneCore Facility). The conditions used for shearing were the following: speed=30 s, concentration=40 ng/ul, vol=130ul. Shearing of DNA was followed by 1.0X v/v cleanup with SMRTbell beads (PacBio). Concentration of sheared DNA was measured with Qubit dsDNA High Sensitivity Assay Kit. Fragment length was analysed with the Agilent Femto Pulse system using Genomic DNA 165 kb assay kit.

#### Library construction for long-read DNA sequencing

Library construction was performed using Ligation Sequencing Kit V14 (SQK-LSK114, Oxford Nanopore), following the Library prep protocol from Oxford Nanopore called “Ligation sequencing DNA V14 (SQK-LSK114)”, further selected for PromethION device. Several steps of the protocol have been altered and all the alterations are listed below.

#### DNA repair and end-prep

- Use of DNA Control Sample (DCS) was omitted and we used 48 ul of DNA (instead of 47 ul)
- Input DNA: 100-200 fmol
- Using a thermal cycler, we incubated at 20 °C for 1 hour (instead of 5 min) and at 65 °C for 10 min (instead of 5 min)
- Incubation on a Hula mixer was done for 30 min (instead of 5 min) at RT
- After resuspension of the pellet in 61 ul Nuclease-free water, we incubated for 10 min (instead of 2 min) at RT

#### Adapter ligation and clean-up

- To enrich for DNA fragments of 3 kb or longer, we used Long Fragment Buffer (LFB)
- Mix of DNA with Ligation buffer, Quick T4 DNA Ligase and Ligation Adapter was incubated for 20 min (instead of 10 min) at RT
- Incubation on a Hula mixer was done for 30 min (instead of 5 min) at RT
- After pellet resuspension in 25 ul Elution Buffer, the tube was incubated for 20 min (instead of 10 min) and at 37 °C instead of RT

#### Long-read tumor DNA sequencing

The input of the tumor DNA library used for loading on the flow cell was 20 fmol. We used R10.4.1 flow cells (FLO-PRO114M, Oxford Nanopore) and sequenced one tumor sample per flow cell. Sequencing was performed at the DKFZ Sequencing Open Lab on the PromethION (P24) device. Priming and loading of the flow cell was done according to Oxford Nanopore instructions. In order to reach optimal data output and coverage (30x), washing and re-loading of the flow cell was performed at least two times, approximately after 24 h and 48 h from the sequencing start. Total sequencing time was 96 hours.

#### Germline DNA sequencing

DNA was extracted from white blood cells using the MonarchⓇ HMW DNA Extraction Kit for Cells & Blood (NEB, #T3050L) with the Standard Input protocol. Extracted DNA was sheared with the Megaruptor 3 system (EMBL, GeneCore Facility). The conditions used for shearing were the same as for tumor DNA: speed=30 s, concentration=40 ng/ul, vol=130 µL. Shearing of DNA is followed by 1.0X v/v cleanup with SMRTbell beads (PacBio). Concentration of sheared DNA was measured with Qubit dsDNA High Sensitivity Assay Kit. Fragment length was analysed with the Agilent Femto Pulse system using Genomic DNA 165 kb assay kit. Libraries for germline DNA bulk sequencing were done with the TruSeq Nano DNA LT LPK kit (order number 20015964) and the TruSeq DNA UD Index v2 (order number 20040870) both from Illumina. Samples were sequenced on a NovaSeq sequencing instrument in paired-end mode (2 × 150bp). Germline sequencing metrics per sample are available in Supplementary Data 11.

#### Bulk RNA sequencing

RNA extraction was done with the Maxwell RSC Simply RNA Tissue Kit or with the RNeasy Kit (Qiagen) using the DNase treatment. The RNA quality was checked with a TapeStation. The library preparation was done at EMBL GeneCore. The RNA-seq libraries were prepared from total RNA with ribosomal RNA depletion using the NEBNext Ultra II Directional RNA Library Prep Kit for Illumina. Samples were multiplexed and sequenced using a NextSeq2000 sequencing instrument in paired-end mode. Depending on input RNA quality, samples were sequenced as 2x65bp (B39, B74 and B123) or 2x110bp (all other samples).

#### Long-read basecalling and alignment

Basecalling of the Nanopore sequencing data was performed with Dorado version 0.6.0 (https://github.com/nanoporetech/dorado) using the most accurate PromethION basecalling model (sup) with modified base detection of 5-methylcytosine (5mC) and 5-hydroxymethylcytosine (5hmC) in CG contexts (sup,5mCG_5hmCG model). The base called data was then aligned to the human reference genome (GRCh38 and T2T) using minimap2^61^. T2T alignment was used to observe copy-number variations on chrY; GRCh38 alignment was used for remaining analysis.

#### Long-read alignment quality control

Quality control of the long-read alignment files was done using NanoPack^62^, Alfred^63^ and verifyBamID^64^ to detect sample cross-contamination. The long read whole-genome coverage varied from 19x (B67) to 43x (B4) with an average coverage of 32x. The N50 read length varied from 11,565 bp to 19,249 bp with an average N50 read length of 15,655 bp. The estimated sequencing error rate of the aligned data was in the range of 0.94% to 1.84% with an average estimated sequencing error rate of 1.17%. ONT sequencing metrics per sample are available in Supplementary Data 11.

#### Single-nucleotide and small insertion and deletion calling

Clair3^65^, ClairS (https://github.com/HKU-BAL/ClairS) and DeepSomatic (https://github.com/google/deepsomatic) were used to call germline and somatic single-nucleotide variants (SNVs) and small insertions and deletions (InDels), respectively. We used the default options of Clair3 and enabled phasing using WhatsHap^66^. We generated haplotagged BAM files using these phased SNVs and InDels to conduct allele-specific expression analyses with the nf-core RNA-Seq pipeline^67^ and allele-specific methylation analyses with modkit (https://github.com/nanoporetech/modkit). To improve the specificity of somatic SNV and InDel calls, we retained only variants supported by both DeepSomatic and ClairS. In addition, somatic calls were filtered against matched controls by genotyping all variants in the short-read data using FreeBayes (https://github.com/freebayes/freebayes). Variants detected in blood samples were subsequently removed.

#### Structural variant calling

We used Delly^68^ and Severus^69^ to identify structural variants (SVs) from the long-read data. Because of our tumor-only long-read sequencing approach, we used a panel-of-normal (PoN) approach to distinguish germline and somatic SVs. For Severus and Delly, we used the 1000 Genomes ONT data generated by Schloissnig et al.,^70^ for the generation of a PoN. Severus additionally requires phased SNVs, which we computed using Clair3^65^ (see above). We also used the default GRCh38 VNTR bed file provided by Severus for calling SVs. For delly, we used the default options in the long-read (lr) SV calling mode. To compute consensus SVs between Severus and Delly, we used Sansa’s compvcf subcommand (https://github.com/dellytools/sansa) to compare the output VCFs and identify matching somatic SVs between the two tools. We also used the matching short-read germline data for further filtering, where we computed SVs using Delly and mobile element insertions using MELT^71^. Because short-read sequencing has limited sensitivity for insertion detection, we applied an additional stringent filtering step for insertions by implanting them into the GRCh38 reference. Short-read control data were then remapped to this augmented reference, and insertions were flagged as germline artefacts if their median coverage reached at least 50% of the genome-wide median coverage in the controls. To assign SVs to haplotypes, we split the BAM files by haplotype and re-run Delly on the haplotype-specific BAM files.

#### Structural variant annotation

We used SVAN^70^ (https://github.com/REPBIO-LAB/SVAN) to annotate all consensus SV calls for variable number of tandem repeat (VNTR) variations, duplication variants and mobile element insertions (MEIs). We used the default options of SVAN for insertion and deletion annotation with the provided BED files for VNTR and mobile element annotation.

#### Somatic retrotransposition analysis

To enhance specificity of our candidate somatic L1 callset, we called mobile element insertion in the matched short-read germline data using MELT^71^. We then compared all somatic L1 insertion with the germline L1 data using bedtools^72^ intersect on the insertion site with ±250 bp to account for potential breakpoint ambiguities between the short- and long-read data. All candidate somatic L1 insertions overlapping a germline element were subsequently removed. To compare short- and long-read L1 calling, we used the matched tumor-normal B125 bladder cancer data as the tumor was sequenced to very high-coverage (82x). For the short-read data, we used MELT both on the tumor and matched control and compared the somatic L1s with the long-read based L1 calls for this sample. 6 out of 9 somatic L1s were directly confirmed by the short-read analysis. For the remaining 3, we used manual inspection in IGV^73^ to determine the somatic or germline L1 status. In one of the three events, we could not observe any abnormal mappings in the corresponding control, suggesting a somatic event. The other two candidate somatic L1s did show abnormal paired-ends in the control that were not called as an L1 by MELT but could be indicative of an insertion and hence, we labelled these events as unclear. An analogous comparison of the short-read based somatic L1 events called by MELT with long-read data showed an overlap of 6 out of 12 called SVs with only 2 long-read missed events classified as somatic after manual inspection in IGV^73^. One event was likely missed due to coverage (only 2 reads support) and the other event was a very short somatic insertion (81 bp) that was called by Delly^68^ and Severus^69^ but not annotated as an L1 by SVAN.

#### Retrotransposon-linked structural variants

Since Delly^68^ and Severus^69^ do not identify complex structural variants where somatic L1s can co-occur at the breakpoint of other SV types, we developed an algorithm to perform a targeted search for L1 sequence fragments at SV breakpoints. This new method, called BreakTracer (https://github.com/tobiasrausch/breaktracer) first identifies split-reads at SV breakpoints and then searches for L1 sequence fragments between these junctions to subsequently locally assemble all reads that support the same genomic breakpoints connected by an L1 insertion. We further filtered all breakpoints that co-occurred in multiple samples to prevent false positive retrotransposon-linked SV calls from mis-alignments or germline L1s that are not present in the reference genome. To further corroborate the presence of L1 fragments at SV breakpoint junctions, we additionally performed independent local read assemblies for all examples shown in the Supplementary Fig. 13 using Shasta (https://github.com/chanzuckerberg/shasta).

#### Copy-number variant calling

We used CNVkit^74^ and Delly’s cnv^68^ subcommand to call copy-number variants and generate read-depth profiles. For Delly, we used a window size of 25,000 bp with the default GRCh38 mappability map downloaded from the Delly GitHub repository. For CNVkit, we selected the ‘wgs’ method and dropped low-coverage regions with the default GRCh38 reference file at 5k resolution.

#### ecDNA identification

We used CoRAL^75^ to identify ecDNA structures from the long-read data with the copy-number profile computed by CNVkit^74^. We used the following parameter values: seed CN = 4.0, MBS = 2, CDA = 0.01. We further refined the structural predictions using ecDNAInspector^76^, a tool using orthogonal structural variant calls to select high confidence ecDNA predictions.

#### De novo assembly of source and somatic L1

We performed targeted de novo assembly of somatic L1 insertions using the Oxford Nanopore data for B42. Candidate insertion sites were extracted from the mobile-element annotated VCF file and extended by ±5 kb. Overlapping regions were merged, and reads mapping to these windows were extracted from the tumor alignment file using samtools^58^. Structural variants were called with Delly^68^ in long-read (lr) mode with the output of SV-supporting reads enabled, and insertions between 500–9,000 bp were selected. L1 insertions that did not achieve a “PASS” quality score from embedded filters were excluded from downstream analysis. If a single variant was detected in a region, supporting reads were assembled using Shasta^77^. The resulting contigs were used to realign the original reads to the assembly, as well as a canonical L1 sequence from MELT^70^ using minimap2^61^ to confirm the L1 presence in the assembly. From these alignments, we inferred the L1 location in the contig using Alfred^63^ and its methylation status using modkit. L1 insertion metrics with length, strand, coverage, and insertion support information are provided in Supplementary Data 12. We compared our source L1s against the published reference of repeat element L1s (UCSC Genome Browser hg38 RepeatMasker - LINE-1 track) and two additional source L1 lists^51,54^ to identify novel source L1s in our cohort. To identify “hot” L1 source regions, we merged overlapping L1 source elements and took the smallest start base pair and largest end base pair to define an L1 source region. These were checked against the previously cited reported L1 source element lists for overlap.

#### Analysis of L1 regulation via methylation

To assess the methylation status of somatic L1 insertions in patient B42, we used modkit pileup to extract CpG methylation calls from ONT reads aligned to each assembled insertion. We then processed the resulting pileup files in R. After filtering for sites with coverage >5, we merged the methylation data with the corresponding L1 insertion metadata, including orientation and genomic coordinates. For full-length insertions (>6,000 bp), we defined the L1 promoter as the first 500 bp (strand-aware), and the L1 body as the remainder of the element. We then assigned each CpG to either promoter, body or 500 bp flanking region and compared methylation levels between the regions using the two-sided Mann–Whitney U test. We next combined coordinate data from assembled L1 fragments (not full-length) and filtered out low-quality entries. L1 lengths were computed based on read alignment positions of a canonical L1 element (see above). Promoter regions were defined as the first 500 bp of each insertion, adjusted according to strand orientation. This annotated dataset provided the basis for integrating methylation profiles with insertion features.

#### Circos Plot

L1 promoter and body regions were annotated using a curated Excel file and converted to BED format. Methylation data from nanopore sequencing pileup files were intersected with these regions using bedtools to extract CpG methylation calls. Average methylation fractions were calculated per region and linked to L1 insertion partner data. Copy number variation was assessed from tumor coverage files and integrated with methylation data for downstream analyses. The plot was created using the circlize R package.

#### L1 expression analysis

To estimate locus-specific L1 expression, we used L1EM^44^ with default parameters and the GRCh38 reference genome. Locus-specific expression estimates were then summarized into a global (mean) L1HS transcription metric by summing (and averaging) the individual L1HS FPM values, restricting the analysis to properly initiated transcripts originating at the 5′ UTR.

#### Integrative analysis of methylation, SNVs and RNA-seq expression

Promoter regions for all genes in the hg38 reference genome were extracted using the GRanges R package and defined as 2000 bp upstream and 400 bp downstream of the transcription start site (TSS). Only unique promoters mapping to canonical chromosomal regions were retained for downstream analysis. Differential methylation between haplotypes was assessed for each tumor sample using *modkit dmr pair*. Statistical significance for each promoter region was evaluated using a binomial test, followed by Benjamini–Hochberg correction for multiple testing. Promoters were considered significantly differentially methylated if they met the following criteria: q < 0.05, modkit score > 50, and absolute haplotype methylation effect size > 0.2.

Significantly differentially methylated promoters were subsequently overlapped with single-nucleotide variant (SNV) calls and matched with corresponding normalized RNA-seq gene expression values. In addition, genes harboring haplotype-specific differentially methylated promoters were annotated with COSMIC cancer gene status (v103) obtained from the COSMIC database.

#### Detection of somatic L1 insertions from cfDNA

Using somatic L1 insertions and their surrounding genomic sequences assembled from the ONT data, personalized genome references were constructed for each patient. Patient-specific cfDNA reads were then aligned to these customized references. To maintain high specificity, we only focused on high-quality alignments covering the L1 junctions. All candidate alignments were manually inspected in IGV. An insertion was considered as detected if it was supported by at least one high-quality, junction-spanning read.

#### Short-read analysis of benign bladder tissue samples and tumor samples used for direct comparison between short and long-read sequencing

For the short-read sequencing data we used MELT^71^ for mobile element discovery, Delly^68^ for structural variant discovery and read-depth profiles, the nf-core/sarek pipeline^78^ for Mutect2 SNV and Indel calls and nf-core/oncoanalyser^79^ for Sage SNV and Indel calls. Consensus short-read SNV calls were computed using the bcftools^80^ isec command.

#### L1 enrichment analysis at copy-number variant breakpoints using PCAWG data

Using the short-read PCAWG^40^ data, we classified each tumor-specific CNV breakpoint as either SV-explained or SV-unexplained based on a 5 kbp breakpoint overlap window. We then evaluated the overlap of somatic L1 insertions with CNV breakpoints in each category (CNVs with and without associated SVs). We observed a significant enrichment (p < 0.001) of somatic L1 insertions near SV-unexplained CNV breakpoints across tumor histologies in PCAWG with at least five samples containing a minimum of one somatic L1 near a CNV breakpoint. This pattern suggests the existence of a previously hidden landscape of complex somatic SVs involving L1 sequence fragments that cannot be fully resolved by short-read sequencing.

#### L1 insertion on ecDNA structure analysis

To identify whether L1 insertions were present on ecDNA rather than linear chromosomes, we used our haplotype-resolved long-read sequencing data and counted the total reads at each insertion position that supported the insertion versus no insertion. We reasoned that the highly amplified haplotype reads at each position would represent the ecDNA reads; thus, if an insertion was present on an ecDNA, there should be a high ratio of reads supporting the insertion versus not. Otherwise, if the ratio of insertion:no insertion reads was small, this would indicate that there was no insertion on the highly amplified ecDNA.

#### Visium spatial gene expression for FFPE tissue

Preparation of FFPE tumor tissue was done according to the 10X Tissue Preparation Guide. RNA quality assessment, section collection and placement, tissue adhesion test, probe hybridization and ligation, probe release and extension, library construction, and sequencing were done as described in our previous work^81^.

#### Visium data processing and quality control

Raw visium FASTQ files were processed using the 10x Genomics SpaceRanger (version 2.0.1) with the GRCh38 reference genome. Downstream analyses were carried out using the Scanpy package (version 1.11.0)^82^. Spots with low number of counts (cutoff selected based on histograms) were removed. Genes detected in fewer than 10 spots were filtered out.

#### Copy number inference from Visium spots

Copy number inference was performed using CopyKAT (version 1.1.0)^83^, with the following parameters: id.type = “S”, ngene.chr=5, win.size=25, KS.cut=0.1, distance = “euclidean”, output.seg = “FALSE”, plot.genes = “TRUE”, genome = “hg20”. Spots annotated as non-tumor by a pathologist were used as a reference for diploid cells via the norm.cell.names parameter. For samples where confident extraction of non-tumor spots was not feasible, the top 100 spots with the highest ESTIMATE score^84^ were used instead. CopyKAT-inferred copy number profiles were subsequently mapped to fixed 25 kb genomic windows for downstream analyses.

To define tumor subclones, we applied Leiden clustering to CNV profiles from all spots classified as aneuploid by CopyKAT. To determine the optimal Leiden resolution, we evaluated resolutions ranging from 0.1 to 0.4 and selected the one yielding the highest Calinski–Harabasz index. If the clustering solution consistently returned a single stable cluster across multiple resolutions, this was chosen as the final result.

Given that individual Visium spots may contain a mixture of tumor and non-tumor cells, the tumor-specific CNV signal can be diluted, potentially resulting in false subclone identification. To mitigate this, we computed a pairwise Pearson correlation coefficient between the pseudobulk CNV profiles of each subclone. Subclones with correlation coefficients above 0.95 were merged. Finally, for every spot we calculated CNV score defined as:

1 $${CNV}\,{score}\,=\,\frac{1}{n}{\sum }_{i=1}^{n}|{{CN}}_{i}|$$

where $n$ is the number of all genomic bins and ${{CN}}_{i}$ is the log ratio from CopyKAT at the $i$-th bin.

#### Cell type deconvolution from Visium

Cell2location^85^ was used for cell type deconvolution. To compute the reference cell type signatures, the dataset by Gouin III et al., was used^86^ (GSE169379). As an alternative reference to validate the deconvolution, the dataset by Chen et al., was used^87^ (PRJNA662018). For the spatial mapping, we set hyperparameters to ‘N_cells_per_location = 20‘ and ‘detection_alpha = 20‘. Relative cell type abundance per spot was used for all the analyses.

#### Identification of ecDNA-enriched regions in Visium data

To identify the ecDNA enrichment in spatial transcriptomics data, we leveraged sample-matched ecDNA structures reconstructed from ONT. For each sample, we extracted all genes located on the reconstructed ecDNA circles and retained only those detectable in the corresponding Visium gene expression matrix. These genes were used to define sample-specific ecDNA transcriptional signatures, where each signature was derived and applied within the same tumor. Next, we performed an additional filtering step to these gene sets. Specifically, we retained only genes whose mean expression across aneuploid Visium spots was highest in the corresponding sample compared to the remaining samples in the cohort. Using these curated gene sets, we then computed signature enrichment scores using the ‘run_aucell‘ function from the decoupler-py package (version 1.9.2)^88^ for each Visium spot. To account for differences in tumor purity, we regressed enrichment scores on the CNV score, which was used as a proxy for tumor purity.

Finally, ecDNA enrichment was aggregated at the level of transcriptionally similar spatial clusters identified by SpatialDE2^89^, considering only aneuploid spots within each cluster. Spatial clusters containing three or fewer spots were excluded from downstream analyses. Comparisons between ecDNA-enriched clusters, as well as between ecDNA-enriched and ecDNA-depleted clusters, were performed using clusters with comparable cluster-level CNV scores to ensure similar tumor purity. Differential gene expression between clusters was performed in Scanpy with the function rank_genes_groups, using the two-sided Mann–Whitney U test, followed by gene set enrichment analysis (GSEA).

#### Bulk RNA data analysis

For RNA-Seq quality control and gene quantification we used the nf-core RNA-Seq pipeline^67^. Reads were trimmed using TrimGalore (https://github.com/FelixKrueger/TrimGalore) and aligned using STAR^90^ version 2.7.11 to the GRCh38 reference genome. Salmon^91^ version 1.10 was used for computing gene counts and normalized transcripts per million (TPM) values. DESeq2^92^ was used for differential gene expression analyses of ecDNA positive samples compared to ecDNA negative samples and L1 positive samples compared to L1 negative samples. Pathway enrichment using cancer hallmark genes sets employed Gene Set Enrichment Analysis (GSEA). The top three L1-count samples were labeled as “L1-high”, and the bottom three L1-count samples were labeled as “L1-low”. The merged Salmon gene counts were then used to produce a ranked gene list for L1-high versus L1-low samples with DESeq2^92^ (v1.38.3). This ranked gene list was provided to GSEA (v4.4.0) for a GSEAPreranked run on Hallmark gene sets (h1.all.v2025.1.Hs.symbols.gmt).

#### Gene fusions

For gene fusion identification, we used the nf-core RNA fusion pipeline^93^ and manually cross-checked predicted RNA fusions with the consensus somatic SV calls from the long-read DNA sequencing data. For the gene fusions supported by both RNA and DNA, we provide information on which fusion callers predicted the fusions and if there was a consensus. Gene fusion visualizations were generated using IGV and Arriba^94^.

#### Identification of the driver genes in the patient cohort

We first performed a literature search to compile a list of established bladder cancer driver and marker genes. These genes were then screened in our patient cohort for copy number alterations (deletions, amplifications and loss of heterozygosity). All genes were visualized using the ComplexHeatmap R package.

#### Clonality of structural variants and mutational signatures

Whole-genome sequencing data from clonal and subclonal single-nucleotide variants (SNVs) were analyzed to characterize mutational signatures in bladder cancer samples. SNV calls were parsed into sample-specific GenomicRanges objects using the GenomicRanges^95^ R package with genome built GRCh38 (hg38) as reference. Structural variant clonality was determined by calculating the variant allele frequency (VAF) for each SV, with a VAF > 0.3 indicating a clonal rearrangement and VAF ≤ 0.3 a subclonal rearrangement. Mutation type occurrences and 96-trinucleotide context mutation matrices were generated using the MutationalPatterns^96^ R package, using *fit_to_signatures_strict* function. Known COSMIC SBS mutational signatures (v3.2) were obtained and fitted to the mutation matrices to estimate signature contributions per sample via non-negative least squares regression implemented in MutationalPatterns.

Signature contributions were normalized to relative values within each tumor sample. Tumors with cosine similarity >0.95 between observed and reconstructed mutation profiles were selected for further analysis. Clonal-to-subclonal ratios of signature contributions were calculated per tumor, and aggregated ratios were visualized.

#### Analysis of the L1 and TP53 status in PCAWG cohort

PCAWG samples were classified as “amplicon” or “no amplicon” based on CNV annotations (CN > 6). L1 insertion counts were compared between amplicon and no-amplicon groups, stratified by *TP53* mutation status (mutant vs wild-type). Counts were log10-transformed for normalization.

#### Fluorescence in situ hybridization (*E2F3*)

Two-colour interphase FISH was performed on human tissue sections using a rhodamine-labelled probe (centromere 6) and a FITC-labelled probe (*E2F3*; clone RP1-177P22). The probes were indirectly labelled via Nick translation. Pre-treatment of slides included use of Sodium thiocyanate (1 M NaSCN) at 80 °C. Digestion was performed with pepsin solution (1 mg/ml) for 30 min. Probes were applied on the slide, followed by a denaturation step for 10 min at 75 °C. Due to indirect probe labelling, signal detection was done on the next day, after washing slides with 50% formamide, followed by 0.5x SSC buffer. Samples showing sufficient FISH efficiency (>90% nuclei with signals) were evaluated.

#### Immunostaining of LINE-1 ORF1p and E2F3

Formalin-fixed paraffin-embedded tissue sections (4 μm thickness) were deparaffinized in xylene and subsequently hydrated. Antigen retrieval was carried out by the incubation of sections in a 10 mM citrate buffer adjusted to pH 6.0 at 95–100 °C in a steam cooker for 40 minutes. The sections were blocked with 10% donkey serum diluted in 1x PBS. For single staining, Anti-LINE-1 ORF1p antibody raised in mouse (Sigma, # MABC1152, clone 4H1) was diluted 1:100 and kept for overnight incubation. Secondary donkey anti-mouse IgG Alexa Fluor 488 antibody (Invitrogen, #A-21202) was used at a dilution of 1:500. Anti-E2F3 antibody raised in rabbit (Invitrogen, PA5-106407) was diluted 1:50 and kept for overnight incubation. Secondary anti-rabbit Alexa Fluor Plus 594 (Invitrogen, A32754) was used at a dilution of 1:300. Mounting of slides was performed with DAPI Fluoromount-G mounting medium (Southern Biotech, #0100-20). For double staining, Anti-LINE-1 ORF1p antibody (dilution 1:100) was incubated simultaneously with E2F3 antibody (dilution 1:50) overnight. Antibody dilution was done with the Antibody diluent from Agilent (S3022). Secondary donkey anti-mouse IgG Alexa Fluor 488 antibody (Invitrogen, # A-21202) was used at a dilution of 1:500 and the secondary anti-rabbit Alexa Fluor Plus 594 (Invitrogen, A32754) at a dilution of 1:300. Mounting of slides was performed with DAPI Fluoromount-G mounting medium (Southern Biotech, #0100-20).

#### Confocal microscopy

Leica SP8 confocal microscope was used for the imaging of sections of FFPE tissue stained for LINE-1 ORF1p. Imaging was performed using the Leica LAS X software. Z-stacks were imaged with a step size of 0.5 μm. The start and end points of the Z-stack were set manually. Maximal intensity projections were made with Fiji with the Z projection function where the projection type was set to maximal intensity where all the stacks imaged were included in the projection.

#### Imaging and scanning of human tissue sections

All H&E, E2F3 and LINE-1 ORF1p stained sections were imaged using the Zeiss Axioscan 7 at the light microscopy facility at the DKFZ. Sections were imaged at 20x magnification with settings adapted from the fluorescence and brightfield profile for the respective fluorophores. Sample detection was performed by manual selection and focal points were assigned manually for each section.

#### Fluorescence in situ hybridization (*MDM2*)

Dual color interphase FISH was performed on patients’ FFPE bladder tissue sections (5 µm) using the XL-*MDM2* enumeration probe (D-5047-100-OG, Metasystems, Germany).

After deparaffinization and rehydration, slides were pretreated with sodium thiocyanate (1 M NaSCN) at 80 °C. Digestion was performed with a pepsin solution (1 mg/ml in 0.9% NaCl) for 30 min in a water bath at 37 °C. Slides were incubated with the probe for 10 min at 75 °C for co-denaturation and placed in a humidified chamber at 37 °C overnight for hybridization.

After stringency wash (0.4% SSC at 37 °C, 2× SSC–0.05% Tween at RT), nuclei were counterstained with DAPI and mounted (VECTASHIELD Vibrance® Antifade Mounting Medium with DAPI).

Regions of interest were scanned at 40× and 100× oil magnification (Olympus Scanner VS200). Images acquired at 100× magnification were used for subsequent automated FISH signal analysis.

#### Automated quantification of *MDM2* FISH signals in space

For each 100× field, nuclei were segmented from the DAPI channel using Cellpose^97^ (cyto model; diameter = 100 px; flow threshold = 0.3; cell probability threshold = 0.2), and all downstream measurements were restricted to the nuclear area. To reduce boundary effects, nuclear masks were eroded by one pixel using a distance-transform–based shrink step before quantification. The *MDM2* FISH signal (Cy3/561 channel) was quantified on a per-nucleus basis using histogram-based Triangle thresholding^98^: for each nucleus, an intensity threshold was computed from the within-nucleus Cy3 distribution, and pixels above this threshold were classified as foreground signal. For every nucleus, we measured the foreground area fraction (fraction of nuclear pixels above the Triangle threshold) as an intensity-independent quantity to directly compare B4 and B96 despite their different overall intensity values (due to different imaging parameters). Nuclei below a global minimum area cutoff (defined as the smallest 1% of nuclei across the dataset; 2,871 px) were excluded to remove small debris and segmentation artefacts. Because the foreground area fractions are bounded between 0 and 1, we applied a logit transformation *z* = log(*x*/(1-*x*)) prior to Gaussian mixture modeling to stabilize variance and remove boundary effects. We first fitted two Gaussian components (ecDNA-negative and tail) to the pooled B96 nuclei. The means and standard deviations of these components were fixed. A third Gaussian component representing ecDNA-positive nuclei was fitted to pooled B4 nuclei. Finally, with all three component parameters fixed, we estimated their mixture proportions separately for each region, yielding region-specific ecDNA-positive, ecDNA-negative, and tail fractions. To assess the stability of mixture proportion estimates, we performed bootstrap resampling (100 iterations), each time refitting the model using a random 50% subsample of nuclei per region. Region-specific fractions are reported as mean ± standard deviation across bootstrap iterations. Nuclei assigned to the tail component (typically *x* > 0.25) exhibited low cumulative nuclear Cy3 intensities relative to nuclei with lower foreground fractions, consistent with thresholding artefacts rather than true ecDNA amplification. In addition, we assessed intra-sample heterogeneity in B4 by jointly analyzing cumulative nuclear Cy3 intensity and the nuclear coefficient of variation.

#### Identification of putative enhancer hijacking events using Pyjacker

Pyjacker is a computational method developed for the systematic prediction of enhancer hijacking events using various types of genomic data^36^. Pyjacker requires the following input files: a merged table of sample-specific structural variant breakpoints, corresponding gene expression values in TPM (Transcripts Per Million), and reference annotation files, including gene annotations and chromosome coordinates. Additionally, some optional inputs can be specified. In this study, the breakpoints table was generated from ONT (Oxford Nanopore Technologies) sequencing data and gene expression data were generated through RNA sequencing for the same samples. Furthermore, ChIP-seq datasets (ENCFF515VMS, ENCFF439WSM, ENCFF487EPL) were obtained from the ENCODE database^99^ to run the ROSE (Rank Ordering of Super-Enhancers) pipeline (https://bitbucket.org/young_computation/rose/src/master/) for identifying putative enhancers. Topologically Associated Domains (TADs) from Hi-C data were obtained from ENCODE (ENCFF682EJU) to complement the analysis. Pyjacker’s output is a table listing gene names, their genomic coordinates, and the corresponding false discovery rates (FDRs) for genes putatively affected by enhancer hijacking. To visualize the results, RStudio version 4.4.2 and figeno^36^ were used.

#### Neoepitope prediction from somatic SNVs and InDels

Somatic single nucleotide variants (SNVs) and small insertions/deletions (InDels) called by Clair3 and ClairS were annotated with Ensembl VEP (v108, GRCh38). HLA class I genotypes were determined by OptiType^100^ (https://github.com/FRED-2/OptiType) from short-read germline DNA. Neoepitope predictions for HLA class I were performed for peptides of 8-15 amino acids in length using pVACseq^100^ (pVACtools 5.3.0, https://github.com/griffithlab/pVACtools). Predicted neoepitopes from pVACtools^101^ were manually filtered based on affinity thresholds (median IC50 < 500 nM and elution rank <2%; predictors: NetMHCpan, BigMHC, MHCflurry, MHCnuggets) and variant quality (tumor DNA VAF > 25% and reads >10). Patients were grouped by L1 insertion burden, with >15 insertions labeled as high (*n* = 5) and <5 as low L1 insertion count (*n* = 13). The number of predicted neoepitopes was compared between the L1 groups using a two-sided Mann-Whitney U test.

#### Data visualization and statistics

For all data visualizations and statistics we used the following packages in R: dplyr, ggplot2, tibble, tidyr, stats, DESeq2, scales, DNAcopy, reshape2, grid, gridExtra, cowplot, ComplexHeatmap. We further used the following packages in Python: gzip, pandas, matplotlib, numpy, glob, gseapy, scikit-learn, scipy, seaborn, pysam, scanpy, scikit-image. Exact package versions can be found in the respective GitHub pages listed in the Code Availability section.

#### Statistics & reproducibility

No statistical method was used to predetermine sample size. No data were excluded from the analyses, except for two Visium samples that did not pass QC. The experiments were not randomized. The investigators were not blinded to allocation during experiments and outcome assessment.

### Reporting summary

Further information on research design is available in the Nature Portfolio Reporting Summary linked to this article.

## Supplementary information

Supplementary Information

Description of Additional Supplementary Files

Supplementary Data

Reporting Summary

Transparent Peer Review file

## Source data

Source Data

## Data Availability

The data generated in this study are available via controlled access in the German Human Genome-Phenome Archive (GHGA, data.ghga.de under the GHGA Accession GHGAS23497594176126). Further details, including the data access policy for the study, can be found there. Source data for spatial transcriptomics, cfDNA and FISH analysis can be found here: (10.5281/zenodo.20085382). Source data are provided with this paper. Due to the sensitive nature of human genomic data and to protect participant privacy, the data is access-controlled. Data access is governed by the Data Access Committee (DAC), which ensures compliance with ethical, legal, and institutional guidelines. Researchers seeking access must submit a data access request via the EGA platform, outlining the intended use of the data. Access will be granted to qualified researchers affiliated with academic or research institutions, and requests will typically be reviewed and responded to within 30 working days. Applicants will be required to provide a brief research proposal and, where applicable, documentation of ethical approval and a signed data sharing agreement. Requests will be evaluated on a case-by-case basis to ensure compliance with participant consent, ethical approvals, and data protection requirements. Data will be made available only for research purposes consistent with the original study objectives. Once approved, data will be available to the requestor until project completion. The remaining data are available within the Article, Supplementary Information or Source Data file. Source data are provided with this paper.

## Code availability

All open-source software code developed in this study is available via GitHub. The spatial, cfDNA and FISH analysis scripts are available at (https://github.com/OtonicarJan/bladder-spatial), (10.5281/zenodo.20085382) and (https://github.com/RippeLab/ecdna-fish-bladder-cancer). The long-read genomic data, expression and gene set enrichment analysis scripts are available at (https://github.com/OsredekIvana/Bladder_cancer, https://github.com/sophiepribus/dkfz-embl-bladder) and (https://github.com/tobiasrausch/blca) (10.5281/zenodo.20122297). The method to screen long-read alignments for retrotransposon-linked SVs is available at (https://github.com/tobiasrausch/breaktracer) (https://zenodo.org/records/20040221). These repositories also contain files needed to reproduce the analysis in the scripts.
