# Supplementary material for: Multiomic profiling links L1 retrotransposition to genomic instability and ecDNA in bladder cancer
Source: Nat Commun. 2026 Jul 16;17:6384. doi: 10.1038/s41467-026-75399-6 (PMC13376730; doi:10.1038/s41467-026-75399-6)
Supplement: Supplementary file 1 — Supplementary Information [file 41467_2026_75399_MOESM1_ESM.pdf]

Supplementary Information

a B42: FGFR3-TACC3

b B5: ZYG11B-RAF1

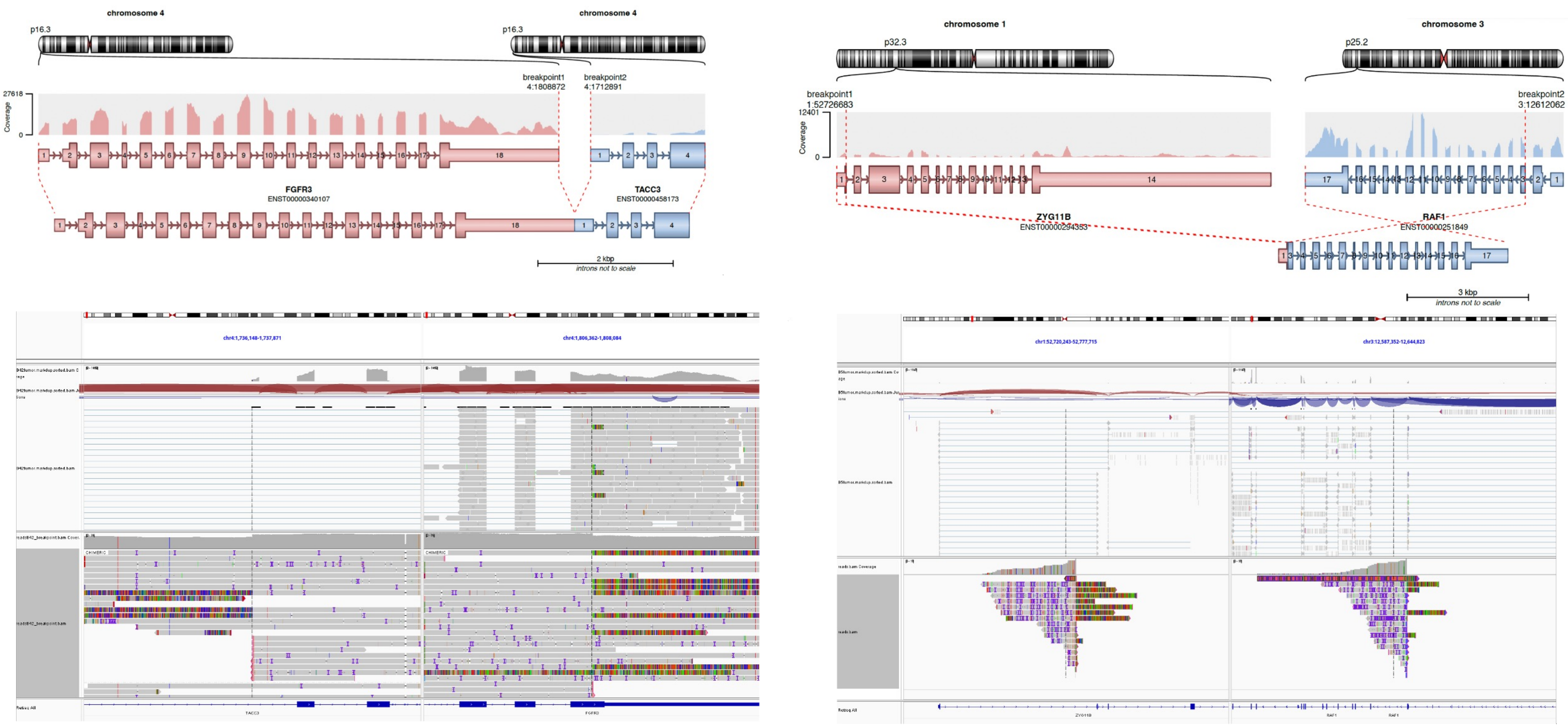

Supplementary Figure 1. Arriba visualization of two highly expressed gene fusions with oncogenic potential. a) FGFR3-TACC3 and **b)** ZYG11B-RAF1 in patients B42 and B5, respectively (visualised using Arriba and IGV).

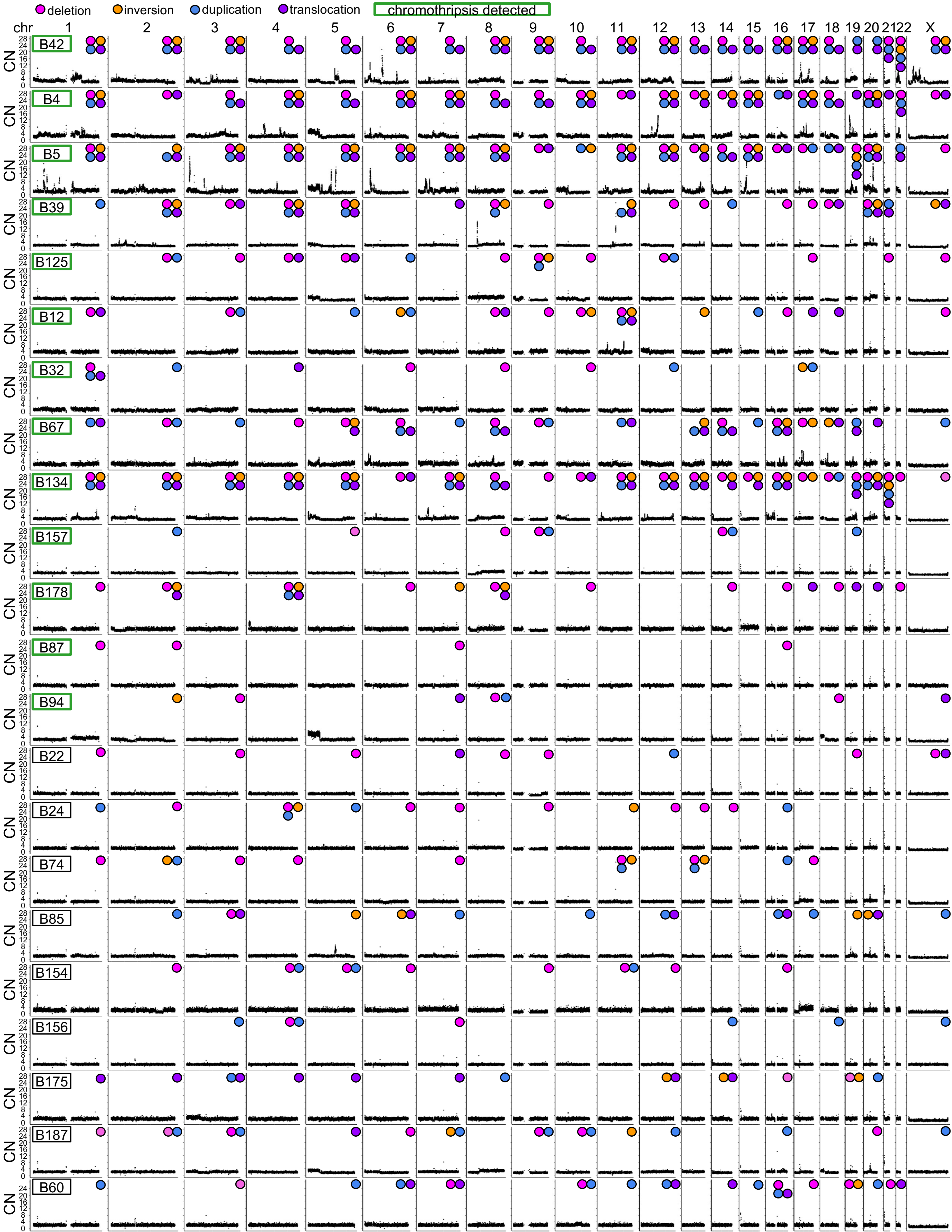
deletion
inversion
duplication
translocation
chromothripsis detected
chr
1 2 3 4 5 6 7 8 9 10 11 12 13 14 15 16 17 18 19 20 21 22 X
CN
B42
B4
B5
B39
B125
B12
B32
B67
B134
B157
B178
B87
B94
B22
B24
B74
B85
B154
B156
B175
B187
B60

Supplementary Figure 2. Long-read WGS of bladder cancer samples reveals broad copy-number variation, sporadic high copy-number amplifications, and extensive structural variants. Chromosomes are labeled with the detected structural variation events. Samples scored positively for chromothripsis are labeled in green.

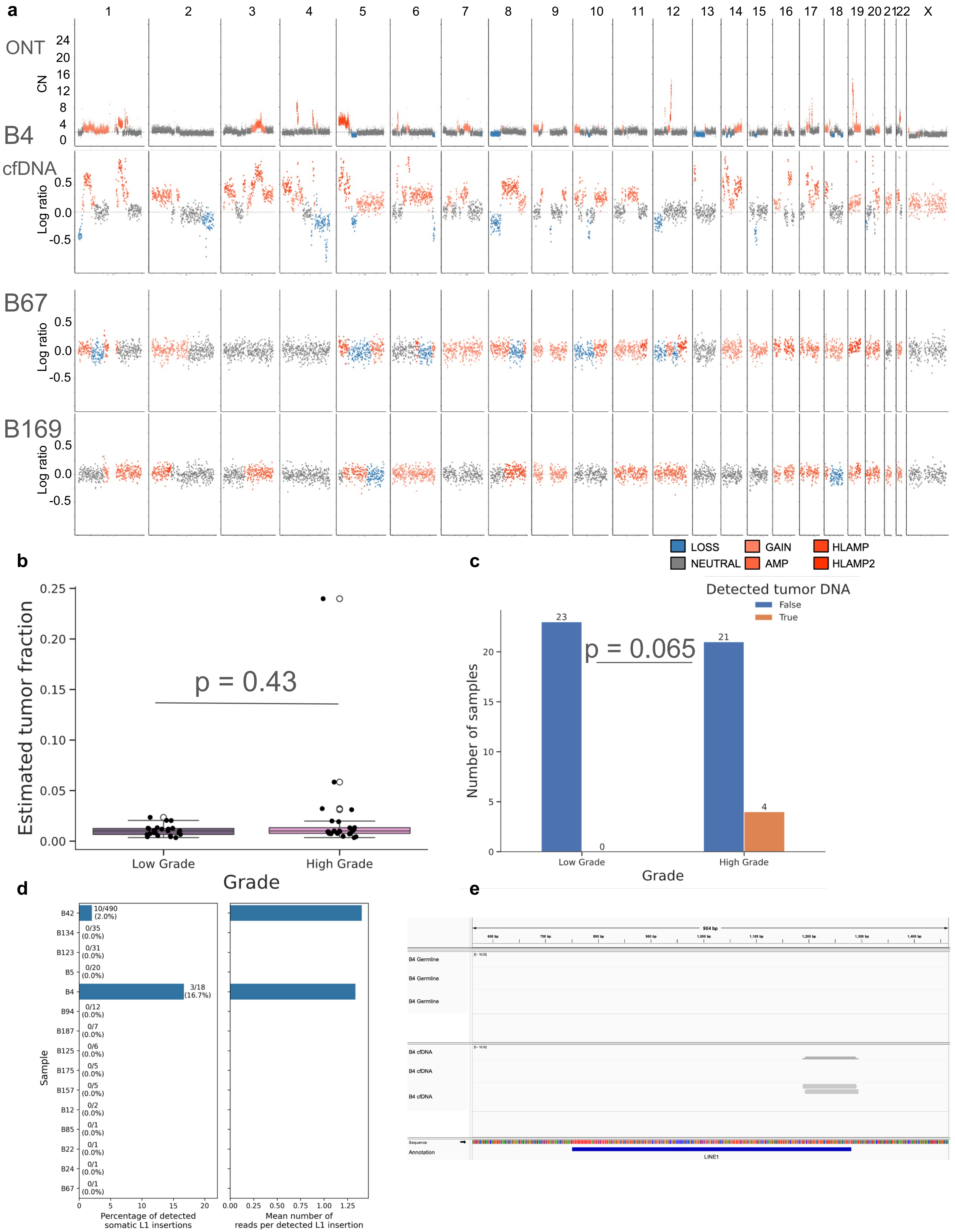
a
1 2 3 4 5 6 7 8 9 10 11 12 13 14 15 16 17 18 19 20 21 22 X
ONT
CN
24 20 16 12 8 4 0
B4
cfDNA
Log ratio
0.5 0.0 -0.5
B67
Log ratio
0.5 0.0 -0.5
B169
Log ratio
0.5 0.0 -0.5
LOSS
NEUTRAL
GAIN
AMP
HLAMP
HLAMP2
b
Estimated tumor fraction
0.25 0.20 0.15 0.10 0.05 0.00
p = 0.43
Low Grade
High Grade
Grade
c
Detected tumor DNA
False
True
Number of samples
20 15 10 5 0
23
0
p = 0.065
21
4
Low Grade
High Grade
Grade
d
Sample
B42 10/490 (2.0%)
B134 0/35 (0.0%)
B123 0/31 (0.0%)
B5 0/20 (0.0%)
B4 3/18 (16.7%)
B94 0/12 (0.0%)
B187 0/7 (0.0%)
B125 0/6 (0.0%)
B175 0/5 (0.0%)
B157 0/5 (0.0%)
B12 0/2 (0.0%)
B85 0/1 (0.0%)
B22 0/1 (0.0%)
B24 0/1 (0.0%)
B67 0/1 (0.0%)
0 5 10 15 20
Percentage of detected somatic L1 insertions
0.00 0.25 0.50 0.75 1.00 1.25
Mean number of reads per detected L1 insertion
e
904 bp
B4 Germline
B4 Germline
B4 Germline
B4 cfDNA
B4 cfDNA
B4 cfDNA
Sequence
Annotation
LINE1

Supplementary Figure 3. Detection of copy-number alterations and L1 insertions from cfDNA. a) Copy-number alterations for the sample B4 inferred from ONT or cfDNA, B67 (cfDNA only) and B169 (cfDNA only). CN states for ONT: LOSS: CN < 1.5, NEUTRAL: 1.5 <= CN < 2.5, GAIN: 2.5 <= CN < 3.5, AMP: 3.5 <= CN < 4.5, HLAMP: 4.5 <= CN < 5.5, HLAMP2: CN >=5.5. CN states for cfDNA: LOSS: CN < 2, NEUTRAL: CN = 2, GAIN: CN = 3, AMP: CN = 4, HLAMP: CN = 5, HLAMP2: CN >= 6. **b)** Estimated tumor fraction from cfDNA for all 48 bladder cancer patients (low grade, n=23; high grade, n=25). Two-sided Mann-Whitney-U test was used for statistical testing. Rank-biserial correlation (effect size) = 0.134. **c)** Number of samples with detected tumor DNA from liquid biopsy. A threshold of 3% estimated tumor fraction was used to classify detected tumor DNA. One-sided Fisher's exact test was used for statistical testing. **d)** Overview of somatic L1 insertions detection in cfDNA. The left panel shows the percentage of somatic L1 insertions that were detected in cfDNA in each sample (number of detected somatic L1 insertions / all somatic L1 insertions). The right panel represents the mean number of reads supporting each detected somatic L1 insertion. Only samples that are L1-positive based on the long-read sequencing are shown. **e)** An example of a detected somatic L1 insertion in cfDNA for the sample B4 visualized in IGV. The upper track shows the B4 germline sample (DNA from white blood cells), while the bottom track shows the B4 cfDNA sample.

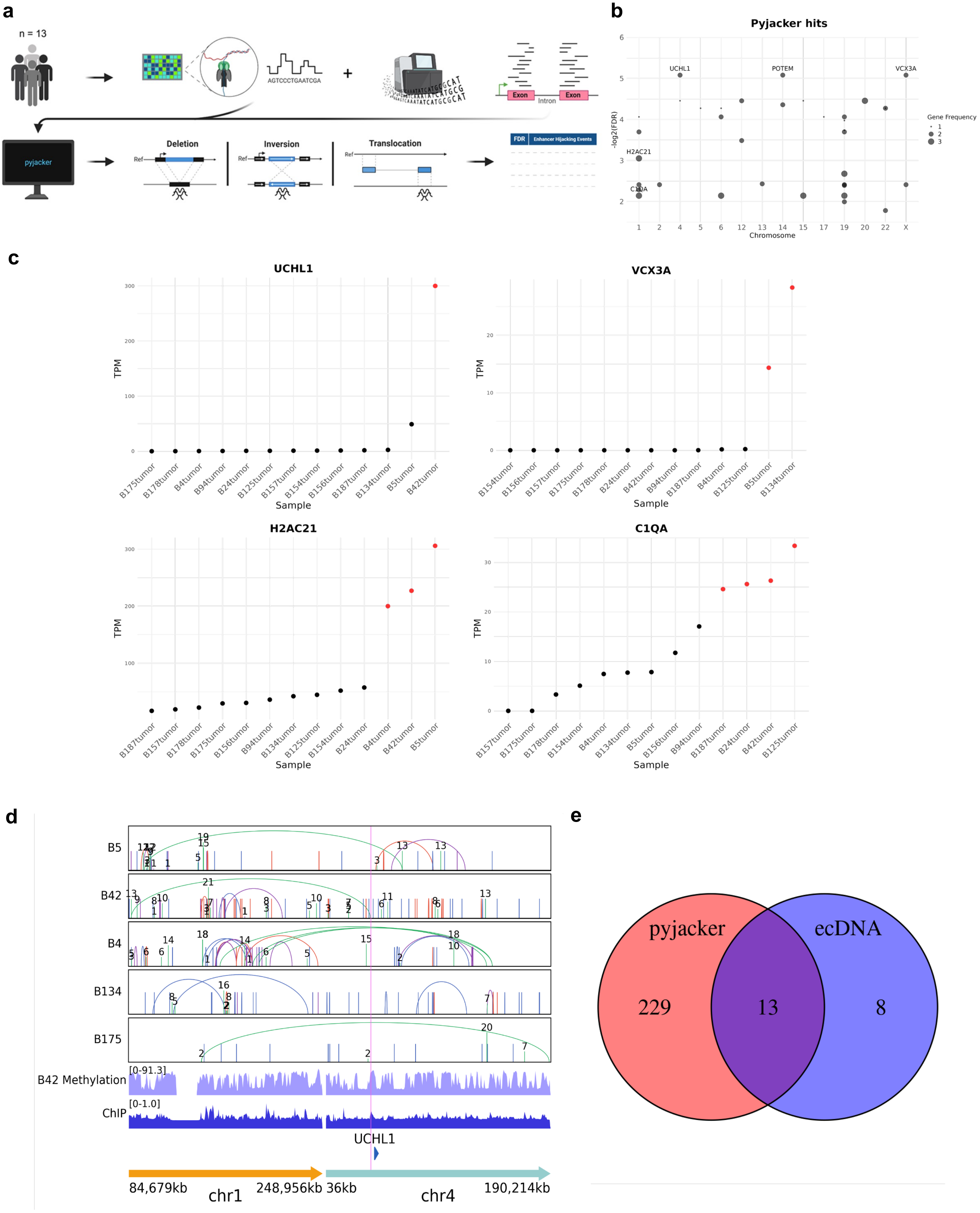

a
n = 13
AGTCCCTGAATCGA
Exon
Intron
Exon
pyjacker
Deletion
Inversion
Translocation
Ref
FDR
Enhancer Hijacking Events
b
Pyjacker hits
UCHL1
POTEM
VCX3A
H2AC21
C1QA
-log2(FDR)
Chromosome
Gene Frequency
c
UCHL1
VCX3A
H2AC21
C1QA
TPM
Sample
d
B5
B42
B4
B134
B175
B42 Methylation
[0-91.3]
ChIP
[0-1.0]
UCHL1
84,679kb
chr1
248,956kb
36kb
chr4
190,214kb
e
pyjacker
ecDNA
229
13
8

Supplementary Figure 4. a) Workflow for the detection of putative enhancer hijacking events (created with BioRender). **b)** Plot displaying the genes associated with the lowest FDR and most recurrent (defined by the number of samples in which the enhancer hijacking was detected) putative enhancer hijacking events as defined by pyjacker. **c)** Representation of gene expression in TPMs for four events with the lowest FDR and highest recurrence, with red points indicating the samples in which the gene is overexpressed (mean + 1 SD). **d)** Visualization of structural variations (SVs) in the *UCHL1* gene across different samples. The SV involved in the putative enhancer hijacking event was a translocation in sample B42, shown here in green. Breakpoints are highlighted in pink. **e)** Venn diagram showing the overlap between genes overexpressed through enhancer hijacking as detected by pyjacker and through ecDNA, respectively.

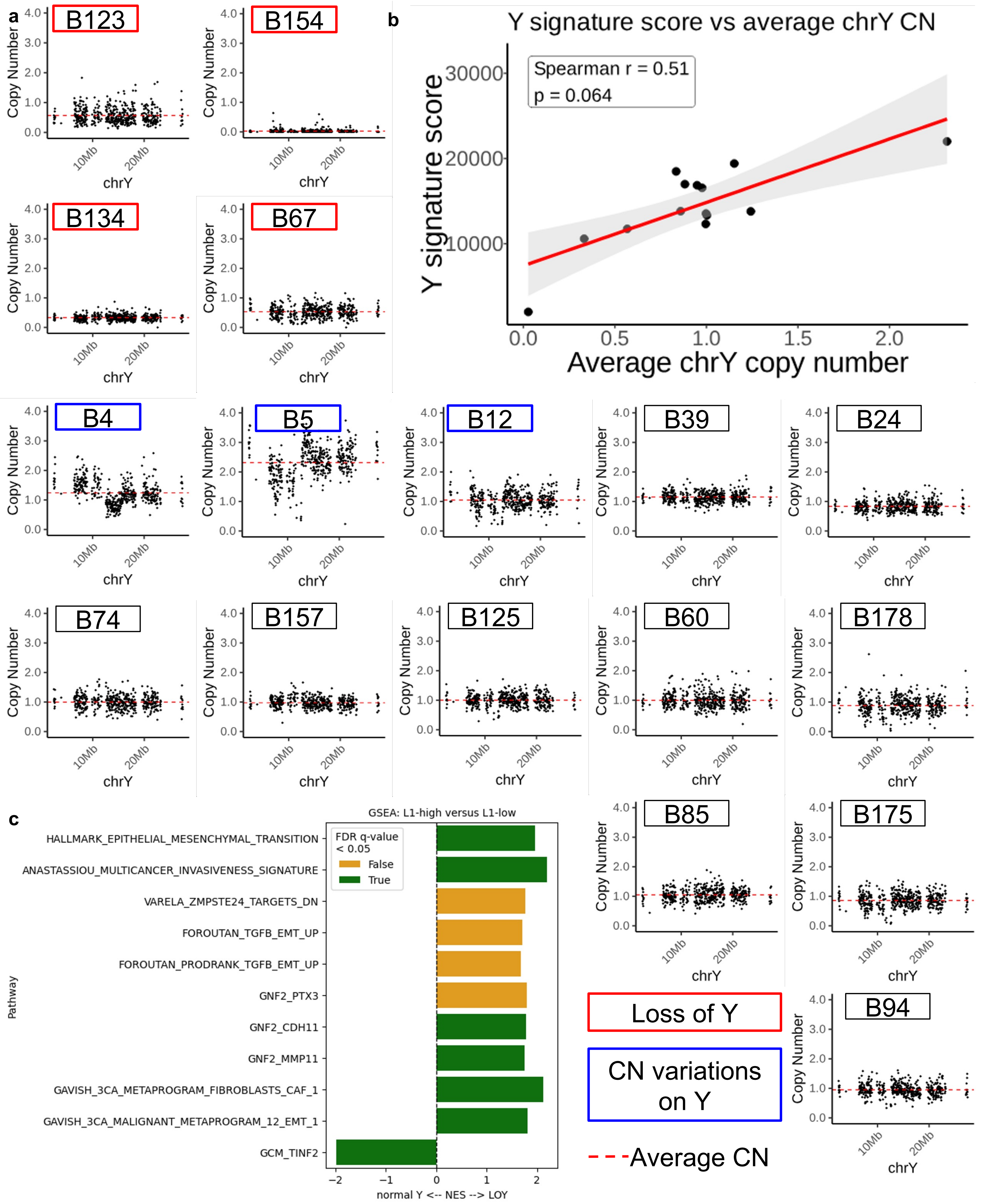
a
B123
B154
B134
B67
B4
B5
B12
B39
B24
B74
B157
B125
B60
B178
B85
B175
B94
Copy Number
chrY
10Mb
20Mb
b
Y signature score vs average chrY CN
Spearman r = 0.51
p = 0.064
Y signature score
Average chrY copy number
c
GSEA: L1-high versus L1-low
FDR q-value < 0.05
False
True
HALLMARK_EPITHELIAL_MESENCHYMAL_TRANSITION
ANASTASSIOU_MULTICANCER_INVASIVENESS_SIGNATURE
VARELA_ZMPSTE24_TARGETS_DN
FOROUTAN_TGFB_EMT_UP
FOROUTAN_PRODRANK_TGFB_EMT_UP
GNF2_PTX3
GNF2_CDH11
GNF2_MMP11
GAVISH_3CA_METAPROGRAM_FIBROBLASTS_CAF_1
GAVISH_3CA_MALIGNANT_METAPROGRAM_12_EMT_1
GCM_TINF2
Pathway
normal Y <-- NES --> LOY
Loss of Y
CN variations on Y
Average CN

Supplementary Figure 5. **a)** Copy-number plots across chromosome Y in male patients show heterogeneous loss of Y (n = 5 patients), copy number variations (n = 3 patients), gain of Y (n = 1 patient), and normal Y expression (n = 10 patients). **b)** ChrY signature score plotted against average chrY copy-number shows positive correlation between chrY copy number and chrY signature score. **c)** GSEA of multiple human gene sets shows significant enrichment of sets (nominal p-value < 0.05) involved in metastasis and invasion in LOY patients, versus tumor suppression in normal Y patients (note; GSEA run separately for each gene set collection [hallmark, c2-cgp, c4-cgn, c4-3ca], and specific gene sets from each selected for visualization). Significance assessed by FDR q-value < 0.05 (green).

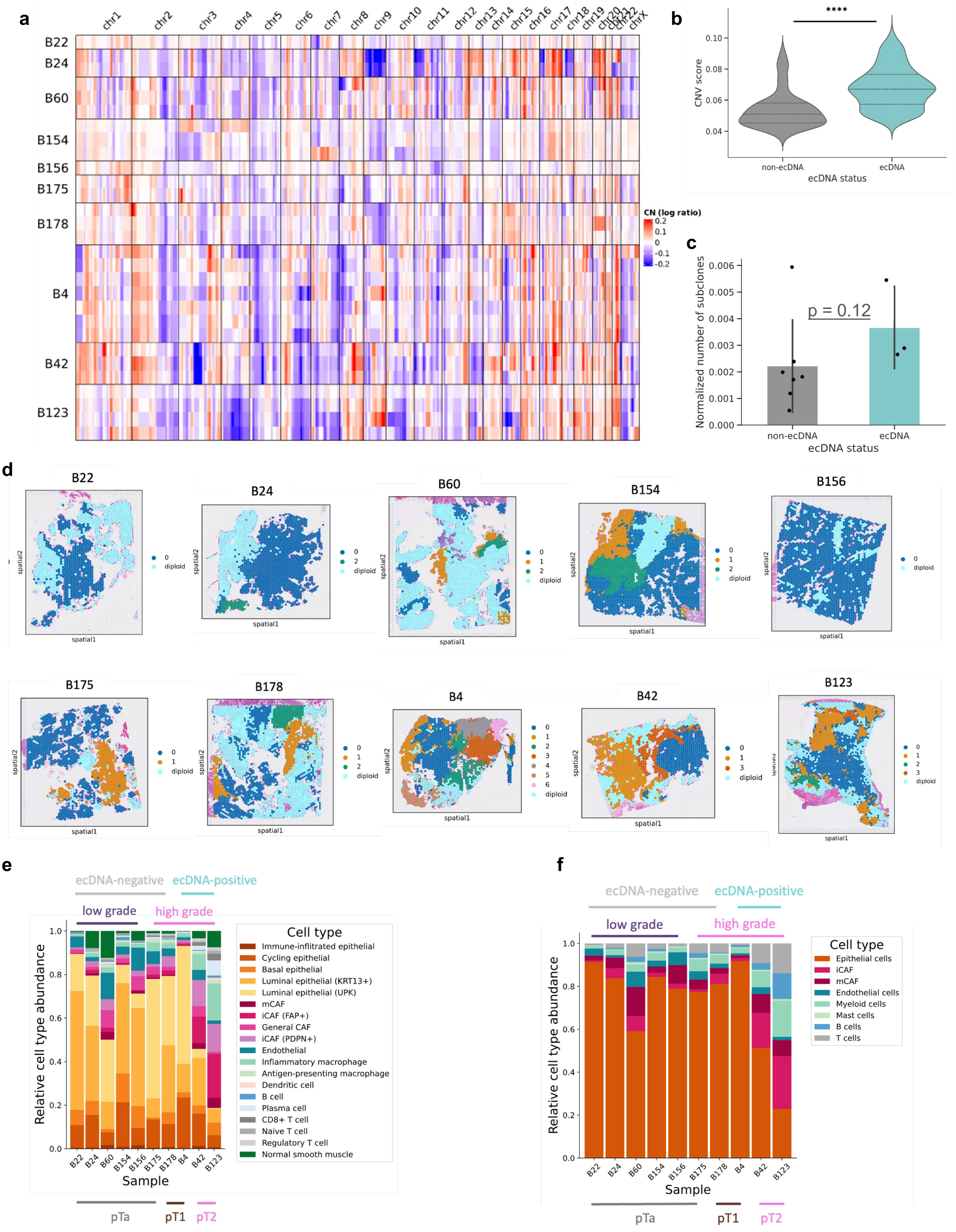
a
chr1 chr2 chr3 chr4 chr5 chr6 chr7 chr8 chr9 chr10 chr11 chr12 chr13 chr14 chr15 chr16 chr17 chr18 chr19 chr20 chr21 chr22 chrX
B22
B24
B60
B154
B156
B175
B178
B4
B42
B123
CN (log ratio)
0.2
0.1
0
-0.1
-0.2
b
****
CNV score
0.10
0.08
0.06
0.04
non-ecDNA
ecDNA
ecDNA status
c
Normalized number of subclones
0.006
0.005
0.004
0.003
0.002
0.001
0.000
p = 0.12
non-ecDNA
ecDNA
ecDNA status
d
B22
B24
B60
B154
B156
B175
B178
B4
B42
B123
spatial1
spatial2
0
1
2
3
4
5
6
diploid
e
ecDNA-negative
ecDNA-positive
low grade
high grade
Relative cell type abundance
1.0
0.8
0.6
0.4
0.2
0.0
Sample
B22 B24 B60 B154 B156 B175 B178 B4 B42 B123
Cell type
Immune-infiltrated epithelial
Cycling epithelial
Basal epithelial
Luminal epithelial (KRT13+)
Luminal epithelial (UPK)
mCAF
iCAF (FAP+)
General CAF
iCAF (PDPN+)
Endothelial
Inflammatory macrophage
Antigen-presenting macrophage
Dendritic cell
B cell
Plasma cell
CD8+ T cell
Naive T cell
Regulatory T cell
Normal smooth muscle
pTa
pT1
pT2
f
ecDNA-negative
ecDNA-positive
low grade
high grade
Relative cell type abundance
1.0
0.8
0.6
0.4
0.2
0.0
Sample
B22 B24 B60 B154 B156 B175 B178 B4 B42 B123
Cell type
Epithelial cells
iCAF
mCAF
Endothelial cells
Myeloid cells
Mast cells
B cells
T cells
pTa
pT1
pT2

Supplementary Figure 6. Copy-number inference and cell type deconvolution from 10 Visium samples. a) Heatmap showing pseudobulk CNA profiles of all the detected subclones for each tumor. Number of detected subclones per sample: B22: 1, B24: 2, B60: 3, B154: 3, B156: 1, B175: 2, B178: 3, B4: 7, B42: 3, B123: 4. **b)** Comparison in the CNV score for all aneuploid Visium spots between ecDNA-negative (n=8,889 spots) and ecDNA-positive (n=3,828 spots) tumors. Two-sided Mann-Whitney-U test was used for statistical testing. P-value < 0.0001, rank-Biserial correlation (effect size) = 0.6. **c)** Comparison in the number of detected subclones from Visium between ecDNA-negative (n=7) and ecDNA-positive (n=3) tumors. Two-sided Mann-Whitney-U test was used for statistical testing. Rank-Biserial correlation (effect size) = 0.714. Height of the bars represent the mean, while error bars represent standard deviation. Each data point represents one tumor (non-ecDNA, n=7; ecDNA, n=3). **d)** Subclones identified by CopyKAT mapped to the Visium slides. Number of spots per sample: B22: 1231, B24: 1696, B60: 2307, B154: 2925, B156: 2108, B175: 1330, B178: 2748, B4: 1512, B42: 1424, B123: 2282. **e)** Cell type composition of the Visium cohort, following deconvolution of cell types with cell2location, using a single-nuclei RNAseq reference from Gouin III *et al.,* or **f)** from single-cell RNAseq reference from Chen *et al.*

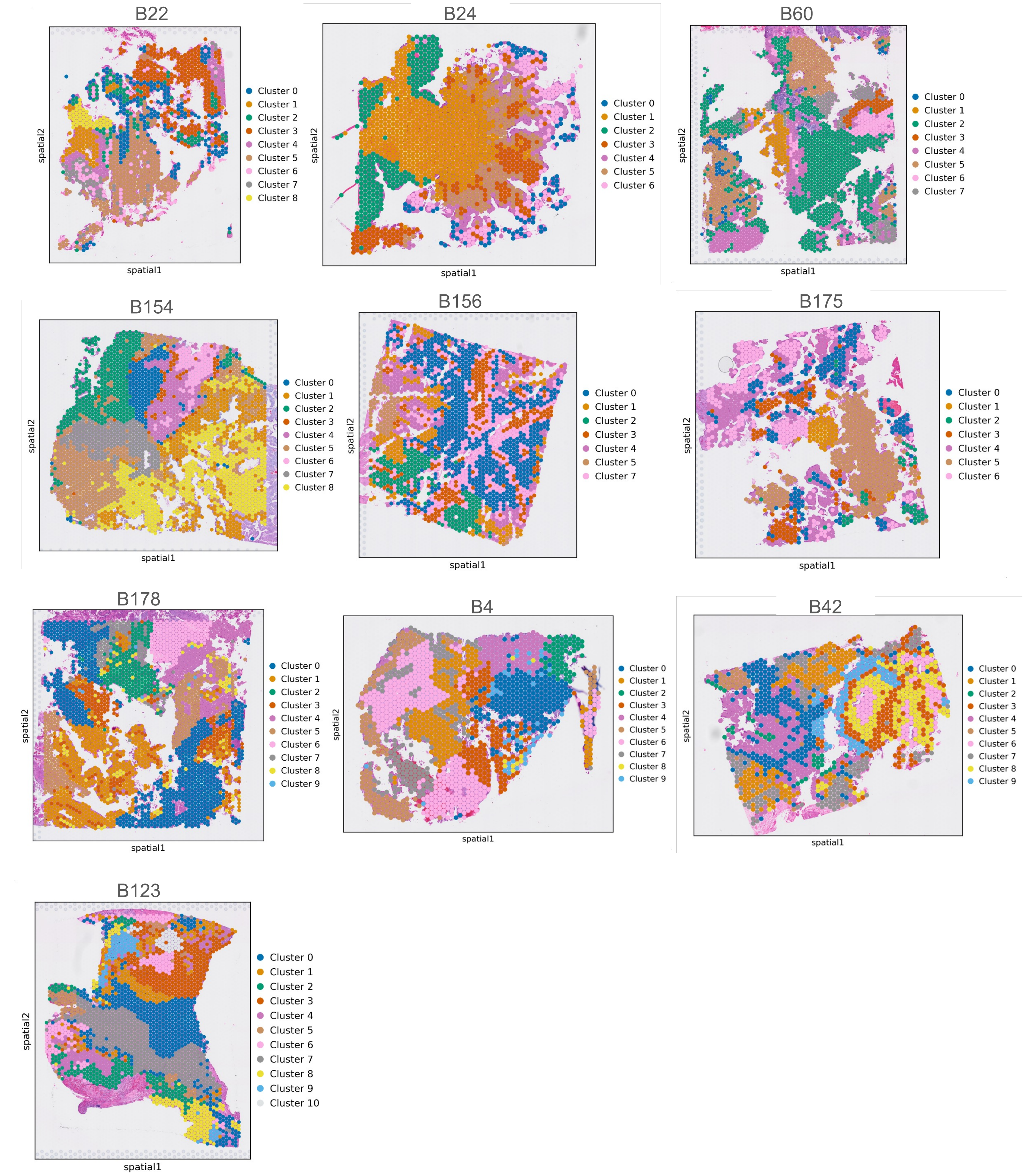

Supplementary Figure 7: **SpatialDE2 clusters in space for all Visium spots across all the samples.** Number of spots per sample: B22: 1231, B24: 1696, B60: 2307, B154: 2925, B156: 2108, B175: 1330, B178: 2748, B4: 1512, B42: 1424, B123: 2282.

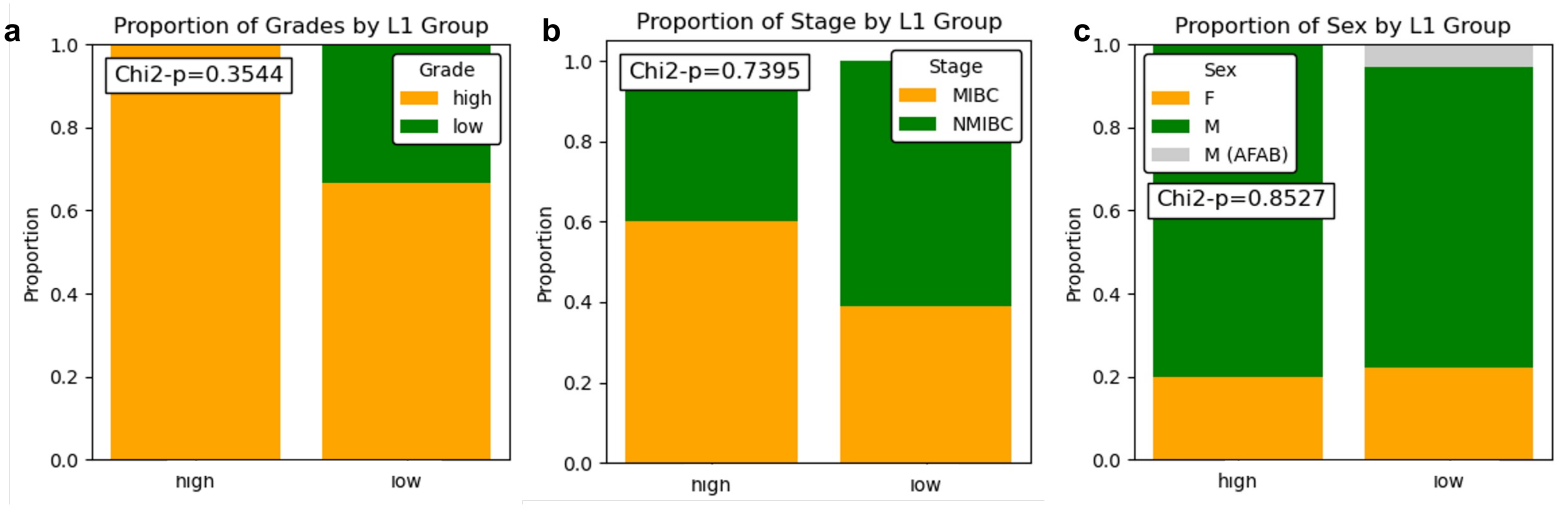

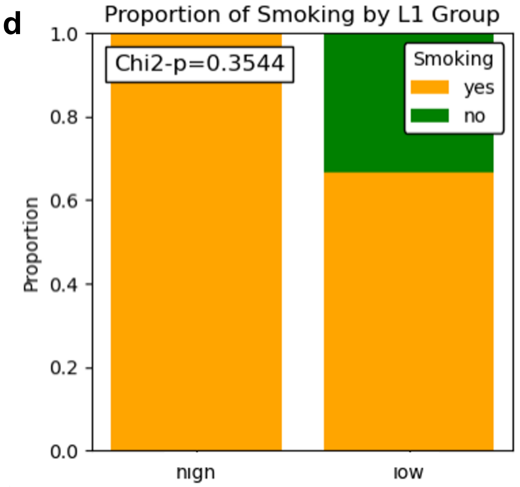

e. L1-high vs L1-low by clinical feature

Feature	Grade	Stage	Age	Sex	Smoking
Test	Mann-Whitney U-test, 2-sided	Mann-Whitney U-test, 2-sided	Spearman correlation	Mann-Whitney U-test, 2-sided	Mann-Whitney U-test, 2-sided
Statistic	U = 84.0, p = 0.020	U = 37.5, p = 0.086	r = 0.17, p = 0.451	U = 44.5, p = 0.905	U = 76.0, p = 0.079

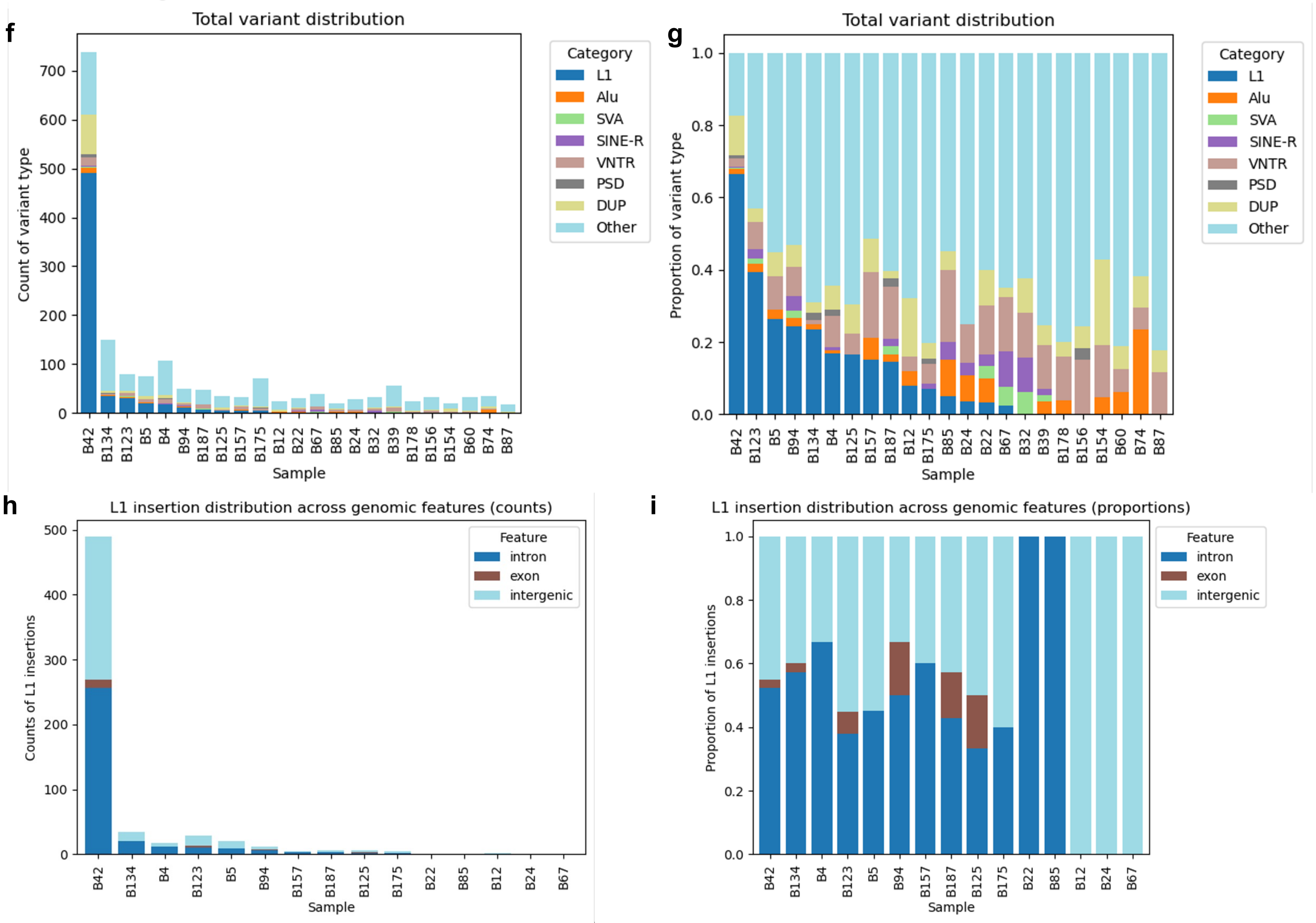

Supplementary Figure 8. a-d) Proportion of clinical features in L1-high and L1-low patients (L1-high = insertion count > 15: n = 5 patients, L1-low = insertion count < 5: n = 7 patients). Statistical significance evaluated with Chi-squared proportion test. **e)** L1 insertion count comparison across clinical feature groups (grade: high versus low, stage: MIBC versus NMIBC, sex: male versus female (AFAB patient excluded), smoking: yes versus no). Statistical significance evaluated with two-sided Mann-Whitney U-test for categorical variables (grade, stage, sex, smoking status) and Spearman correlation for continuous variables (age). **f-g)** Total variant distributions by count (f) and proportion (g) across all identified categories from long-read sequencing. h-i) L1 insertion genomic location (intron, exon, intergenic) distribution by count (h) and proportion (i).

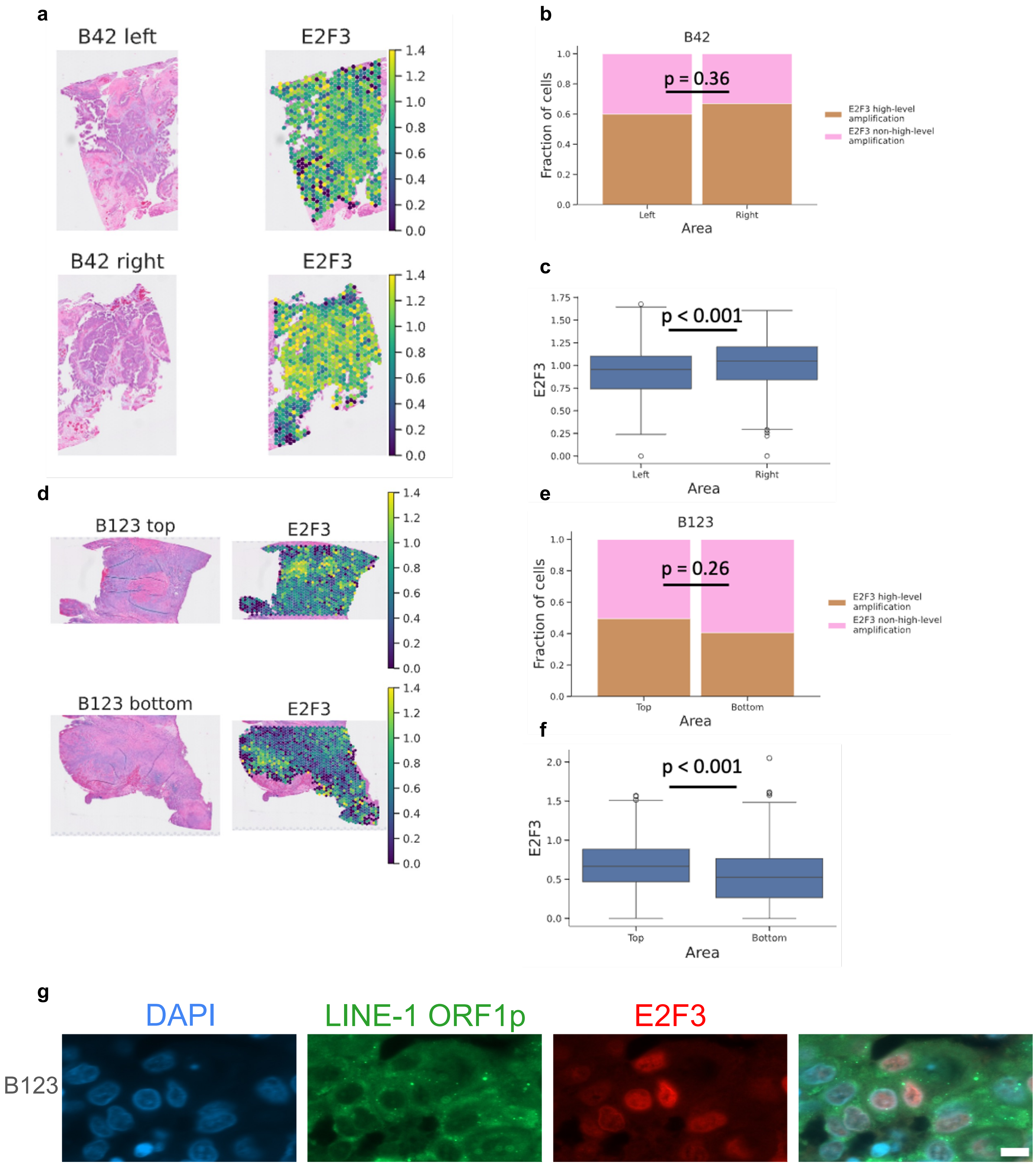

a
B42 left
E2F3
B42 right
E2F3
b
B42
p = 0.36
Fraction of cells
Area
Left
Right
E2F3 high-level amplification
E2F3 non-high-level amplification
c
p < 0.001
E2F3
Area
Left
Right
d
B123 top
E2F3
B123 bottom
E2F3
e
B123
p = 0.26
Fraction of cells
Area
Top
Bottom
E2F3 high-level amplification
E2F3 non-high-level amplification
f
p < 0.001
E2F3
Area
Top
Bottom
g
DAPI
LINE-1 ORF1p
E2F3
B123

Supplementary Figure 9. Quantification of high-level amplification of *E2F3* by FISH validate Visium analysis results. **a)** Two regions of sample B42 used for FISH (left) with normalised E2F3 expression from Visium (right). **b)** Fraction of cells with *E2F3* high-level amplification quantified by FISH for two distinct regions in sample B42. Chi-squared test was used for statistical testing, Cramér's V = 0.063. Number of counted cells per region: left, n=110; right, n=100. **c)** Normalised *E2F3* expression from Visium for two distinct regions in sample B42. Two-sided Mann-Whitney-U test was used for statistical testing. Rank-Biserial correlation (effect size) = 0.21. Number of spots per region: left, n=737; right, n=687. **d)** Two regions of tumor B123 used for FISH (left) with normalised *E2F3* expression from Visium (right). **e)** Fraction of cells with *E2F3* high-level amplification quantified by FISH for two distinct regions in sample B123. Chi-squared test was used for statistical testing, Cramér's V = 0.080. Number of counted cells per region: top, n=101; bottom, n=101. **f)** Normalised *E2F3* expression from Visium for two distinct region in sample B123. Two-sided Mann-Whitney-U test was used for statistical testing. Rank-Biserial correlation (effect size) = 0.234. Number of spots per region: top, n=1006; bottom, n=1276. **g)** Co-expression of LINE-1 ORF1p and E2F3 in the same cells. Representative immunofluorescence stainings for the sample B123 are shown. The experiment was repeated in one more patient with similar results. Scale bar: 10 µm. Boxes in panels c and f represent the interquartile range (IQR), with the center line indicating the median. Whiskers extend to 1.5× IQR.

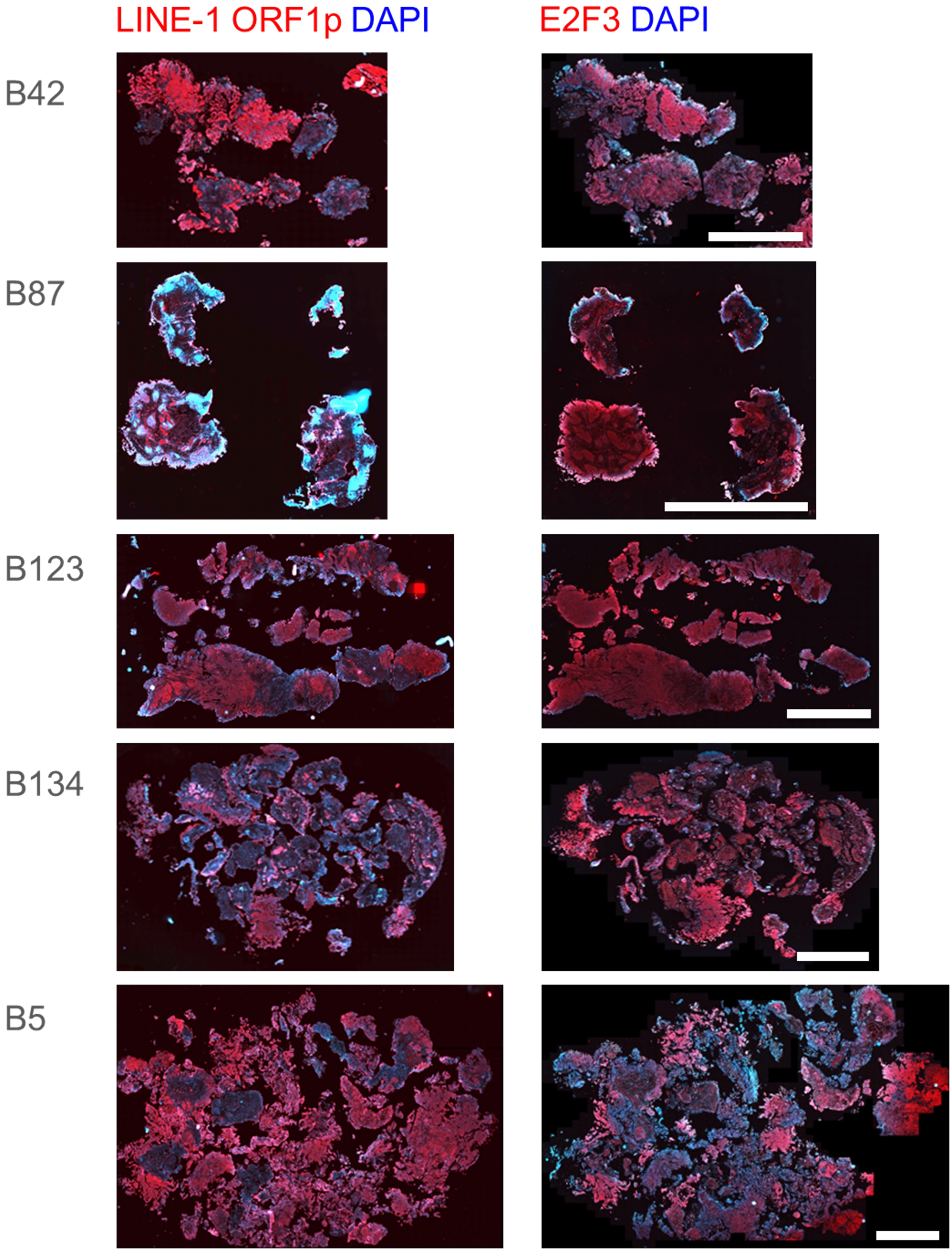

LINE-1 ORF1p DAPI
E2F3 DAPI
B42
B87
B123
B134
B5

Supplementary Figure 10. Expression of E2F3 (oncogene located on a ecDNA structure) and LINE-1 ORF1p in the same tissue regions of five patients. Scale bar: 500 μm.

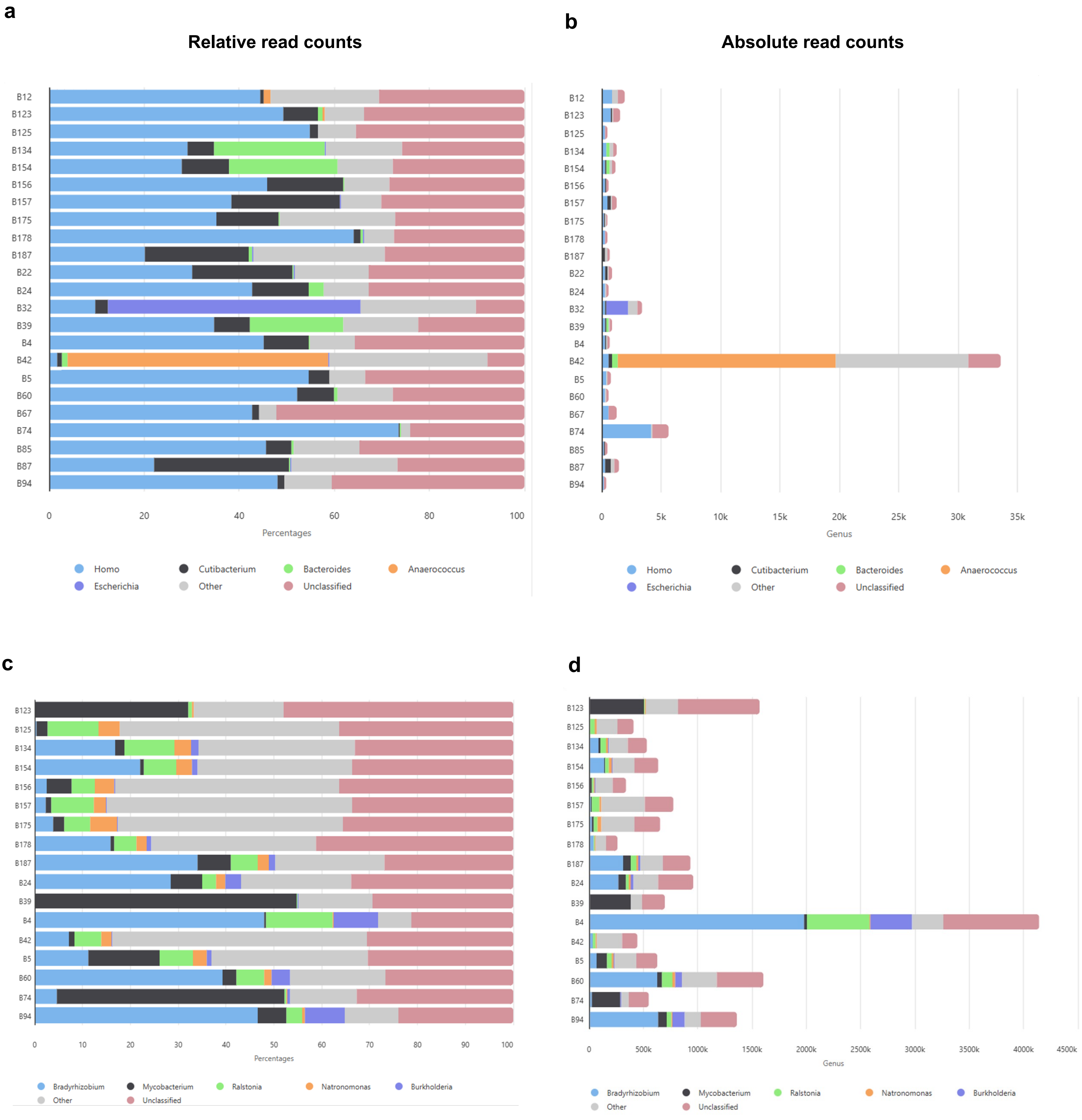

a
Relative read counts
B12
B123
B125
B134
B154
B156
B157
B175
B178
B187
B22
B24
B32
B39
B4
B42
B5
B60
B67
B74
B85
B87
B94
0
20
40
60
80
100
Percentages
Homo
Cutibacterium
Bacteroides
Anaerococcus
Escherichia
Other
Unclassified
b
Absolute read counts
0
5k
10k
15k
20k
25k
30k
35k
Genus
c
B123
B125
B134
B154
B156
B157
B175
B178
B187
B24
B39
B4
B42
B5
B60
B74
B94
0
10
20
30
40
50
60
70
80
90
100
Percentages
Bradyrhizobium
Mycobacterium
Ralstonia
Natronomonas
Burkholderia
Other
Unclassified
d
0
500k
1000k
1500k
2000k
2500k
3000k
3500k
4000k
4500k
Genus

Supplementary Figure 11. a) Percentages of unclassified and classified reads at the Genus taxonomy level of the long-read tumor WGS data (DNA-Seq). **b)** Absolute read counts of the long-read tumor WGS data (DNA-Seq). **c)** Percentages of unclassified and classified reads at the Genus taxonomy level of the bulk transcriptome data (RNA-Seq). **d)** Absolute read counts in the bulk transcriptome data. The analysis was performed on all unmapped reads from the tumor sample using kraken2.

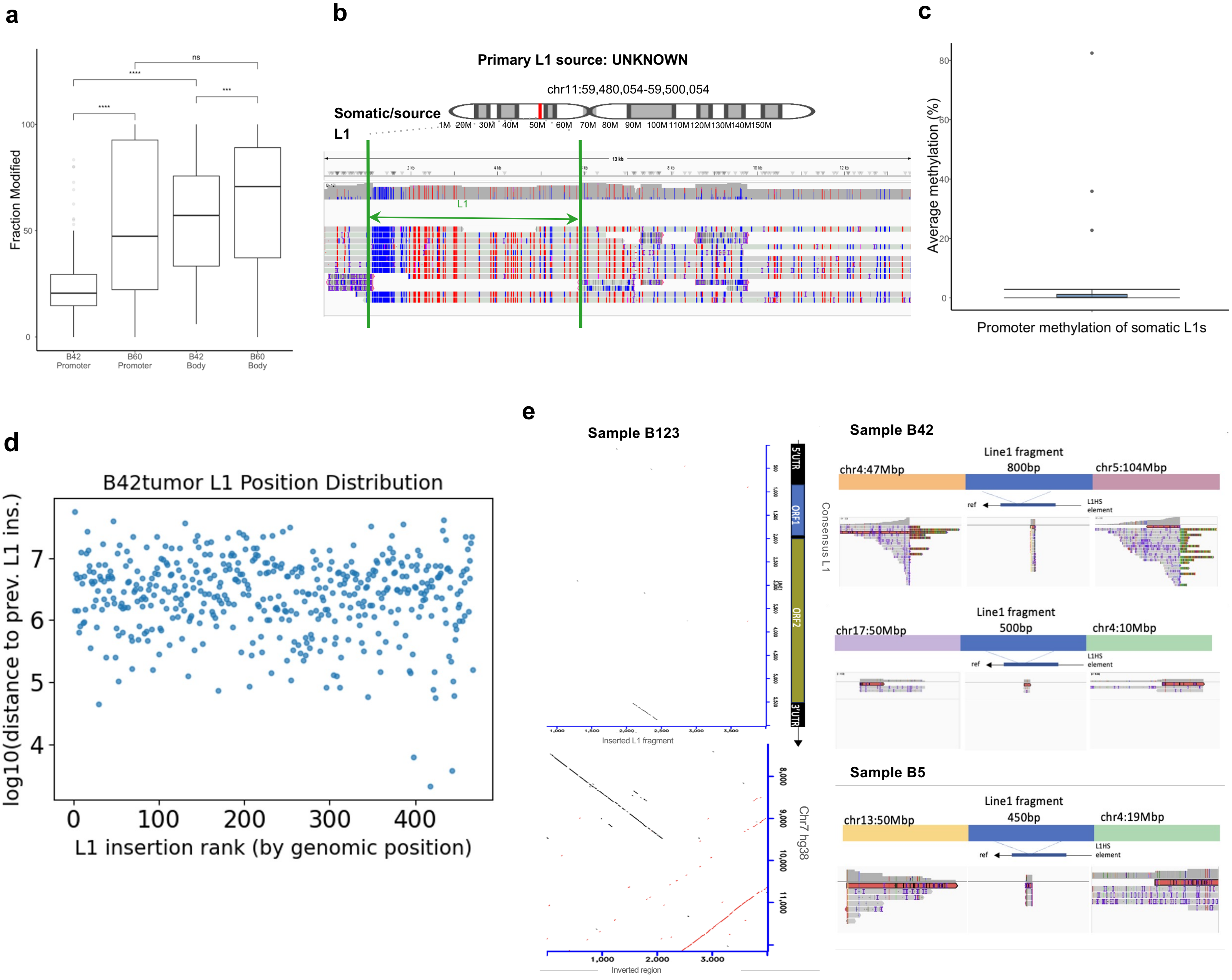

Supplementary Figure 12. a) Comparative CpG methylation profiles of hg38-annotated LINE-1 loci in patient samples B60 (low L1 activity, n CpG promoter = 205, n CpG body = 212) and B42 (high L1 activity, n CpG promoter = 205, n CpG body = 213) (***: p <= 0.001, ****:p <= 0.0001, ns: p > 0.05, Wilcoxon test). **b)** Second example of a multi-jump L1 event detected in the B42 tumor, visualized in IGV. A solo L1 insertion without any transduced sequence on chromosome 11 is visualized with unmethylated CpG sites shown in blue. **c)** Average promoter methylation of the full-length somatic L1 elements in B42 (n=16) **d)** Rainfall plot of L1 insertion positions across the genome in B42 tumor. “L1 insertion rank” indicates relative position of L1 insertion in ordered genome (i.e. rank 0 is the first L1 insertion found in the sample, rank 1 is the second L1 insertion found in the sample, etc.) **e)** Examples of a somatic L1 insertion embedded between somatic structural variant (SV) breakpoints in samples B123, B42 and B4. Source data are provided as a Source Data file.

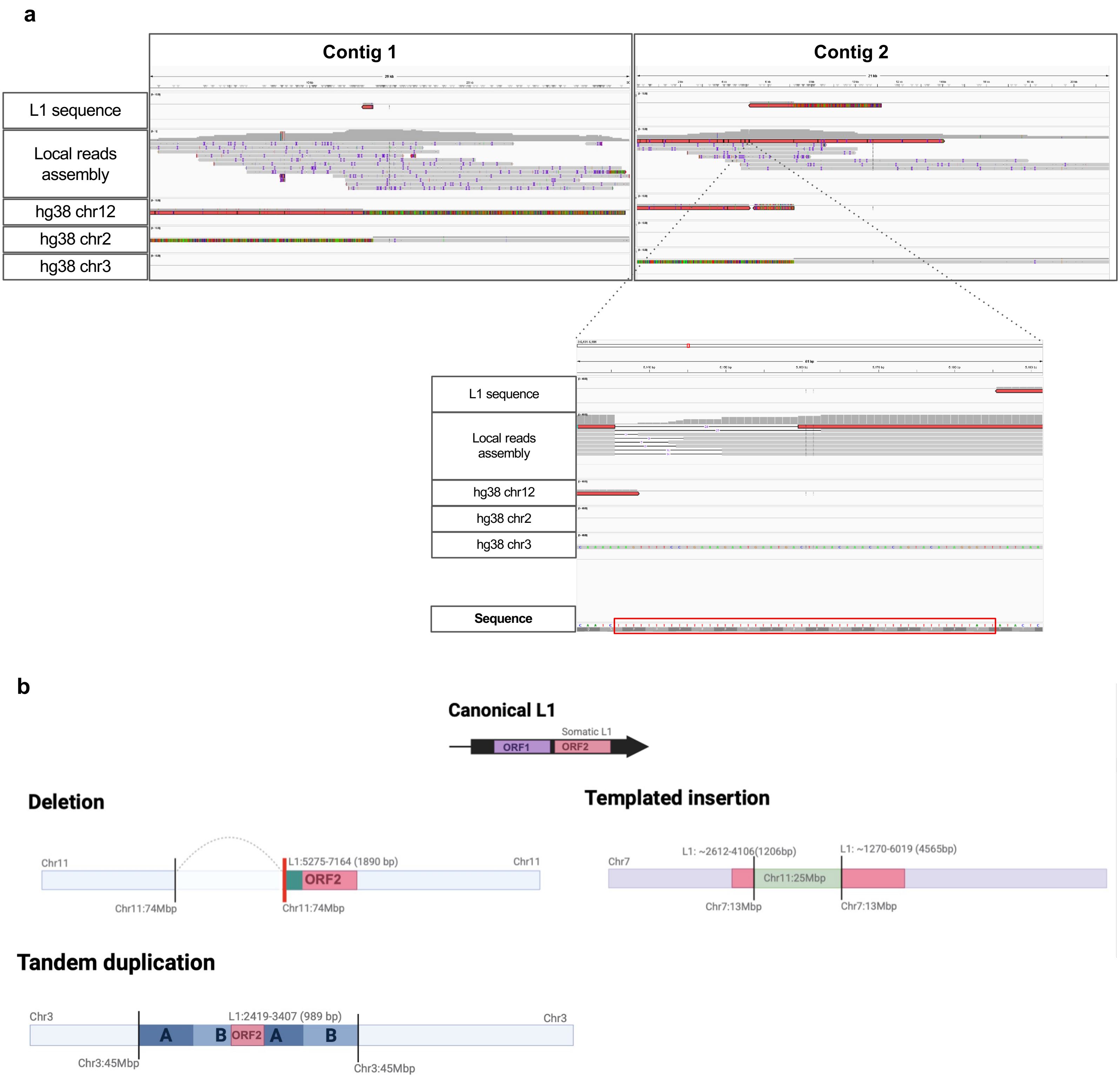

Supplementary Figure 13. a) Local read and L1-read assemblies supporting the L1-mediated interchromosomal translocation event shown in Figure 3B. Contigs were locally assembled, illustrating the rearranged structure and the contribution of L1-derived sequence within the breakpoint region. **b)** Schematic overview of additional L1-associated structural variants identified in patient B42 using Breaktracer, including deletions, tandem duplications, and templated insertions derived from internal L1 (ORF2) regions. The canonical L1 structure is shown for reference.

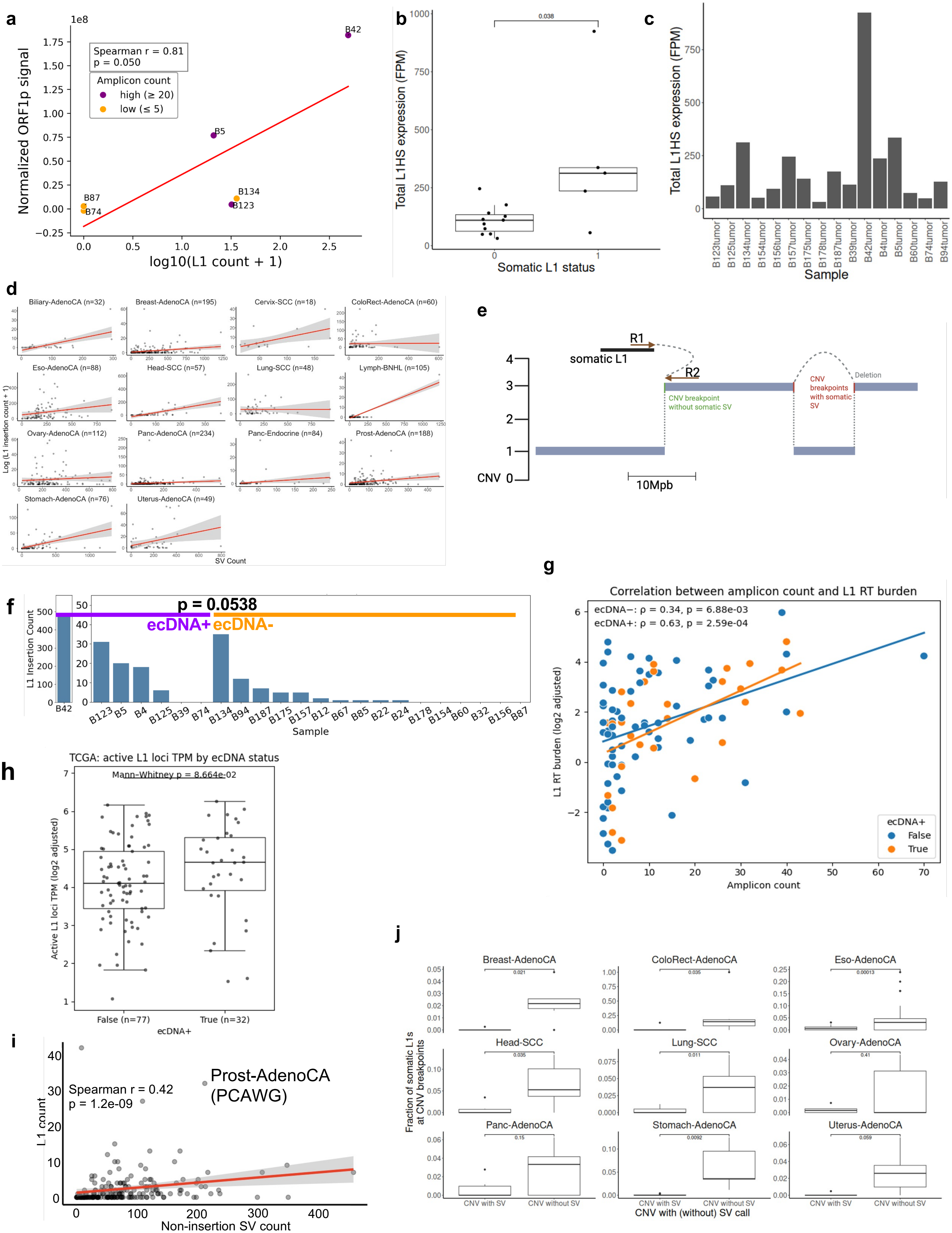

a
Spearman r = 0.81
p = 0.050
Amplicon count
high (≥ 20)
low (≤ 5)
Normalized ORF1p signal
log10(L1 count + 1)
B42
B5
B134
B123
B87
B74
b
0.038
Total L1HS expression (FPM)
Somatic L1 status
c
Total L1HS expression (FPM)
Sample
d
Biliary-AdenoCA (n=32)
Breast-AdenoCA (n=195)
Cervix-SCC (n=18)
ColoRect-AdenoCA (n=60)
Eso-AdenoCA (n=88)
Head-SCC (n=57)
Lung-SCC (n=48)
Lymph-BNHL (n=105)
Ovary-AdenoCA (n=112)
Panc-AdenoCA (n=234)
Panc-Endocrine (n=84)
Prost-AdenoCA (n=188)
Stomach-AdenoCA (n=76)
Uterus-AdenoCA (n=49)
Log (L1 insertion count + 1)
SV Count
e
R1
somatic L1
R2
CNV breakpoint without somatic SV
CNV breakpoints with somatic SV
Deletion
CNV
10Mpb
f
p = 0.0538
ecDNA+
ecDNA-
L1 Insertion Count
Sample
g
Correlation between amplicon count and L1 RT burden
ecDNA−: ρ = 0.34, p = 6.88e-03
ecDNA+: ρ = 0.63, p = 2.59e-04
L1 RT burden (log2 adjusted)
Amplicon count
ecDNA+
False
True
h
TCGA: active L1 loci TPM by ecDNA status
Mann-Whitney p = 8.664e-02
Active L1 loci TPM (log2 adjusted)
False (n=77)
True (n=32)
ecDNA+
i
Spearman r = 0.42
p = 1.2e-09
Prost-AdenoCA (PCAWG)
L1 count
Non-insertion SV count
j
Breast-AdenoCA
ColoRect-AdenoCA
Eso-AdenoCA
Head-SCC
Lung-SCC
Ovary-AdenoCA
Panc-AdenoCA
Stomach-AdenoCA
Uterus-AdenoCA
Fraction of somatic L1s at CNV breakpoints
CNV with SV
CNV without SV
CNV with (without) SV call

Supplementary Figure 14. a) Correlation between the number of LINE-1 insertions and ORF1p protein expression (signal intensity). Each data point shows one tumor. **b)** Total L1HS expression for L1-high samples (B123, B134, B4, B42, B5), labelled 1, compared to all other samples. **c)** Total L1HS expression values for each sample estimated using L1EM. **d)** Correlation between the number of L1 insertions and non-insertion SVs across PCAWG for tumors with more than 10 samples and at least one tumor with L1 count > 25. **e)** Schematic overview of the classification of CNV breakpoints as SV-unexplained or SV-explained based on their overlap (+/-5kbp) with somatic L1 insertions in the PCAWG dataset. Created in bioRender. **f)** Counts of L1 elements across tumors. ecDNA-positive tumors (n = 7 patients) show a significant enrichment for L1 insertions versus ecDNA-negative tumors (n = 16 patients) ($p = 0.0538$, Rank-biserial effect size = 0.429, one-sided Mann-Whitney U-test). **g)** Correlation between amplicon count and L1 retrotranscription (RT) burden in TCGA patients. Color split by ecDNA status (blue = ecDNA-negative, orange = ecDNA-positive). **h)** Active L1 loci TPM (log2 adjusted) for ecDNA-positive versus ecDNA-negative TCGA patients. Boxes represent the interquartile range (IQR), with the center line indicating the median. Whiskers extend to 1.5× IQR. Points represent individual samples. **i)** Correlation between the number of L1 insertions and non-insertion SVs across PCAWG for Prost-AdenoCA (n samples = 188). **j)** The overlap of somatic L1 insertions with CNV breakpoints computed using a ±5 kbp window for each category (CNVs with and without SV support), and the fractions of somatic L1s proximal to CNV breakpoints were compared across PCAWG tumor histologies.

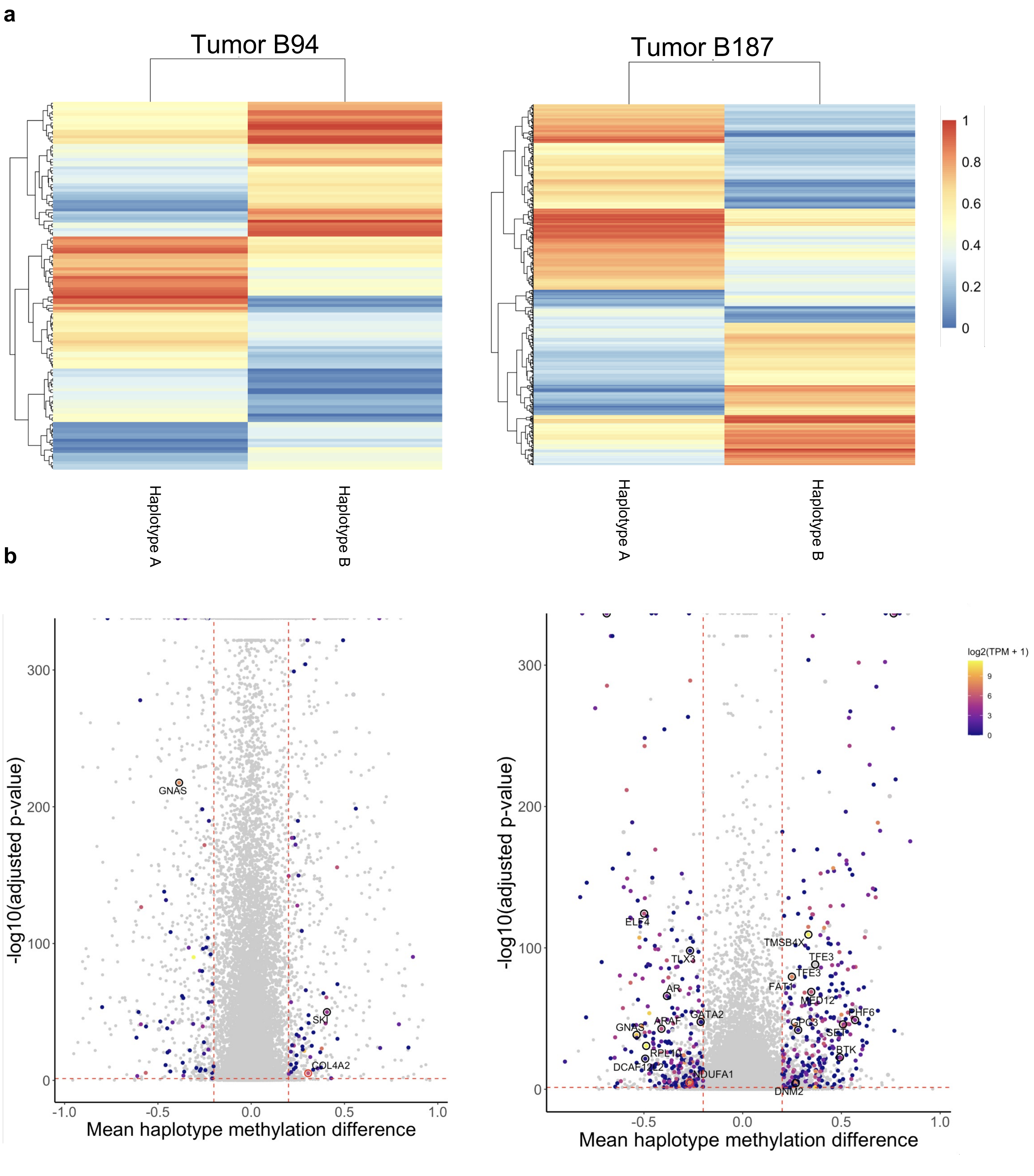

Supplementary Figure 15. a) Heatmap of promoters exhibiting significant differential methylation (q < 0.05; Benjamini–Hochberg–corrected binomial test) in samples B94 (n promoters = 131) and B187 (n promoters = 570). **b)** Volcano plots corresponding to the samples shown in a). Genes with significantly differentially methylated promoters are colored according to normalized gene expression levels. Genes harboring single-nucleotide variants (SNVs) are labeled in red, while genes listed in the COSMIC database are labeled in black.

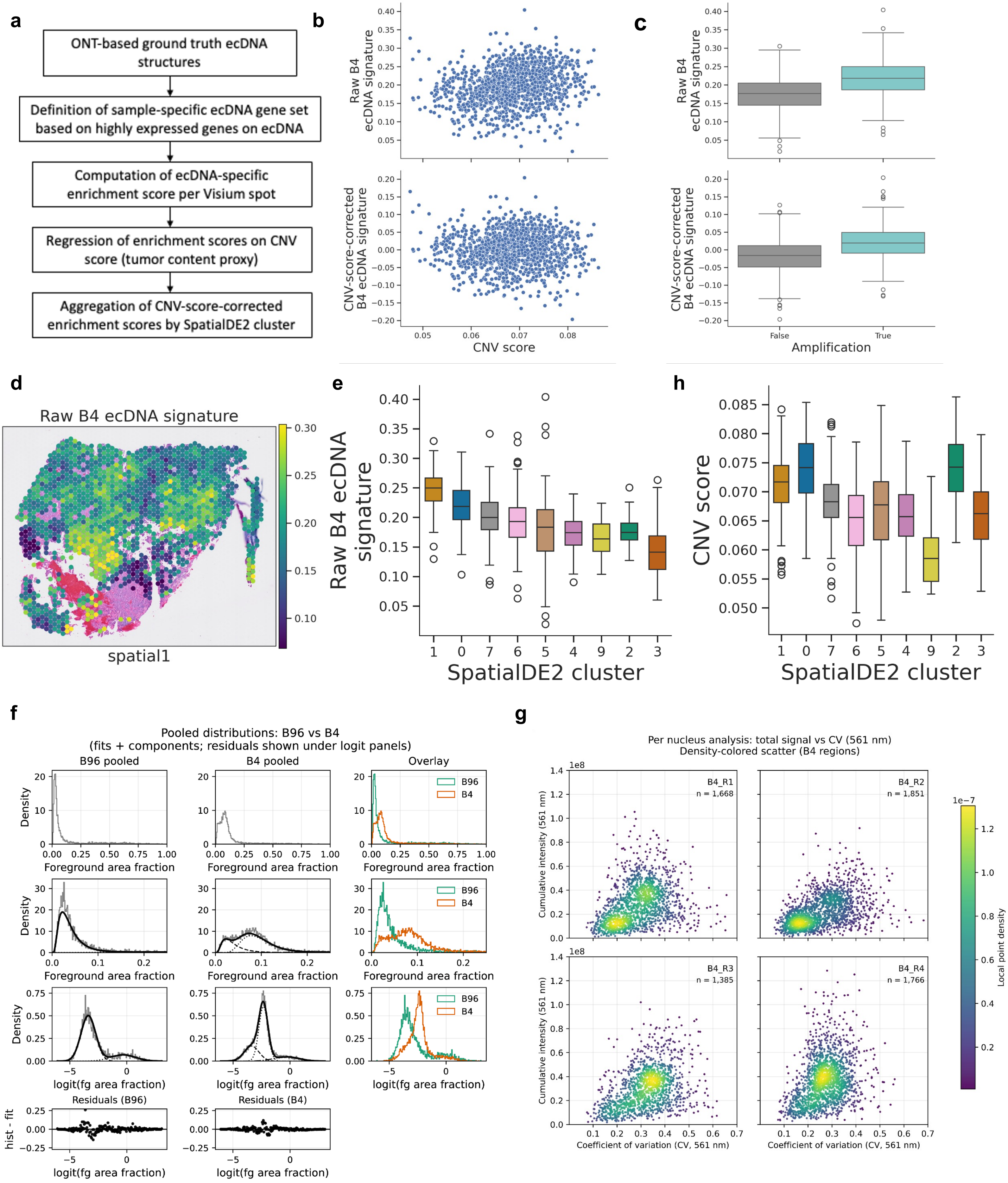
a
ONT-based ground truth ecDNA structures
Definition of sample-specific ecDNA gene set based on highly expressed genes on ecDNA
Computation of ecDNA-specific enrichment score per Visium spot
Regression of enrichment scores on CNV score (tumor content proxy)
Aggregation of CNV-score-corrected enrichment scores by SpatialDE2 cluster
b
Raw B4 ecDNA signature
CNV-score-corrected B4 ecDNA signature
CNV score
c
Raw B4 ecDNA signature
CNV-score-corrected B4 ecDNA signature
False
True
Amplification
d
Raw B4 ecDNA signature
spatial2
spatial1
e
Raw B4 ecDNA signature
SpatialDE2 cluster
h
CNV score
SpatialDE2 cluster
f
Pooled distributions: B96 vs B4
(fits + components; residuals shown under logit panels)
B96 pooled
B4 pooled
Overlay
Density
Foreground area fraction
B96
B4
logit(fg area fraction)
Residuals (B96)
Residuals (B4)
hist - fit
g
Per nucleus analysis: total signal vs CV (561 nm)
Density-colored scatter (B4 regions)
B4_R1
n = 1,668
B4_R2
n = 1,851
B4_R3
n = 1,385
B4_R4
n = 1,766
Cumulative intensity (561 nm)
Coefficient of variation (CV, 561 nm)
Local point density
1e8
1e−7

Supplementary Figure 16. Identification of ecDNA-enriched spatial regions in Visium data for sample B4. a) Schematic overview of the approach to identify ecDNA-enriched spatial regions in Visium data. **b)** Top, relationship between CNV score and raw ecDNA-specific signature enrichment score. Bottom, relationship between CNV score and ecDNA-specific signature enrichment score after regressing out the CNV score on a per-spot basis. **c)** Top: comparison of raw ecDNA-specific signature enrichment scores between spots without inferred amplification spots with inferred amplification at the ecDNA genomic region. Bottom: the same comparison using CNV-score-corrected ecDNA-specific signature enrichment scores. **d)** Raw ecDNA-specific signature enrichment scores in space. **e)** Raw ecDNA-specific signature enrichment scores across all aneuploid spots, grouped by SpatialDE2 cluster. **f)** Quantification of *MDM2* FISH signals showing the distributions of foreground area fraction for pooled B96 and B4 regions, together with Gaussian mixture model (GMM) fits. **g)** Per-nucleus analysis of *MDM2* FISH signal across different regions (R1-R4) in sample B4. The x-axis represents the coefficient of variation (CV) of the *MDM2* signal within each nucleus, while the y-axis shows the cumulative signal intensity per nucleus. Nuclei with high CV and high total signal are consistent with ecDNA-positive cells, while nuclei with lower CV and lower total signal are consistent with ecDNA-negative cells. **h)** CNV score for all aneuploid spots, grouped by SpatialDE2 cluster.

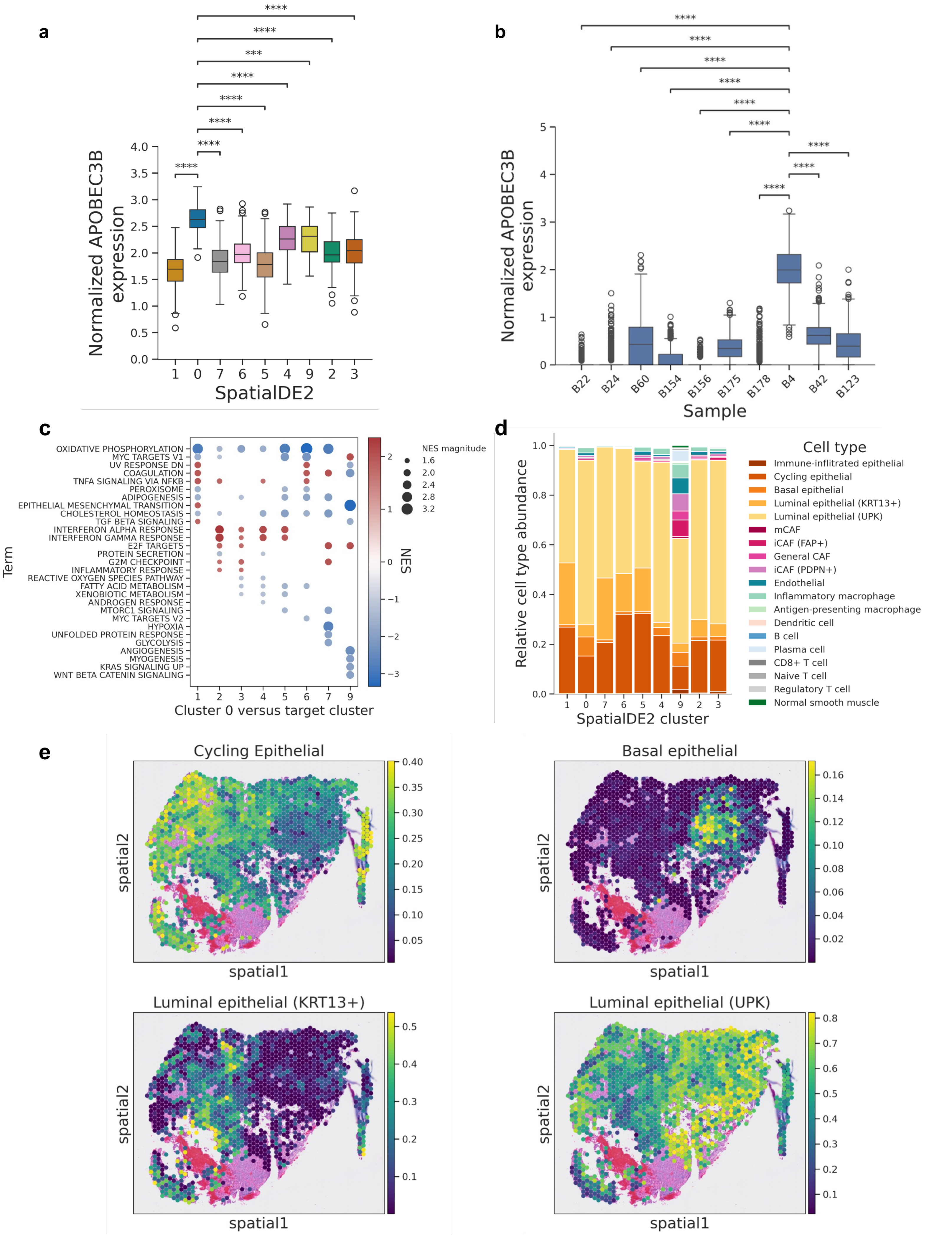
a
Normalized APOBEC3B expression
SpatialDE2
1 0 7 6 5 4 9 2 3
****
***
b
Normalized APOBEC3B expression
Sample
B22 B24 B60 B154 B156 B175 B178 B4 B42 B123
c
OXIDATIVE PHOSPHORYLATION
MYC TARGETS V1
UV RESPONSE DN
COAGULATION
TNFA SIGNALING VIA NFKB
PEROXISOME
ADIPOGENESIS
EPITHELIAL MESENCHYMAL TRANSITION
CHOLESTEROL HOMEOSTASIS
TGF BETA SIGNALING
INTERFERON ALPHA RESPONSE
INTERFERON GAMMA RESPONSE
E2F TARGETS
PROTEIN SECRETION
G2M CHECKPOINT
INFLAMMATORY RESPONSE
REACTIVE OXYGEN SPECIES PATHWAY
FATTY ACID METABOLISM
XENOBIOTIC METABOLISM
ANDROGEN RESPONSE
MTORC1 SIGNALING
MYC TARGETS V2
HYPOXIA
UNFOLDED PROTEIN RESPONSE
GLYCOLYSIS
ANGIOGENESIS
MYOGENESIS
KRAS SIGNALING UP
WNT BETA CATENIN SIGNALING
Term
Cluster 0 versus target cluster
NES
NES magnitude
1.6
2.0
2.4
2.8
3.2
d
Relative cell type abundance
SpatialDE2 cluster
Cell type
Immune-infiltrated epithelial
Cycling epithelial
Basal epithelial
Luminal epithelial (KRT13+)
Luminal epithelial (UPK)
mCAF
iCAF (FAP+)
General CAF
iCAF (PDPN+)
Endothelial
Inflammatory macrophage
Antigen-presenting macrophage
Dendritic cell
B cell
Plasma cell
CD8+ T cell
Naive T cell
Regulatory T cell
Normal smooth muscle
e
Cycling Epithelial
Basal epithelial
Luminal epithelial (KRT13+)
Luminal epithelial (UPK)
spatial1
spatial2

Supplementary Figure 17. ecDNA-enriched spatial clusters show different cell type composition in sample B4. **a)** Comparison of the normalized expression of *APOBEC3B* between SpatialDE2 clusters. A non-parametric Kruskal–Wallis test was used for statistical testing, followed by Dunn's test with Benjamini–Hochberg correction for multiple testing. For clarity, only statistical comparisons between each cluster and cluster 0 are shown. **b)** Normalized expression of *APOBEC3B* per spot for all 10 Visium samples. A non-parametric Kruskal–Wallis test was used for statistical testing, followed by Dunn's test with Benjamini–Hochberg correction for multiple testing. Number of spots per sample: B22, n=504; B24, n=1171; B60, n=505; B154, n=2525; B156, n=1826; B175, n=1102; B178, n=1256; B4, n=1284; B42, n=1037; B123, n=1507. **c)** GSEA on Hallmark pathways comparing cluster 0 to each SpatialDE2 cluster. For each cluster, the top 10 significantly enriched pathways (ranked by normalized enrichment score, NES) are shown. Dot size reflects the magnitude of the NES and color indicates the direction of enrichment (positive NES = higher in cluster 0, negative NES = higher in target cluster). **d)** Cell type composition of SpatialDE2 clusters. **e)** Fraction of epithelial subtypes across all aneuploid spots.

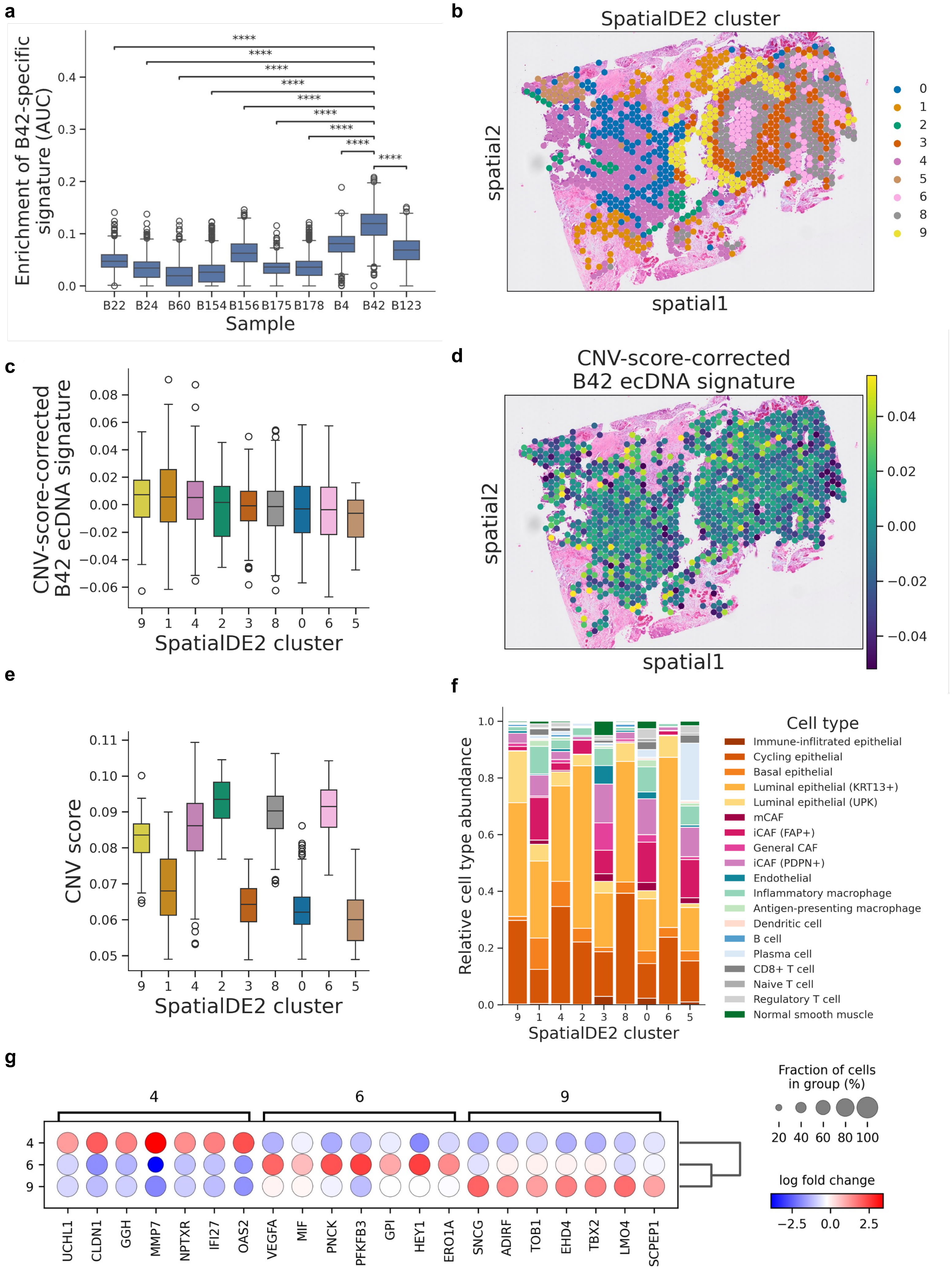

a
Enrichment of B42-specific signature (AUC)
****
Sample
B22 B24 B60 B154 B156 B175 B178 B4 B42 B123
b
SpatialDE2 cluster
spatial1
spatial2
0 1 2 3 4 5 6 8 9
c
CNV-score-corrected B42 ecDNA signature
SpatialDE2 cluster
d
CNV-score-corrected B42 ecDNA signature
spatial1
spatial2
e
CNV score
SpatialDE2 cluster
f
Relative cell type abundance
SpatialDE2 cluster
Cell type
Immune-inflitrated epithelial
Cycling epithelial
Basal epithelial
Luminal epithelial (KRT13+)
Luminal epithelial (UPK)
mCAF
iCAF (FAP+)
General CAF
iCAF (PDPN+)
Endothelial
Inflammatory macrophage
Antigen-presenting macrophage
Dendritic cell
B cell
Plasma cell
CD8+ T cell
Naive T cell
Regulatory T cell
Normal smooth muscle
g
UCHL1 CLDN1 GGH MMP7 NPTXR IFI27 OAS2 VEGFA MIF PNCK PFKFB3 GPI HEY1 ERO1A SNCG ADIRF TOB1 EHD4 TBX2 LMO4 SCPEP1
Fraction of cells in group (%)
20 40 60 80 100
log fold change
−2.5 0.0 2.5

Supplementary Figure 18. **Identification of ecDNA-enriched spatial regions in Visium data for sample B42. a)** AUC scores (enrichment) of the gene set consisting of highly expressed genes on ecDNA in tumor B42, computed for each aneuploid spot in all 10 Visium samples. A non-parametric Kruskal–Wallis test was used for statistical testing, followed by Dunn's test with Benjamini–Hochberg correction for multiple testing. Number of aneuploid spots per sample: B22, n=504; B24, n=1171; B60, n=505; B154, n=2525; B156, n=1826; B175, n=1102; B178, n=1256; B4, n=1284; B42, n=1037; B123, n=1507. **b)** Spatial clusters detected by SpatialDE2. Number of aneuploid spots per cluster: '0', n=148; '1', n=102; '2', n=36; '3', n=125; '4', n=208; '5', n=28; '6', n=86; '7', n=3; '8', n=197; '9', n=104. **c)** CNV-score-corrected ecDNA-specific signature enrichment score for all aneuploid spots, grouped by SpatialDE2 cluster. **d)** CNV-score-corrected ecDNA-specific signature enrichment score in space. **e)** CNV scores for all aneuploid spots, grouped by SpatialDE2 cluster. **f)** Cell type composition of SpatialDE2 clusters. **g)** Top 7 differentially expressed genes comparing two clusters with high ecDNA enrichment (9, 4) and one with low ecDNA enrichment (6), while having comparable CNV scores.

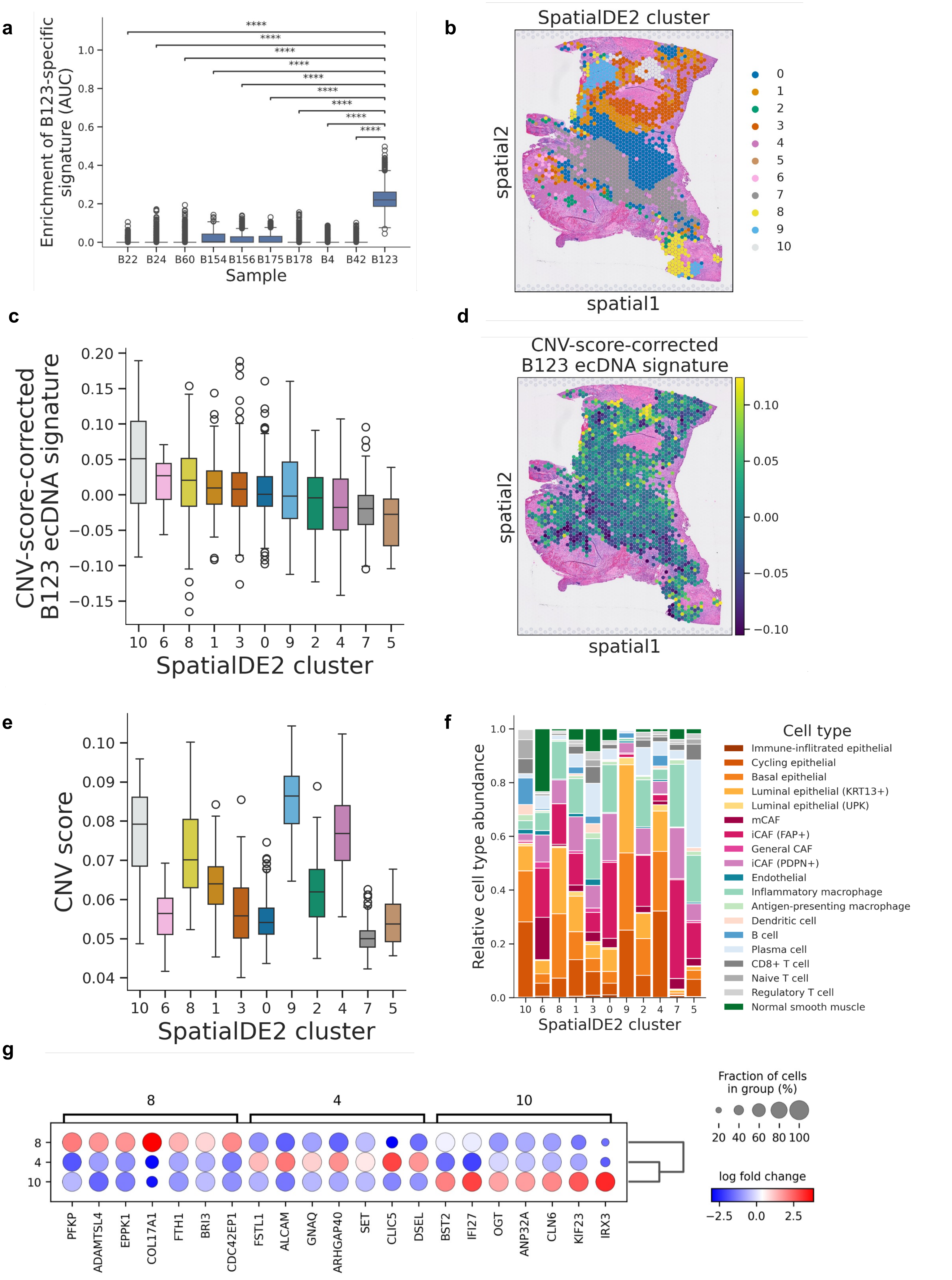

a
Enrichment of B123-specific signature (AUC)
****
B22 B24 B60 B154 B156 B175 B178 B4 B42 B123
Sample
b
SpatialDE2 cluster
spatial2
spatial1
0 1 2 3 4 5 6 7 8 9 10
c
CNV-score-corrected B123 ecDNA signature
SpatialDE2 cluster
10 6 8 1 3 0 9 2 4 7 5
d
CNV-score-corrected B123 ecDNA signature
spatial2
spatial1
e
CNV score
SpatialDE2 cluster
f
Relative cell type abundance
SpatialDE2 cluster
Cell type
Immune-inflitrated epithelial
Cycling epithelial
Basal epithelial
Luminal epithelial (KRT13+)
Luminal epithelial (UPK)
mCAF
iCAF (FAP+)
General CAF
iCAF (PDPN+)
Endothelial
Inflammatory macrophage
Antigen-presenting macrophage
Dendritic cell
B cell
Plasma cell
CD8+ T cell
Naive T cell
Regulatory T cell
Normal smooth muscle
g
8
4
10
Fraction of cells in group (%)
20 40 60 80 100
log fold change
−2.5 0.0 2.5
PFKP
ADAMTSL4
EPPK1
COL17A1
FTH1
BRI3
CDC42EP1
FSTL1
ALCAM
GNAQ
ARHGAP40
SET
CLIC5
DSEL
BST2
IFI27
OGT
ANP32A
CLN6
KIF23
IRX3

Supplementary Figure 19. Identification of ecDNA-enriched spatial regions in Visium data for sample B123. a) AUC scores (enrichment) of the gene set consisting of highly expressed genes on ecDNA in tumor B123, computed for each aneuploid spot in all 10 Visium samples. A non-parametric Kruskal–Wallis test was used for statistical testing, followed by Dunn's test with Benjamini–Hochberg correction for multiple testing. Number of aneuploid spots per sample: B22, n=504; B24, n=1171; B60, n=505; B154, n=2525; B156, n=1826; B175, n=1102; B178, n=1256; B4, n=1284; B42, n=1037; B123, n=1507. **b)** Spatial clusters detected by SpatialDE2. Number of aneuploid spots per cluster: '0', n=350; '1', n=173; '2', n=45; '3', n=211; '4', n=121; '5', n=16; '6', n=26; '7', n=347; '8', n=91; '9', n=84; '10', n=43. **c)** CNV-score-corrected ecDNA-specific signature enrichment score for all aneuploid spots, grouped by SpatialDE2 cluster. **d)** CNV-score-corrected ecDNA-specific signature enrichment score in space. **e)** CNV scores for all aneuploid spots, grouped by SpatialDE2 cluster. f) Cell type composition of SpatialDE2 clusters. **g)** Top 7 differentially expressed genes comparing two clusters with high ecDNA enrichment (10, 8) and one with low ecDNA enrichment (4), while having comparable CNV scores.

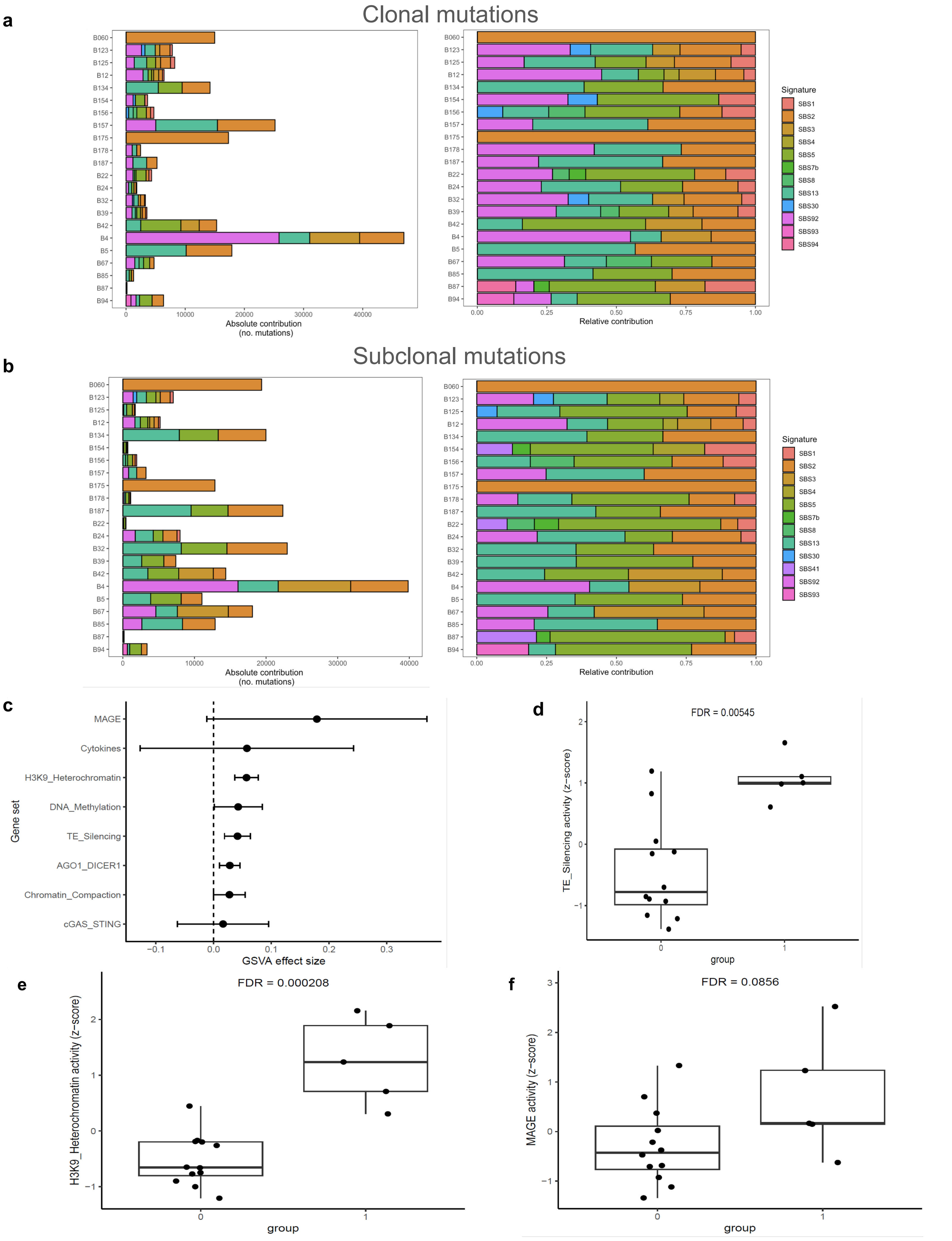
a
Clonal mutations
B060
B123
B125
B12
B134
B154
B156
B157
B175
B178
B187
B22
B24
B32
B39
B42
B4
B5
B67
B85
B87
B94
0
10000
20000
30000
40000
Absolute contribution
(no. mutations)
0.00
0.25
0.50
0.75
1.00
Relative contribution
Signature
SBS1
SBS2
SBS3
SBS4
SBS5
SBS7b
SBS8
SBS13
SBS30
SBS92
SBS93
SBS94
b
Subclonal mutations
Absolute contribution
(no. mutations)
Relative contribution
Signature
SBS1
SBS2
SBS3
SBS4
SBS5
SBS7b
SBS8
SBS13
SBS30
SBS41
SBS92
SBS93
c
MAGE
Cytokines
H3K9_Heterochromatin
DNA_Methylation
TE_Silencing
AGO1_DICER1
Chromatin_Compaction
cGAS_STING
Gene set
−0.1
0.0
0.1
0.2
0.3
GSVA effect size
d
FDR = 0.00545
TE_Silencing activity (z-score)
group
e
FDR = 0.000208
H3K9_Heterochromatin activity (z-score)
group
f
FDR = 0.0856
MAGE activity (z-score)
group

Supplementary Figure 20. a) Relative and absolute contribution of each COSMIC signature in the patient cohort for clonal mutations. **b)** Relative and absolute contribution of each COSMIC signature in the patient cohort for subclonal mutations. **c)** Gene set tests using Camera and GSVA between the L1-high samples (B123, B134, B4, B42, B5) and all other samples. **d-f)** Gene Set Variation Analysis (GSVA) of various gene sets comparing L1-high samples (B123, B134, B4, B42, B5) to all other samples for TE silencing activity (d), H3K9 heterochromatin activity (e), and MAGE activity (f).
