# Supplementary material for: Multiomic profiling links L1 retrotransposition to genomic instability and ecDNA in bladder cancer
Source: Nat Commun. 2026 Jul 16;17:6384. doi: 10.1038/s41467-026-75399-6 (PMC13376730; doi:10.1038/s41467-026-75399-6)
Supplement: Supplementary file 2 — Description of Additional Supplementary Files [file 41467_2026_75399_MOESM2_ESM.pdf]

Descriptions of Additional Supplementary Files

Supplementary Data 1: Cohort information, including clinical data, available data types, and sample quality metrics

Supplementary Data 2: Structural variants

Supplementary Data 3: Single nucleotide variants

Supplementary Data 4: Gene fusions

Supplementary Data 5: ecDNA descriptions

Supplementary Data 6: Top 20 marker genes for SpatialDE2 clusters per sample

Supplementary Data 7: Novel source L1s

Supplementary Data 8: Hot source L1s

Supplementary Data 9: TP53 mutations

Supplementary Data 10: Sequencing metrics for germline data

Supplementary Data 11: Sequencing metrics for ONT

Supplementary Data 12: L1 insertions with length, strand, read coverage and insertion support information
