# Supplementary material for: Multiomic profiling links L1 retrotransposition to genomic instability and ecDNA in bladder cancer
Source: Nat Commun. 2026 Jul 16;17:6384. doi: 10.1038/s41467-026-75399-6 (PMC13376730; doi:10.1038/s41467-026-75399-6)
Supplement: Supplementary file 4 — Reporting Summary [file 41467_2026_75399_MOESM4_ESM.pdf]

Corresponding author(s): Jan O. Korbel, Tobias Rausch, Aurélie Ernst

Last updated by author(s): May 4, 2026

Reporting Summary

Nature Portfolio wishes to improve the reproducibility of the work that we publish. This form provides structure for consistency and transparency in reporting. For further information on Nature Portfolio policies, see our Editorial Policies and the Editorial Policy Checklist.

Statistics

For all statistical analyses, confirm that the following items are present in the figure legend, table legend, main text, or Methods section.

n/a	Confirmed	
☐	☒	The exact sample size (*n*) for each experimental group/condition, given as a discrete number and unit of measurement
☐	☒	A statement on whether measurements were taken from distinct samples or whether the same sample was measured repeatedly
☐	☒	The statistical test(s) used AND whether they are one- or two-sided<br>*Only common tests should be described solely by name; describe more complex techniques in the Methods section.*
☐	☒	A description of all covariates tested
☐	☒	A description of any assumptions or corrections, such as tests of normality and adjustment for multiple comparisons
☐	☒	A full description of the statistical parameters including central tendency (e.g. means) or other basic estimates (e.g. regression coefficient) AND variation (e.g. standard deviation) or associated estimates of uncertainty (e.g. confidence intervals)
☐	☒	For null hypothesis testing, the test statistic (e.g. *F*, *t*, *r*) with confidence intervals, effect sizes, degrees of freedom and *P* value noted<br>*Give P values as exact values whenever suitable.*
☒	☐	For Bayesian analysis, information on the choice of priors and Markov chain Monte Carlo settings
☒	☐	For hierarchical and complex designs, identification of the appropriate level for tests and full reporting of outcomes
☐	☒	Estimates of effect sizes (e.g. Cohen's *d*, Pearson's *r*), indicating how they were calculated

Our web collection on statistics for biologists contains articles on many of the points above.

Software and code

Policy information about availability of computer code

Data collection	Whole-genome sequencing of cfDNA and germline DNA was performed on the Illumina NovaSeq 6000 S4 platform. Long-read tumor DNA sequencing was carried out on the PromethION (P24) device. Bulk RNA sequencing was performed on the Illumina NextSeq 2000. Visium spatial transcriptomics sequencing was conducted on the Illumina NovaSeq 6000 S1. Imaging of FFPE tissue sections stained for LINE-1 ORF1p was done using the Leica SP8 confocal microscope. All H&E, E2F3, and LINE-1 ORF1p stained sections were imaged with the Zeiss Axioscan 7.
Data analysis	BWA-MEM (0.7.15), picard tools (2.25.1), SAMtools (1.19.2), hmmcopy_utils, ichorCNA, Dorado 0.6.0, minimap2, NanoPack, Alfred, verifyBamID, Clair3, ClairS (0.4.0), WhatsHap, nf-core RNA-Seq pipeline, modkit (0.4.1), Delly (1.3.3), Severus, Sansa (0.2.3), SVAN, MELT, bedtools (2.31.1), IGV (2.18.2), BreakTracer, CNVkit (0.9.12), CoRAL, circlize (0.4.11), 10x Genomics SpaceRanger (2.0.1), Scanpy (1.11.0), CopyKAT (1.1.0), Cell2location, decoupler-py (1.9.2), SpatialDE2, TrimGalore, STAR, Salmon, DESeq2 (1.38.3), GSEA (4.4.0), nf-core RNA fusion pipeline, Arriba, ComplexHeatmap, MutationalPatterns, GenomicRanges, Pyjacker, Ensembl VEP 108, kallisto (0.51.1), VAtools, OptiType, pVACtools (5.3.0), BigMHC, DeepImmuno, MHCflurry, MHCnuggetsI, NNalign, NetMHC, NetMHCpan, NetMHCpanEL, PickPocket, SMM, dplyr (1.1.0), ggplot (2 3.5.2), tibble (3.1.8), tidyr (1.3.0), stats (4.2.2), scales (1.4.0,) DNAcopy (1.72.3), reshape2 (1.4.4), grid (4.2.2), gridExtra (2.3), cowplot (1.2.0), gzip, pandas (1.5.3), matplotlib (3.7.0), numpy (1.23.5), glob, gseapy (1.1.9), scikit-learn (1.2.1), scipy (1.10.1), seaborn (0.12.2), pysam (0.23.3), maftools (2.18.0),TxDb.Hsapiens.UCSC.hg38.refGene (3.18.0)

For manuscripts utilizing custom algorithms or software that are central to the research but not yet described in published literature, software must be made available to editors and reviewers. We strongly encourage code deposition in a community repository (e.g. GitHub). See the Nature Portfolio guidelines for submitting code & software for further information.

Data

Policy information about availability of data

All manuscripts must include a data availability statement. This statement should provide the following information, where applicable:

- Accession codes, unique identifiers, or web links for publicly available datasets
- A description of any restrictions on data availability
- For clinical datasets or third party data, please ensure that the statement adheres to our policy

Sequence data were uploaded to the German Human Genome-Phenome Archive ID GHGAS23497594176126

Research involving human participants, their data, or biological material

Policy information about studies with human participants or human data. See also policy information about sex, gender (identity/presentation), and sexual orientation and race, ethnicity and racism.

Reporting on sex and gender	We report sex of the donor as provided in the Supplementary table A of the manuscript. For all relevant analyses, the sex assigned at birth was utilized.
Reporting on race, ethnicity, or other socially relevant groupings	This study does not assess the biological variations across different ethnic and racial groups.
Population characteristics	n.a.
Recruitment	Clinical data and tissue of all patients in the study were collected after receiving written informed consent from the respective patients or their legal representatives and after approval by the ethics committee of Heidelberg University. Participants did not receive compensation and were informed of this prior to enrolment in the study.
Ethics oversight	The project was revised and approved by the ethics committee of Heidelberg University and by the BIAC committee at EMBL under the EMBL BIAC Application No. 2024-025 / 2024_HE000075.

Note that full information on the approval of the study protocol must also be provided in the manuscript.

Field-specific reporting

Please select the one below that is the best fit for your research. If you are not sure, read the appropriate sections before making your selection.

☒ Life sciences ☐ Behavioural & social sciences ☐ Ecological, evolutionary & environmental sciences

For a reference copy of the document with all sections, see nature.com/documents/nr-reporting-summary-flat.pdf

Life sciences study design

All studies must disclose on these points even when the disclosure is negative.

Sample size	Sample size was not decided based on power calculation and effect size but based on pilot experiments and preliminary findings.
Data exclusions	No samples were excluded except for 2 Visium samples not passing QC.
Replication	n.a.
Randomization	The samples were not allocated into different experimental groups.
Blinding	Comparison between experimental groups was not the purpose of this study, therefore blinding was not necessary.

Reporting for specific materials, systems and methods

We require information from authors about some types of materials, experimental systems and methods used in many studies. Here, indicate whether each material, system or method listed is relevant to your study. If you are not sure if a list item applies to your research, read the appropriate section before selecting a response.

Materials & experimental systems

n/a	Involved in the study	
☐	☒	Antibodies
☒	☐	Eukaryotic cell lines
☒	☐	Palaeontology and archaeology
☒	☐	Animals and other organisms
☐	☒	Clinical data
☒	☐	Dual use research of concern
☒	☐	Plants

Methods

n/a	Involved in the study	
☐	☒	ChIP-seq
☒	☐	Flow cytometry
☒	☐	MRI-based neuroimaging

Antibodies

Antibodies used

Primary antibodies:
Anti-LINE-1 ORF1p antibody raised in mouse (Sigma, #MABC1152, clone 4H1) was diluted 1:100.
Anti-E2F3 antibody raised in rabbit (Invitrogen, #PA5-106407) was diluted 1:50.

Secondary antibodies:
Donkey anti-mouse IgG Alexa Fluor 488 antibody (Invitrogen, #A-21202) was diluted 1:500.
Donkey anti-rabbit Alexa Fluor Plus 594 antibody (Invitrogen, #A32754) was diluted 1:300.

Validation

Both primary antibodies have been validated for IHC-P and demonstrate reactivity against human antigens according to manufacturer information and/or supporting literature:

https://www.sigmaaldrich.com/DE/en/product/mm/mabc1152?srsltid=AfmBOoop-ULf7lK2QLbU3kyfsmO86g2Qoc23m4xQs7t2GpDr35vh0JYW&icid=sharepdp-clipboard-copy-productdetailpage

https://www.thermofisher.com/antibody/product/E2F3-Antibody-Polyclonal/PA5-106407

Clinical data

Policy information about clinical studies
All manuscripts should comply with the ICMJE guidelines for publication of clinical research and a completed CONSORT checklist must be included with all submissions.

Clinical trial registration

n.a.

Study protocol

n.a.

Data collection

Collection of blood and tumor material from patients with bladder carcinoma at diagnosis was done in collaboration with Dr. Mladen Stankovic and his team at the Salem hospital (Heidelberg), who also collected the clinical data.

Outcomes

n.a.

Plants

Seed stocks

Report on the source of all seed stocks or other plant material used. If applicable, state the seed stock centre and catalogue number. If plant specimens were collected from the field, describe the collection location, date and sampling procedures.

Novel plant genotypes

Describe the methods by which all novel plant genotypes were produced. This includes those generated by transgenic approaches, gene editing, chemical/radiation-based mutagenesis and hybridization. For transgenic lines, describe the transformation method, the number of independent lines analyzed and the generation upon which experiments were performed. For gene-edited lines, describe the editor used, the endogenous sequence targeted for editing, the targeting guide RNA sequence (if applicable) and how the editor was applied.

Authentication

Describe any authentication procedures for each seed stock used or novel genotype generated. Describe any experiments used to assess the effect of a mutation and, where applicable, how potential secondary effects (e.g. second site T-DNA insertions, mosiacism, off-target gene editing) were examined.

ChIP-seq

Data deposition

☐ Confirm that both raw and final processed data have been deposited in a public database such as GEO.

☐ Confirm that you have deposited or provided access to graph files (e.g. BED files) for the called peaks.

Data access links<br>*May remain private before publication.*	We used publicly available data from ENCODE database, with the following accession numbers: ENCFF515VMS, ENCFF439WSM, ENCFF487EPL
Files in database submission	*Provide a list of all files available in the database submission.*
Genome browser session<br>(e.g. UCSC)	*Provide a link to an anonymized genome browser session for "Initial submission" and "Revised version" documents only, to enable peer review. Write "no longer applicable" for "Final submission" documents.*

Methodology

Replicates	*Describe the experimental replicates, specifying number, type and replicate agreement.*
Sequencing depth	*Describe the sequencing depth for each experiment, providing the total number of reads, uniquely mapped reads, length of reads and whether they were paired- or single-end.*
Antibodies	*Describe the antibodies used for the ChIP-seq experiments; as applicable, provide supplier name, catalog number, clone name, and lot number.*
Peak calling parameters	*Specify the command line program and parameters used for read mapping and peak calling, including the ChIP, control and index files used.*
Data quality	*Describe the methods used to ensure data quality in full detail, including how many peaks are at FDR 5% and above 5-fold enrichment.*
Software	*Describe the software used to collect and analyze the ChIP-seq data. For custom code that has been deposited into a community repository, provide accession details.*
