# Supplementary material for: Multiomic profiling links L1 retrotransposition to genomic instability and ecDNA in bladder cancer
Source: Nat Commun. 2026 Jul 16;17:6384. doi: 10.1038/s41467-026-75399-6 (PMC13376730; doi:10.1038/s41467-026-75399-6)
Supplement: Supplementary file 5 — Transparent Peer Review file [file 41467_2026_75399_MOESM5_ESM.pdf]

nature portfolio

Peer Review File

Multiomic profiling links L1 retrotransposition to genomic instability and ecDNA in bladder cancer

Corresponding Author: Dr Aurelie Ernst

This file contains all reviewer reports in order by version, followed by all author rebuttals in order by version.

Version 0:

Reviewer comments:

Reviewer #1

(Remarks to the Author)
Dear Editor,
Thank you for the opportunity to review the manuscript entitled “Integrative spatial and multi-omic profiling in bladder cancer links L1 retrotransposition to extrachromosomal DNA, genomic instability, and viral mimicry response” by Pribus et al.

The Authors showed great effort in combining a variety of approaches including integrated multi-omics and spatial analyses in a relatively large set of 49 bladder cancers.
They described genetic, epigenetic, transcriptomic, and spatial features of bladder cancer, and also added cell-free DNA blood analysis. They identified a subset of blood cancers with high LINE-1 (L1) insertions and linked them to downstream genomic rearrangements and chromosomal instability and to structural variants and extrachromosomal DNA (ecDNA). Overall the manuscript provides a large wealth of data which is interesting to the scientific community, is technically sound (including the spatial approach for ecDNA) but remains largely observational and correlative in essence. Several conclusions on the L1 activity and role in genomic instability have been described in multiple papers, although in other cancer types. While the mechanistic novelty is overall limited, this work provides valuable insights in the pathogenesis of bladder cancer.
Here are some suggestions to improve the manuscript:

- With only reads of Cutibacterium and Anaerococcus in some of the tumor sample the conclusion that microbial infection is a potential driver of L1 mobilization is too speculative. There is no validation via experiments, and the proposed mechanisms of ROS in the process is also very speculative. Thus, these conclusion shroud be tuned down and considered as speculative in the Discussion.
- Supplementary Figure 10a: There are only three examples. The authors should use the analysis like Fig. 3d, which covers promoter (n=1032), to do the comparison.
- It will be very helpful to put B60 as negative control in the Supplementary Figure 10c
- What is the X-axis mean in Supplementary Figure 10d?
- The order of Figure 3 is not linear with a, b, c, d....
- The authors should include the sequences of the two examples of translocation in Figure 3b. It seems one of them is a non-canonical L1 insertion without any polyA tail (the right one).
- The authors should indicate the name of each tumor sample in the Supplementary Figure 10h, same for Figure 3f, at least for the B42 and B60 as two positive and negative controls

Reviewer #2

(Remarks to the Author)
I co-reviewed this manuscript with one of the reviewers who provided the listed reports. This is part of the Nature Communications initiative to facilitate training in peer review and to provide appropriate recognition for Early Career Researchers who co-review manuscripts.

Reviewer #3

(Remarks to the Author)
The authors present an integrative multi-omic and spatial profiling study of 49 bladder cancer patients, combining Oxford Nanopore long-read sequencing, cfDNA low-pass sequencing, RNA-seq, and spatial transcriptomics. They report frequent LINE-1 retrotranspositions in bladder tumors, propose mechanistic links between L1 activation, extrachromosomal DNA (ecDNA) formation, and APOBEC3B/viral mimicry signaling, and highlight L1 as a potential biomarker of genomic instability and UC aggressiveness.
The study is technically ambitious. It provides a valuable long-read and spatial multi-omic dataset in UC. The connection between L1 activity, ecDNA, and innate immune response could be highly relevant to the broader understanding of cancer genome instability.
However, several major points should be addressed :

Major comments:
1. Mechanistic causality between L1 and ecDNA
The authors repeatedly implies a causal relationship between L1 retrotransposition and ecDNA formation ("L1 insertion-mediated genomic instability leading to ecDNA").
While the correlation is interesting, the evidence remains unclear.
- No examples show L1 fragments directly within ecDNA boundaries.
- The linear correlation between L1 count and ecDNA presence could also reflect shared underlying genomic instability rather than causation.

Clarify that the data are correlative and explicitly state that causality is hypothesized but not demonstrated. Consider including a schematic summarizing plausible but unproven models.

2. Quantitative validation of L1 detection
The detection of >500 somatic L1 insertions in a single tumor is striking and beyond what has been reported in other cancer types.
- How was the false discovery rate estimated?
- Are all insertions supported by unique long reads, or could this include chimeric/mis-mapped reads?
- Validation by orthogonal methods (PCR, short-read confirmation, or visual inspection) is limited.

The authors should provide per-sample QC metrics (read coverage, insertion support, validation rate) and discuss potential overestimation due to Nanopore artifacts.

3. Spatial transcriptomics interpretation
The approach to infer ecDNA from 10x Visium data is intriguing but methodologically unclear. Can you clarify?:
- How are ecDNA-high clusters defined?
- Is there potential confounding by tumor purity or local copy-number variation?
- APOBEC3B enrichment could reflect immune infiltrates rather than ecDNA presence.

Include a clearer methodological flowchart and validation on at least one additional sample with orthogonal (FISH or DNA FISH) spatial validation.

4. The manuscript links L1 activity to activation of cGAS-STING and RIG-I/MDA5 pathways.
However, most p-values are marginal (n = 12 RNA-seq samples).
Tone down claims ("suggests" instead of "demonstrates") and discuss alternative explanations (e.g., chronic inflammation, bacterial contamination, or APOBEC-driven dsDNA release unrelated to L1).

5. The section on Anaerococcus and Cutibacterium looks like very speculative and lacks functional validation.
Given the small number of positive samples and the high risk of reagent contamination, this add confusion for the main message.
Move this to supplementary data and clearly state that the evidence for bacterial contribution is anecdotal.

6. While the biological insights are rich, the clinical dimension is not clearly developed.
- Were L1-high tumors associated with clinical variables (stage, grade, recurrence, or survival)?
- Can L1 count in cfDNA serve as a biomarker? Adding even exploratory analyses would strengthen translational relevance.

7. Manuscritpt structure
The manuscript is extremely dense and could be more reader-friendly.
- The two first sections of the introduction should be merged.
- The Results section mixes discovery, validation, and speculation.
- Consider a clearer structure. I would suggest:
1- Multi-omic landscape
2- L1 discovery and validation
3- L1–ecDNA relationship
4- Viral mimicry and immune contexture
5- Integrative model

Minor comments:

- Figures 2–4 should be improved; axes and legends are often too small.
- Provide accession numbers for sequencing data
- Clarify in the abstract that causation between L1 and ecDNA is hypothesized.
- In the Introduction, include reference to bladder cancer-specific genomic instability studies (e.g., Robertson et al., Cell 2017; Kamoun et al., Eur Urol 2020).
- Methods section should mention quality control and filtering criteria for L1 and ecDNA calls.
- Typo note: “Long interspersed element 1” should be consistently abbreviated as LINE-1 (L1), not alternately L1 or LINE1.

Reviewer #4

(Remarks to the Author)
This is a well-organized manuscript in which the authors resolve bladder cancer using multi-omics approaches. Short-read WGS, long-read WGS, bulk and spatial transcriptomics were integrated to profile the somatic variants and dissect the tumor microenvironment. However, here are some major concerns.

Major:
1. Study design: Are benign or normal tissues available in the tissue bank? The study has been focusing on the tumor tissue and the blood samples, while it’s unclear if the differences are caused by tumor or tissue. If benign, normal or paired bladder tissues can be measured in the same way (long-read WGS, bulk or spatial transcriptomics), the control group will be clearer.
2. Please provide more QC metrics for different omics measurements. For example, only the mean coverage across all the samples was provided, while notfor individual samples. Will the sequencing depth (or accuracy, quality, GC content, etc) influence the CNV and mutation detection? For example, the library with low coverage by long-read WGS will yield a smaller number of variants.
3. The ONT technology has been reported to reach lower sequencing accuracy when compared with short-read sequencing. How did the authors specifically address this concern when calling mutations from long-read data. How to control the false positives? In the method section, it’s unclear how the top or high-confident variants were screened out.
4. Have the SVs, SNVs and gene fusions been fully or partially validated by experimental molecular approaches, such as PCR, Sanger sequencing, etc? If not, I would highly suggest that the authors choose the top variants in each category to validate. For example, the 2 fusion in Supp Figure 1, the 12 high-impact variants the ones listed in Figure 1b.
5. Please provide the accession ID of the omics data in the ‘Data Availability’ section. The authors can keep the data in private status until the manuscript acceptance/ publication.
6. Microbial infection analysis: other than ONT data, bulk and spatial transcriptomics data can also detect and estimate microbial (bacteria and virus) expression. Given the multi-omics scope of this project, I would highlighly recommend the authors perform comprehensive microbial infection analysis using these omics datasets. They can also validate the finding from the ONT data.
7. Figure 4: Spatial clustering was performed, while the biological interpretation of the clusters are not clear. The authors only focus on cluster 0 and 1, but not the others. In addition, 10 spatial transcriptomics were performed. Can their clusters be matched together? Any difference among their clinical features, such as the low vs high grade, MIBC vs NMIBC, tumor number and size, or age?
8. Figure 4: The transcriptome and multi-omics level analyses are not sufficient. For example, given the spatial Visium and bulk RNA-seq data, the authors can perform cell annotation (Figure 4c has clusters but no cell type annotation), cell type deconvolution, cellular interaction, and trajectory inference. The authors can also integrate the variant calling with the expression level analysis to resolve bladder cancer in a systematic manner.
9. This manuscript focuses on the L1 elements. It couldn’t be better to show the overall variant distribution, such as 5’ UTR, intron, exon, 3’ UTR, or other transposable elements, such as SINE or LTR.
10. Please discuss the limitations and future works of this project in the Discussion section.

Minor:
1. Figure 2B: Please highlight the MIBC and NMIBC in the top bar. Do they show differential patterns in SCNA and mutation patterns?
2. Figure 4c: Why is cluster 8 not available?
3. Figure 4h and 4k: What do ‘false’ and ‘true’ represent? All the bars are green (true).
4. Supplementary Figure 2: I would suggest the authors align the tumor long-read WGS with the cfDNA short-read WGS for the same patient, as it was shown in Fig 2a-b. Otherwise, there is no control for the germline variant calling. In addition, coloring the variant positions with different backgrounds will help highlight the variants.

Version 1:

Reviewer comments:

Reviewer #1

(Remarks to the Author)
Dear Editor,
we reviewed the revised manuscript. The Authors addressed satisfactorily all the criticisms raised by our side.
The manuscript is markedly improved and we do not have further comments.

(Remarks on code availability)

Reviewer #2

(Remarks to the Author)
I co-reviewed this manuscript with one of the reviewers who provided the listed reports. This is part of the Nature Communications initiative to facilitate training in peer review and to provide appropriate recognition for Early Career Researchers who co-review manuscripts.

(Remarks on code availability)
I have send my comments to Roberto Chiarle, and he will submit all in one.

Reviewer #3

(Remarks to the Author)
The authors provided extensive responses to my concerns and comments.

(Remarks on code availability)

Reviewer #4

(Remarks to the Author)
I would appreciate the great manuscript revision work! The authors have addressed all my concerns.

(Remarks on code availability)

Reviewer #5

(Remarks to the Author)
In the manuscript entitled “Integrative spatial and multi-omic profiling in bladder cancer links L1 retrotransposition to extrachromosomal DNA, genomic instability, and viral mimicry response”, Pribus et al profiled samples from 48 bladder cancer patients combining long-read sequencing, spatial transcriptomics, and other genomic approaches. They identified frequent L1 insertions as an early event of bladder cancer, which potentially drives subsequent genome instability, such as APOBEC3B-mediated mutagenesis and ecDNA formation, further promoting tumorigenesis. L1 activation has also been linked to downstream viral-mimicry effects. Overall, this is a comprehensive study using multi-omics to study genome instability in bladder cancer progression. Despite being somewhat descriptive, the study provides rich genomic resources and infers how genomic instability events are linked to bladder cancer progression. Following the Editor's instructions, my assessment primarily focused on ecDNA.

1. In supplementary figure 4e, the authors mentioned that most of the tumor-promoting genes activated through enhancer hijacking were independent of ecDNA formation. This is true and unsurprising because ecDNAs comprise only a small fraction of the cancer genome. However, according to this figure panel, 13 of the 21 ecDNA genes were involved in enhancer hijacking, if I read it correctly. Therefore, the message that ecDNA is a hotspot for enhancer hijacking should not be overlooked (e.g. PMID: 33199677). If readers do not carefully digest the original sentence, they may misunderstand the message. This is a minor point but needs to be revised.

2. In supplementary figure 14f, the authors claimed that higher L1 insertion is positively associated with the presence of ecDNAs. However, the P value was 0.0538 even under the one-sided test. I understand the challenge of sample size limitation that reduces statistical power. However, we should still be stringent. The sentence should be rephrased to show the trend by ecDNA stratification while acknowledging the insignificance due to limited power.
The same problem appears in Supplementary figures 14g-h. In 14g, the coefficient was indeed higher in the ecDNA+ cohort than in the ecDNA- cohort. However, whether this difference is significant remains unclear. The 14h panel addressed this question. However, it was insignificant even by the one-sided test. Again, I understand the challenge of a limited sample size. However, we should not send the wrong message to the field.
For 14h, another possible explanation for the enrichment of active L1 loci expression in ecDNA-positive patients is that some L1 elements may reside on ecDNAs and become transcriptionally activated due to the permissive chromatin environment associated with ecDNA. In this scenario, L1 activation would be a consequence rather than a driver of ecDNA formation. Notably, recent reports (PMID: 39282372) have suggested that retroelements located on ecDNA can be transcriptionally active. This possibility should be considered and at least discussed.
Therefore, all L1-ecDNA-related texts should be carefully revised, including in the abstract, which emphasizes the intimate relationship between L1 and ecDNA.

3. I find it difficult to understand the “sample-specific ecDNA signature” method. To the best of my understanding, was the rationale as below?
Spatial transcriptomic signals were sparse, limiting the sensitivity of ecDNA cluster detection; therefore, the authors

extracted gene expression signatures from high-confidence ecDNA clusters, using ecDNA gene-high cells as a surrogate to infer cells that may contain ecDNAs. This method was applied on a case-by-case basis. For example, the B4 signature was only used in the B4 sample, but not in other samples.
Alternatively, were the aggregated gene signatures from 3 samples (B4, B42, and B123) applied to all samples with spatial data?
The authors must explain it clearly to the readers. And if the method was applied in the second way I described above, the authors must present careful benchmark data to demonstrate sensitivity and specificity, using a series of ground-truth positive and negative samples, other than the "training set," as references.

4. In Figure 5i and also in the main text, the authors proposed that APOBEC3B-mediated mutagenesis promotes ecDNA formation: "These findings support a model in which EMT-associated chromatin relaxation permits elevated L1 transcription, leading to increased DNA damage and APOBEC3B activation, thereby promoting genomic fragmentation and circularization events that underlie ecDNA formation." In a previous report studying the APOBEC3B mutagenesis and ecDNA evolution (PMID: 35140399, also referred by the authors), it proposes that most of the APOBEC3B-mediated katagies mutational events likely occurred after ecDNA formation and clonal amplification, which likely serves as a mechanism to drive ecDNA evolution, rather than formation. In this manuscript, the authors identified APOBEC3B activity upregulation (potentially caused by L1 activation) as an early event, potentially driving ecDNA formation. The differences between this and previous discoveries could be cancer-type specific or due to sampling biases. Alternatively, this might be a novel mechanism specific to L1-mediated ecDNA formation. The authors do discuss their hypothesis in the discussion section, but the conclusions from the previous studies suggesting APOBEC is unlikely to be involved in ecDNA formation are not fully emphasized.

(Remarks on code availability)

Reviewer #6

(Remarks to the Author)
I co-reviewed this manuscript with one of the reviewers who provided the listed reports. This is part of the Nature Communications initiative to facilitate training in peer review and to provide appropriate recognition for Early Career Researchers who co-review manuscripts.

(Remarks on code availability)

Open Access This Peer Review File is licensed under a Creative Commons Attribution 4.0 International License, which permits use, sharing, adaptation, distribution and reproduction in any medium or format, as long as you give appropriate credit to the original author(s) and the source, provide a link to the Creative Commons license, and indicate if changes were made.
In cases where reviewers are anonymous, credit should be given to 'Anonymous Referee' and the source.
The images or other third party material in this Peer Review File are included in the article's Creative Commons license,

unless indicated otherwise in a credit line to the material. If material is not included in the article's Creative Commons license and your intended use is not permitted by statutory regulation or exceeds the permitted use, you will need to obtain permission directly from the copyright holder.
To view a copy of this license, visit https://creativecommons.org/licenses/by/4.0/

REVIEWER COMMENTS

Reviewer #1 (Remarks to the Author):

Dear Editor,
Thank you for the opportunity to review the manuscript entitled "Integrative spatial and multi-omic profiling in bladder cancer links L1 retrotransposition to extrachromosomal DNA, genomic instability, and viral mimicry response" by Pribus et al.

The Authors showed great effort in combining a variety of approaches including integrated multi-omics and spatial analyses in a relatively large set of 49 bladder cancers.
They described genetic, epigenetic, transcriptomic, and spatial features of bladder cancer, and also added cell-free DNA blood analysis. They identified a subset of blood cancers with high LINE-1 (L1) insertions and linked them to downstream genomic rearrangements and chromosomal instability and to structural variants and extrachromosomal DNA (ecDNA).
Overall the manuscript provides a large wealth of data which is interesting to the scientific community, is technically sound (including the spatial approach for ecDNA) but remains largely observational and correlative in essence. Several conclusions on the L1 activity and role in genomic instability have been described in multiple papers, although in other cancer types. While the mechanistic novelty is overall limited, this work provides valuable insights in the pathogenesis of bladder cancer.
Here are some suggestions to improve the manuscript:

Reviewer #1–1: With only reads of Cutibacterium and Anaerococcus in some of the tumor sample the conclusion that microbial infection is a potential driver of L1 mobilization is too speculative. There is no validation via experiments, and the proposed mechanisms of ROS in the process is also very speculative. Thus, these conclusions should be tuned down and considered as speculative in the Discussion.
Response: As suggested, we have now toned down the conclusion on the potential role of microbial infection and revised the text accordingly (page 6): "Hence, microbial infection does not seem to be a frequent driver of L1 mobilization".
In light of recent evidence from Sheng et al. (2025, PMID: 40696353), which shows that Anaerococcus - among other genera - is enriched in the urine of bladder cancer patients compared to controls, we still mention the possibility of a putative role in the revised discussion.

Related comments by other reviewers (comment #3–5 and #4-6) also prompted us to conduct further in-depth analysis of the microbial content using additional methods and data modalities. First, we complemented our previous Kraken2 k-mer based microbial analysis workflow with a sequence alignment-based analysis pipeline from the GATK toolkit (PathSeq). Analysis of the ONT data from B42 revealed a similar microbial abundance estimate for Anaerococcus: 45.7% with PathSeq compared to 54.9% with Kraken2. For Cutibacterium, PathSeq labelled more reads as ambiguous alignments which lead to overall lower abundance estimates for Cutibacterium in all samples (range 0% - 7.9% with PathSeq).
As suggested by reviewer 4 (comment #4-6), we also used the bulk transcriptomic data to confirm the presence of Anaerococcus reads in B42. As microbial mRNA is short-lived and less common than human mRNA, we could not directly compare microbial abundances between DNA and RNA-Seq data. Using Kraken2, 6,890 RNA-Seq reads of B42 (1.56% of all unmapped reads) were classified as Anaerococcus, underscoring the presence of this genus in both DNA and RNA-Seq data for B42. The most abundant genus in the RNA-Seq data was Bradyrhizobium (range 0.1% - 47.9%), which is another microbial species found to be enriched in recurrent bladder cancer by Sheng et al. (2025).
These new results supporting the pathogen analysis are now presented in the revised Supplementary Figure 11.

Reviewer #1–2: Supplementary Figure 10a: There are only three examples. The authors should use the analysis like Fig. 3d, which covers promoter (n=1032), to do the comparison.
Response: As suggested, we have now performed a new analysis like the analysis presented in Fig. 3d which covers the promoter (n=2,631 CpG sites) and the body (n=2,631 CpG sites) instead of showing representative examples. These new data are presented in the revised Supplementary Figure 12a.

Reviewer #1–3: It will be very helpful to put B60 as negative control in the Supplementary Figure 10c

Response:
Thank you for this suggestion. We would like to clarify the analysis that we performed in Supplementary Figure 12c (formerly 10c). The graph reflects the methylation of the full length somatic L1 in the sample B42. Since somatic L1 insertions are unique to an individual and different from germline insertions that are shared on a population level, we believe that the comparison in methylation of somatic L1 between patients would be inaccurate. We would like to point out that we have performed a methylation comparison between B60 and B42

germline L1 that come from the same loci; these results are shown in revised Supplementary figure 12a.

Reviewer #1–4: What is the X-axis mean in Supplementary Figure 10d?
Response: Supplementary Figure 12d (formerly 10d) shows a rainfall plot displaying intermutation distance (y-axis) as a function of the sequential mutation rank (x-axis). The L1 insertion rank indicates the relative position of the L1 insertions along the genome and the y-axis indicates the distance to the previous L1 insertion. Clustered L1s would thus appear as rainfalls in the plot, similar to clustered SNV mutations due to kataegis. We have now clarified this in the revised legend of Supplementary Figure 12d.

Reviewer #1–5: The order of Figure 3 is not linear with a, b, c, d....
Response: We have now adjusted the order of the references to the figures in the main text. The figure panels are now cited in the correct order from a to h in the revised text.

Reviewer #1–6: The authors should include the sequences of the two examples of translocation in Figure 3b. It seems one of them is a non-canonical L1 insertion without any polyA tail (the right one).
Response: Thank you for this suggestion. We locally assembled each event from the supporting reads and as the reviewer noted, only one of them has a poly-A tail. Intriguingly, the L1 coordinates suggest that both L1 fragments embedded in the two translocations are potentially derived from a single source somatic L1, which would naturally lead to one L1 fragment lacking a poly-A tail and the other L1 fragment keeping the poly-A tail.
To further illustrate this L1-mediated rearrangement in more detail, we show in the new Supplementary Figure 13 the alignment of breakpoint-supporting reads to the local assembly of each inter-chromosomal translocation.

Reviewer #1–7: The authors should indicate the name of each tumor sample in the Supplementary Figure 10h, same for Figure 3f, at least for the B42 and B60 as two positive and negative controls
Response: As suggested, we have now indicated the name of each tumor sample in the revised Supplementary Figure 10h (now labelled Supplementary Figure 14a) and the revised Figure 3f.

Reviewer #2 (Remarks to the Author):

I co-reviewed this manuscript with one of the reviewers who provided the listed reports. This is part of the Nature Communications initiative to facilitate training

in peer review and to provide appropriate recognition for Early Career Researchers who co-review manuscripts.
Response: Thank you for co-reviewing the manuscript.

Reviewer #3 (Remarks to the Author):

The authors present an integrative multi-omic and spatial profiling study of 49 bladder cancer patients, combining Oxford Nanopore long-read sequencing, cfDNA low-pass sequencing, RNA-seq, and spatial transcriptomics. They report frequent LINE-1 retrotranspositions in bladder tumors, propose mechanistic links between L1 activation, extrachromosomal DNA (ecDNA) formation, and APOBEC3B/viral mimicry signaling, and highlight L1 as a potential biomarker of genomic instability and UC aggressiveness.
The study is technically ambitious. It provides a valuable long-read and spatial multi-omic dataset in UC. The connection between L1 activity, ecDNA, and innate immune response could be highly relevant to the broader understanding of cancer genome instability.
However, several major points should be addressed :

Major comments:

Reviewer #3–1: Mechanistic causality between L1 and ecDNA
The authors repeatedly implies a causal relationship between L1 retrotransposition and ecDNA formation ("L1 insertion-mediated genomic instability leading to ecDNA").
While the correlation is interesting, the evidence remains unclear.
- No examples show L1 fragments directly within ecDNA boundaries.
- The linear correlation between L1 count and ecDNA presence could also reflect shared underlying genomic instability rather than causation.

Clarify that the data are correlative and explicitly state that causality is hypothesized but not demonstrated. Consider including a schematic summarizing plausible but unproven models.

Response: Thank you for this comment highlighting that the correlation between somatic L1 activity and ecDNA formation that we report warrants more in-depth investigation. In order to address this reviewer question, we performed three types of analyses – using our own long-read sequencing data as well as two external cohorts, namely TCGA (n= 112 bladder cancer patients with matched L1 and ecDNA data) and PCAWG (n= 2,642 patients from 38 tumor types). These new results substantially strengthen our findings on the link between L1 and ecDNA formation, as outlined below:

We first evaluated the association between L1 count and ecDNA in additional datasets. We reanalyzed TCGA cohort data from two published papers on bladder cancer on the same samples, one providing sample-level L1 expression data (Solovyov et al 2025, PMID: 40021663), and one providing sample-level ecDNA status (Hung et al 2024, PMID: 39506152). We identified a significant positive correlation between patient amplicon count and L1 retrotranscriptional burden, a correlation that was stronger in ecDNA-positive patients (ecDNA+: $\rho = 0.63$, $p = 2.59e-4$, ecDNA-: $\rho = 0.34$, $p = 6.88e-3$). Active L1 loci expression was also elevated in ecDNA-positive patients, even though this difference was not significant with this cohort size ($p = 0.087$). These results have been added as new Supplementary Figures 14g-h.
While we acknowledge that L1-insertions must not necessarily lead to ecDNA formation, our results suggest a non-random correlation. Importantly, our data are in line with recent *in vitro* studies. Notably, in cultured cells, ecDNA has been shown to be enriched for transposable element sequences including L1 fragments, and these retrotransposon-associated elements can be actively transcribed from ecDNA and function as enhancers in oncogenic contexts (PMID: 41120733). Hence, we further explored downstream effects of L1 insertions to better understand how they may be related mechanistically to ecDNA formation and other genomic features.

We found that L1 promoters were significantly demethylated (Fig. 3b), supporting the idea that they were active. We now confirm this activity using the L1EM tool to show that samples harboring extensive somatic L1 insertions also display pronounced transcriptional activation of L1 elements (new Supplementary Fig. 14b-c). Having confirmed their activity, we moved on to mechanistic explorations.

We first focused on the potential role of somatic L1s in SV generation. At the genomic level, we further refined our BreakTracer algorithm to comprehensively detect complex SVs containing somatic L1 fragments in our own ONT data. This analysis revealed a multi-layered panorama of diverse rearrangements involving L1 sequences (Supplementary Fig. 12 and 13). In addition to inter-chromosomal SVs with L1 sequences at the breakpoints, we identified further genomic rearrangement classes incorporating L1 fragments, including templated insertions, deletions, and tandem duplications (new Supplementary Fig. 13). Although comprehensive characterization of these rearrangements across tumor types will likely require larger cohorts of long-read sequenced cancer genomes, we initiated this effort by revisiting the short-read PCAWG data: We now show that somatic L1 insertions are enriched near copy-number alteration breakpoints that cannot be explained by a coincident somatic SV compared with copy-number breakpoints that do have a matching SV (Fig. 3i). This finding suggests that somatic L1

fragments located between CNV breakpoints impede short-read sequencing from spanning complex rearrangements (Supplementary Fig. 14e). This interpretation is consistent with observations by Choo et al. (Nature Genetics, 2023), who demonstrated that viral integrations at SV junctions can also give rise to CNV breakpoints that are unexplained by SVs in short-read data. By extending this observation to somatic L1 elements located near copy-number shifts in the tumor read-depth signals across multiple histologies in PCAWG (Supplementary Fig. 14j), our results highlight the need for a more systematic investigation of how somatic L1 activity shapes the SV landscape across diverse cancer types.

We had also shown that L1-high samples display a significant enrichment of the hallmark epithelial-to-mesenchymal transition (EMT) program (Fig. 5e), a cellular state known to involve extensive chromatin remodeling, replication stress, and increased tolerance of genomic instability. This is in agreement with a previous study positively correlating EMT signaling with L1 ORF1p expression (PMID: 36707636). In this context, we performed a new analysis during manuscript revision of bulk RNAseq data in our bladder cancer samples. We found an up-regulation of gene sets associated with H3K9 heterochromatin formation and transposable element silencing, including elevated expression of the H3K9 methyltransferases *SUV39H1*, *SUV39H2* and *MORC2*, consistent with engagement of compensatory epigenetic defense mechanisms in response to increased retrotransposon activity (Fig. 5g, Supplementary Fig. 20). Despite this response, canonical heterochromatin-repressed loci, most notably MAGE family cancer–testis antigens, are derepressed in L1-high samples (Supplementary Fig. 20c, Supplementary Fig. 20f), indicating functional insufficiency of H3K9-mediated silencing. Importantly, it has previously been shown that MAGE expression is associated with tumor aggressiveness, which we hypothesize may be due either to the relationship with L1 expression or a mutual enrichment of both MAGE and L1 in high grade tumors (PMID: 35022469). We also previously detected increased expression of *APOBEC3B*, a cytidine deaminase induced by DNA damage and replication stress and implicated in the mutational diversification of extrachromosomal DNA (ecDNA). We further note trends toward increased expression of *AGO1* and *DICER1* (Supplementary Fig. 20c), suggesting partial engagement of RNA-mediated surveillance pathways in response to abundant repeat and retrotransposon transcripts.

Hence, our analyses support a strong link between L1 insertions and SVs as well as elevated L1 transcription promoted by EMT-associated chromatin relaxation. Both processes lead to increased DNA damage, with the later further contributing to APOBEC3B activation, thereby promoting genomic fragmentation and circularization events that underlie ecDNA formation. The simultaneous induction, but apparent functional inadequacy of multiple epigenetic and RNA-based

silencing mechanisms (as previously documented in L1 RNA activity (PMID: 35947677), highlights a state of persistent genomic stress linking L1 activity, epigenetic dysregulation, and ecDNA biology.

To clarify our findings and proposed hypotheses, we have made a graphical abstract/schematic representation of our results (new Fig. 5i), as suggested by the reviewer. This schematic depicts both demonstrated and hypothesized connections between L1 insertions and multiple forms of genomic instability, including epigenetic dysregulation, structural variation, extrachromosomal DNA (ecDNA), and viral mimicry responses.

Reviewer #3–2: Quantitative validation of L1 detection
The detection of >500 somatic L1 insertions in a single tumor is striking and beyond what has been reported in other cancer types.
- How was the false discovery rate estimated?
- Are all insertions supported by unique long reads, or could this include chimeric/mis-mapped reads?
- Validation by orthogonal methods (PCR, short-read confirmation, or visual inspection) is limited.

Response: We agree with the reviewer that it is important to validate the high number of somatic L1 insertions observed in a subset of tumors and to estimate the false discovery rate (FDR).
In the initial manuscript, we already applied a consensus approach using Severus and Delly. Such ensemble strategies have previously been shown to improve specificity by reducing false positive SV predictions (e.g., Zarate et al., GigaScience, 2020). In addition, we employed a panel-of-normals (PON) approach to filter likely germline variants, using data from Schloissnig et al. (Nature, 2025) comprising 1,019 ONT-sequenced samples from the 1000 Genomes cohort. As PON-based filtering can miss rare and ultra-rare polymorphic variants, we have now further improved this procedure by implanting all called insertions, including L1 events, into the GRCh38 reference and remapping the short-read germline data to this augmented reference. We subsequently filtered out candidate somatic insertions that exhibited at least 50% of the genome-wide median coverage in the matched control. We note that while this filtering step effectively removes remaining germline artifacts, it may be overly stringent and favors specificity over sensitivity, as short reads may be incorrectly mapped or may fail to map in repetitive contexts such as L1 sequences. For B42, the sample with the highest number of previously reported somatic L1 insertions (n = 524), this stringent filtering retains 490 L1 insertions as somatic (93.5%).
To estimate the false discovery rate (FDR) of the somatic L1 insertions, we generated additional short-read whole-genome sequencing data from the tumor

tissue of patients B42 and B125 for orthogonal validations, as suggested by the reviewer. We note that short-read sequencing has inherent limitations for detecting somatic L1 insertions, as (i) sufficient unique flanking sequence is required to anchor reads unambiguously to the reference genome, and (ii) reads must span the combined length of the target site duplication (mean ~15 bp) and the poly(A) tail (mean ~45 bp) to reach into the diagnostic L1 3′ UTR. In addition, short-read approaches may fail to validate insertions located in repetitive genomic contexts. We first analyzed the short-read tumor data using MELT, a commonly used short-read method to call mobile element insertions, to identify putative L1 insertions present in tumor tissue but absent from the matched blood sample. This analysis confirmed only 111 of the 490 somatic L1 insertions and thus, sensitivity was substantially reduced compared with long-read based SV detection as noted previously (e.g., Kosugi et al., Human Genome Variation, 2024). We next used our strategy of implanting insertions into the GRCh38 reference to mitigate this detection bias. We therefore applied this procedure to the tumor tissue as well and assessed whether the normalized coverage (corrected for overall sequencing depth) was higher in the tumor than in the matched control sample for each somatic L1 insertion. This criterion was met for 469 of the 490 somatic L1 insertions in B42, corresponding to an estimated FDR of 4.3%. For all somatic insertions called in B42, the estimated FDR was slightly higher (8.3%), confirming previous observations that mobile element insertions exhibit lower FDRs compared to all insertions due to their well-defined allele architecture (Schloissnig et al., Nature, 2025).

The authors should provide per-sample QC metrics (read coverage, insertion support, validation rate) and discuss potential overestimation due to Nanopore artifacts.
Response: As requested, we now provide per-sample quality control metrics for the L1 insertions, including read coverage, read support for the L1 insertion, length of the retrotransposon, strand and structural variant call quality (Supplementary Table L). We discuss potential overestimation due to the error rate of Nanopore sequencing in the revised discussion (page 13, “Limitations and future work”).

Reviewer #3–3: Spatial transcriptomics interpretation
The approach to infer ecDNA from 10x Visium data is intriguing but methodologically unclear. Can you clarify?:
- How are ecDNA-high clusters defined?
We have now added more details to the revised Methods to describe how the ecDNA enrichment across clusters is defined (see section “*Identification of ecDNA-enriched regions in Visium data”,* page 22).

- Is there potential confounding by tumor purity or local copy-number variation?
As we regress out the CNV score (see Methods), which is a reliable proxy for tumor purity, potential confounding by tumor purity is minimized. We have now added this information to the revised caption of Figure 4 to clarify this point.

If by "local copy-number variation" the reviewer refers to spatially restricted chromosomal amplifications, these could indeed potentially influence transcription-based estimates of ecDNA enrichment. We addressed this by deriving ecDNA-specific gene signatures from ONT-reconstructed ecDNA structures. However, we acknowledge that transcriptional changes driven by focal chromosomal amplifications cannot be fully distinguished from ecDNA-driven transcriptional signals using probe-based, spot-level Visium FFPE data alone. We added this point to the limitations of our study (see revised discussion).

- APOBEC3B enrichment could reflect immune infiltrates rather than ecDNA presence.
As a clarification, we do not claim that *APOBEC3B* enrichment could reflect ecDNA presence, this might have been a misunderstanding.

We agree with the reviewer that the potential contribution of immune cells to transcriptional signatures is important to evaluate. To test if *APOBEC3B* expression was driven by immune infiltration, we fit a multiple linear regression model to log-normalized *APOBEC3B* expression per Visium spot, with standardized cell2location-inferred cell type abundances as predictors. The adjusted R^2 of the model was 0.43. The strongest positive association was observed for the basal epithelial subtype (standardized β = 0.1487). Immune cell types showed no significant association with *APOBEC3B* expression, as shown below.

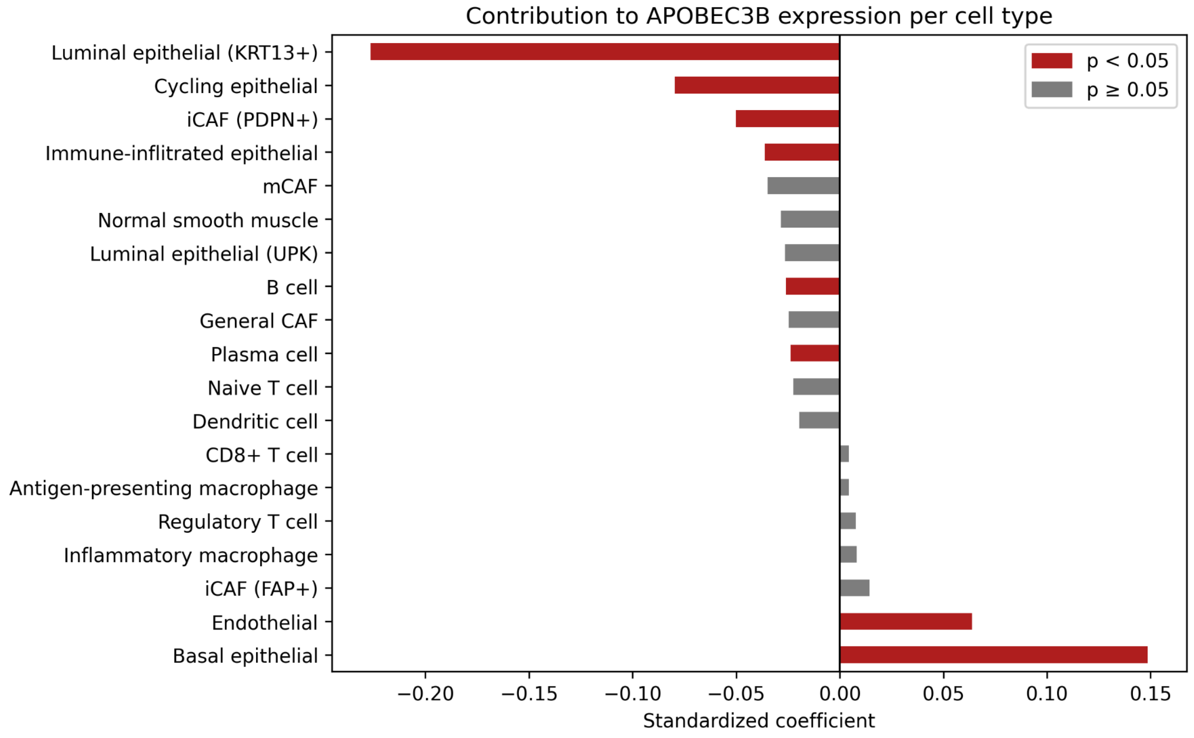

Figure R 3-3: Standardized coefficients from a multiple linear regression model of log-normalized *APOBEC3B* expressions per spot. Bars represent effect sizes (**β**) and are colored by significance ($p < 0.05$).

Include a clearer methodological flowchart and validation on at least one additional sample with orthogonal (FISH or DNA FISH) spatial validation.
As suggested, we have generated a methodological flowchart that describes our new ecDNA detection method from Visium data (new Supplementary Figure 16f).

In the initial version of the manuscript, we had included DNA FISH validations for two patients, namely B42 and B123. We had originally split the tissues into different regions for which the FISH signals were quantified separately to confirm the Visium data (Supplementary Figure 9).

We have now performed FISH on two additional samples, namely B4, which shows ecDNAs containing *MDM2*, and B96 as a diploid control. We did an automated quantification of the FISH signals in space on consecutive sections from the slides used for the Visium analysis. This analysis showed that the control sample B96 is diploid for *MDM2*, as expected (revised Figure 4f). In patient B4, the areas predicted as ecDNA-high showed extrachromosomal amplifications of *MDM2* by FISH as well (revised Figure 4d-g, Supplementary Figure 16).
Hence, this orthogonal spatial validation by FISH confirms the Visium-based ecDNA predictions.

Reviewer #3–4: The manuscript links L1 activity to activation of cGAS-STING and

RIG-I/MDA5 pathways.
However, most p-values are marginal (n = 12 RNA-seq samples).
Tone down claims (“suggests” instead of “demonstrates”) and discuss alternative explanations (e.g., chronic inflammation, bacterial contamination, or APOBEC-driven dsDNA release unrelated to L1).

As suggested, we have now toned down the claims related to the links between L1 activity and cGAC-STING and RIG-I/MDA5 in the abstract (“support” instead of “offer evidence for”), the revised results section (“suggest” instead of “support”, (see revised text page 10, second paragraph).

We have also added alternative explanations including chronic inflammation, bacterial contamination, or APOBEC-driven dsDNA release unrelated to L1 to the revised discussion (see page 13, “Limitations and future work”).

Reviewer #3–5: The section on Anaerococcus and Cutibacterium looks like very speculative and lacks functional validation.
Given the small number of positive samples and the high risk of reagent contamination, this add confusion for the main message.
Move this to supplementary data and clearly state that the evidence for bacterial contribution is anecdotal.
As suggested, we have now moved the section on Anaerococcus and Cutibacterium to the Supplementary data (revised Supplementary Figure 11) and rephrased the text to reflect the fact that the evidence for bacterial contribution is limited.
However, as mentioned in the response to point 1 from reviewer 1, in light of recent evidence from Sheng et al. (2025), which shows that Anaerococcus - among other genera - is enriched in the urine of bladder cancer patients compared to controls, we still mention the possibility of a putative role in the discussion.
Similar comments by other reviewers (comment #1–1 and #4-6) also prompted us to conduct further in-depth analysis of the microbial content using additional methods and data modalities. First, we complemented our previous Kraken2 k-mer based microbial analysis workflow with a sequence alignment-based analysis pipeline from the GATK toolkit (PathSeq). Analysis of the ONT data from B42 revealed a similar microbial abundance estimate for Anaerococcus: 45.7% with PathSeq compared to 54.9% with Kraken2. For Cutibacterium, PathSeq labelled more reads as ambiguous alignments which lead to overall lower abundance estimates for Cutibacterium in all samples (range 0% - 7.9% with PathSeq).
As suggested by reviewer 4 (comment #4-6), we also used the bulk transcriptomic data to confirm the presence of Anaerococcus reads in B42. As microbial mRNA is

short-lived and less common than human mRNA we could not directly compare microbial abundances between DNA and RNA-Seq data. Using Kraken2, 6,890 RNA-Seq reads of B42 (1.56% of all unmapped reads) were classified as Anaerococcus, underscoring the presence of this genus in both DNA and RNA-Seq data for B42. The most abundant genus in the RNA-Seq data was Bradyrhizobium (range 0.1% - 47.9%), which is another microbial species found to be enriched by Sheng et al. (2025) in recurrent bladder cancer.
These new results related to the pathogen analysis are now presented in the revised Supplementary Figure 11.

Reviewer #3–6: While the biological insights are rich, the clinical dimension is not clearly developed.
- Were L1-high tumors associated with clinical variables (stage, grade, recurrence, or survival)?

Response: As suggested, we have tested for associations between L1 counts and clinical variables. Survival data were not available unfortunately, but we tested for associations with grade, stage, age, sex and smoking status. We showed a significant association of L1-high tumors with higher tumor grade (Mann Whitney, p=0.009). These results are now presented in revised Figure 1B and Supplementary Figure 8a-e.

- Can L1 count in cfDNA serve as a biomarker? Adding even exploratory analyses would strengthen translational relevance.
Response:
To search for somatic L1 insertions in the cfDNA, we applied the following strategy. First, using the somatic L1 insertions and their surrounding genomic sequences assembled from the ONT data, we constructed personalized genome references for each patient. We then mapped cfDNA sequencing reads for each patient to these personalized references. To ensure confident detection of L1 insertions, we restricted our analysis to high-quality reads that unambiguously aligned to the L1 junctions. At least one high-quality junction-spanning alignment was required to consider an insertion as detected in the cfDNA. Despite the relatively low cfDNA sequencing depth (mean depth: 4.5x), we detected instances of L1 insertions in cfDNA. Below, we present an overview of the analysis and one representative example from sample B4 as new Supplementary Figure 3d and e:

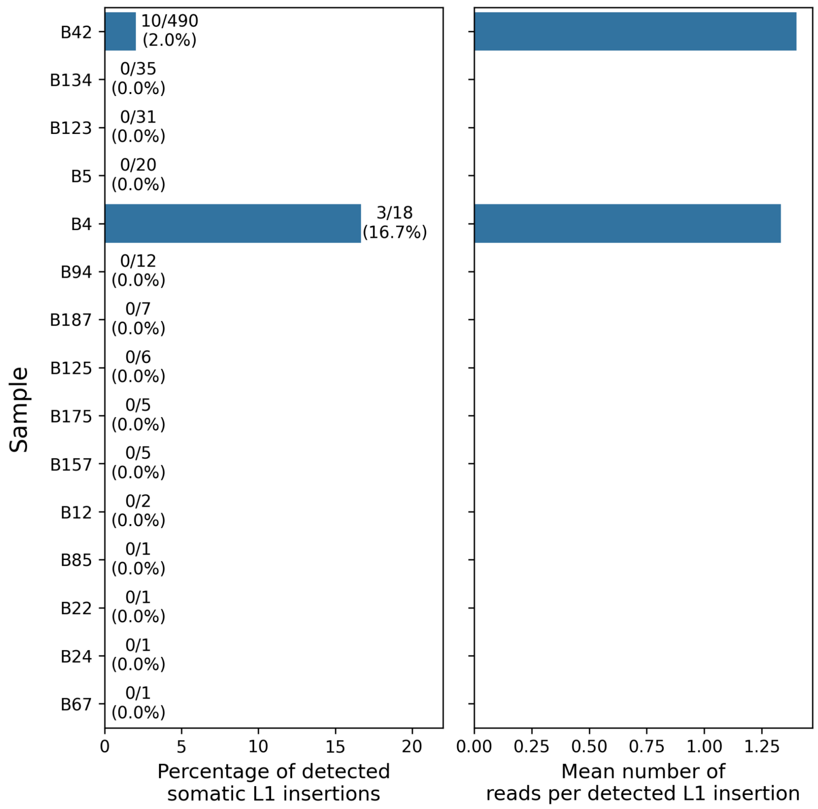

Supplementary Figure 3d: Overview of somatic L1 insertions detection in cfDNA. The left panel shows the percentage of somatic L1 insertions that were detected in cfDNA in each sample (number of detected somatic L1 insertions / all somatic L1 insertions). The right panel represents the mean number of reads supporting each detected somatic L1 insertion. Only samples that are L1-positive based on the long-read sequencing are shown.

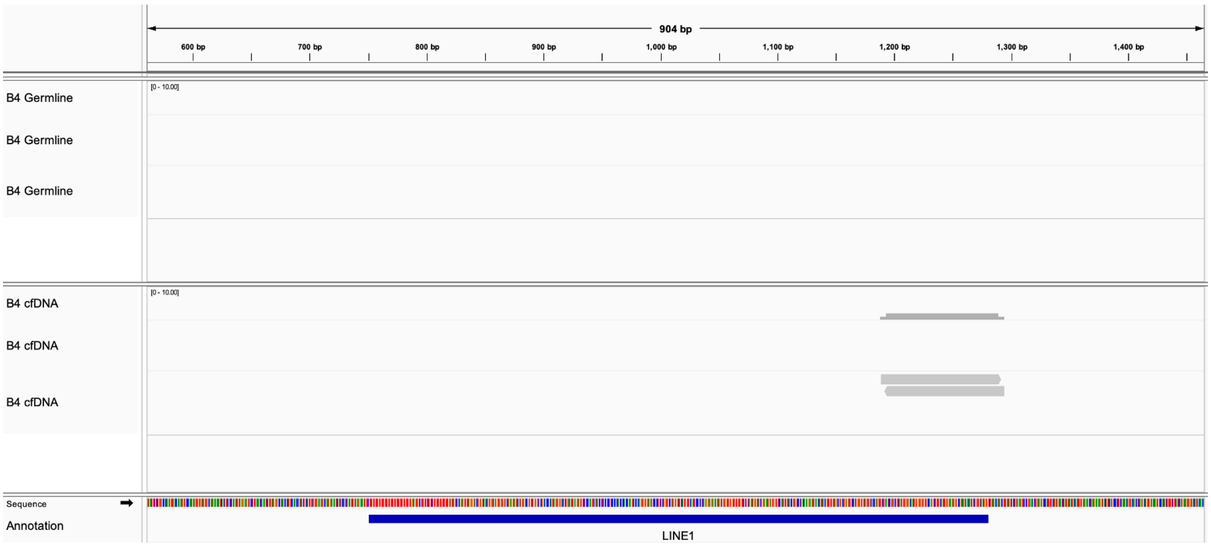

Supplementary Figure 3e: A representative example of a detected somatic L1 insertion in cfDNA for the sample B4 visualized in IGV. The upper track shows the B4 germline sample (DNA from white blood cells, whole-genome sequencing), while the bottom track shows the B4 cfDNA sample.

Reviewer #3-7: Manuscript structure
The manuscript is extremely dense and could be more reader-friendly.
- The two first sections of the introduction should be merged.
Response: As suggested, we have now merged the first two sections of the introduction.

- The Results section mixes discovery, validation, and speculation.
Response: We have revised the text to move speculative statements to the discussion.

- Consider a clearer structure. I would suggest:
1- Multi-omic landscape
2- L1 discovery and validation
3- L1–ecDNA relationship
4- Viral mimicry and immune contexture
5- Integrative model
Response: We have now revised the structure of the sections of the manuscript and added an integrative model (new Figure 5i).

Minor comments:
- Figures 2–4 should be improved; axes and legends are often too small.
Response: As suggested, we have now increased the size of the axes and legends.

- Provide accession numbers for sequencing data
Response: We have now added the accession ID of the omics data (GHGAS23497594176126) in the "Data Availability" section.

- Clarify in the abstract that causation between L1 and ecDNA is hypothesized.
Response: As suggested, we have now revised the abstract to clarify the fact that the causative link between L1 and ecDNA is putative.

- In the Introduction, include reference to bladder cancer-specific genomic instability studies (e.g., Robertson et al., Cell 2017; Kamoun et al., Eur Urol 2020).
Response: As suggested, we have now added references to both studies to the revised introduction.

- Methods section should mention quality control and filtering criteria for L1 and ecDNA calls.

Response: We have now added all details on quality control and filtering criteria for L1 and ecDNA calls (see revised Methods, "ecDNA identification", "de novo assembly of source and somatic L1", and Supplementary Table L).

- Typo note: "Long interspersed element 1" should be consistently abbreviated as LINE-1 (L1), not alternately L1 or LINE1.
Response: We now use L1 throughout the manuscript (with the exception of LINE-1ORF1P).

Reviewer #4 (Remarks to the Author):

This is a well-organized manuscript in which the authors resolve bladder cancer using multi-omics approaches. Short-read WGS, long-read WGS, bulk and spatial transcriptomics were integrated to profile the somatic variants and dissect the tumor microenvironment. However, here are some major concerns.

Major:
Reviewer #4–1: Study design: Are benign or normal tissues available in the tissue bank? The study has been focusing on the tumor tissue and the blood samples, while it's unclear if the differences are caused by tumor or tissue. If benign, normal or paired bladder tissues can be measured in the same way (long-read WGS, bulk or spatial transcriptomics), the control group will be clearer.

Response: We appreciate the reviewer's thoughtful comment. Paired bladder tissue samples were not available for this cohort, unfortunately. However, benign bladder tissue was available for five patients (B182, B192, B212, B218, B197), as well as matched blood. Short-read whole-genome sequencing was performed for all benign tissues and corresponding blood controls to address this question. As we have shown for the short-read and long-read sequencing data of the B42 tumor (see reviewer comment #3-2), short-read mobile element insertion callers show reduced sensitivity towards somatic L1s but can reliably identify the subset of L1s that have short poly-A tails and small target site duplications and insert in unique genomic context. Similarly, short-read methods are routinely applied for ecDNA detection but appear to show reduced sensitivity compared to long-read approaches in repetitive contexts (Sun et al., Cell Rep, 2025).
For the benign tissue and blood samples, we applied MELT, a state-of-the-art short-read mobile element caller, to identify putative L1 insertions present in benign tissue but absent from matched blood. This analysis yielded only 1 - 4 candidate tissue-specific L1 insertions per sample. Manual inspection in IGV indicated that these candidates were mostly not true benign-specific events, but

instead reflected either false negative calls in the matched blood due to coverage variability (**Fig. R4-1.1** and **R4-1.2**) or likely false-positive calls in benign tissue. As an additional control, reversing the comparison (blood versus benign tissue) similarly identified 1-2 private L1 insertions in blood that were not supported by the benign tissue data. Overall, the very small number of putative tissue-specific variants (1-4) identified among the total L1 insertions called by MELT per sample (105-108) suggests that these events mostly reflect false negatives in the matched blood or false positives in the benign tissue samples.
In parallel, we performed standard somatic SNV and InDel calling using Sage (Hartwig Medical Foundation) and Mutect2. Both methods identified relatively low numbers of tissue-specific variants (Sage: 23–517; Mutect2: 170–1332), in particular after taking the consensus of the two methods to increase specificity which led to only 2 tissue-specific variants in B182, 287 in B192, 3 in B197, 40 in B212 and 4 in B218.
To assess whether benign tissues showed evidence of genomic instability or extrachromosomal DNA (ecDNA), we ran AmpliconArchitect, a tool that identifies one or more connected genomic regions which have simultaneous copy number amplification and structural variants connecting the amplicons. For the benign samples, we did not obtain any high-quality ecDNA predictions, which suggests that the ecDNA formation is characteristic for bladder tumor tissue and drives oncogene expression. The tumor-specificity of ecDNA observed by us is in line with prior works from other cancer types conducted by the ecDNA community (Luebeck at al., 2023, PMID: 37046089).

In summary, we observe very few tissue-specific L1 insertions in the benign samples and only a small number of benign tissue-specific somatic SNVs and InDels, likely reflecting minor clonal expansions in somatic tissue. We find no evidence of ecDNA and therefore conclude that the observed patterns most likely originate from the tumor and cannot be explained by bladder tissue alone.

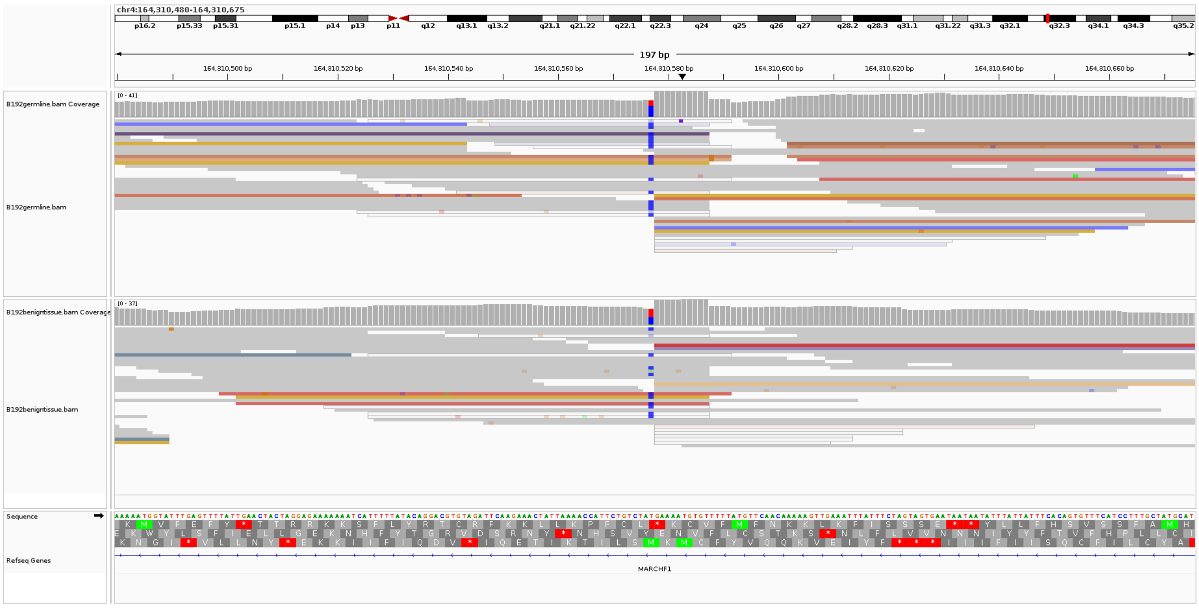

Figure R4-1.1: Manual inspection in IGV indicates that candidate L1 insertions in benign samples were not true benign-specific events, but instead reflected false negative calls in the matched blood blood. A representative locus predicted to harbor an L1 insertion in the benign tissue sample (lower panel) compared to the blood control (upper panel) is shown. Manual IGV analysis shows the characteristic split reads and target site duplication signatures in both samples, suggesting a missed insertion call by MELT in the blood sample (false negative).

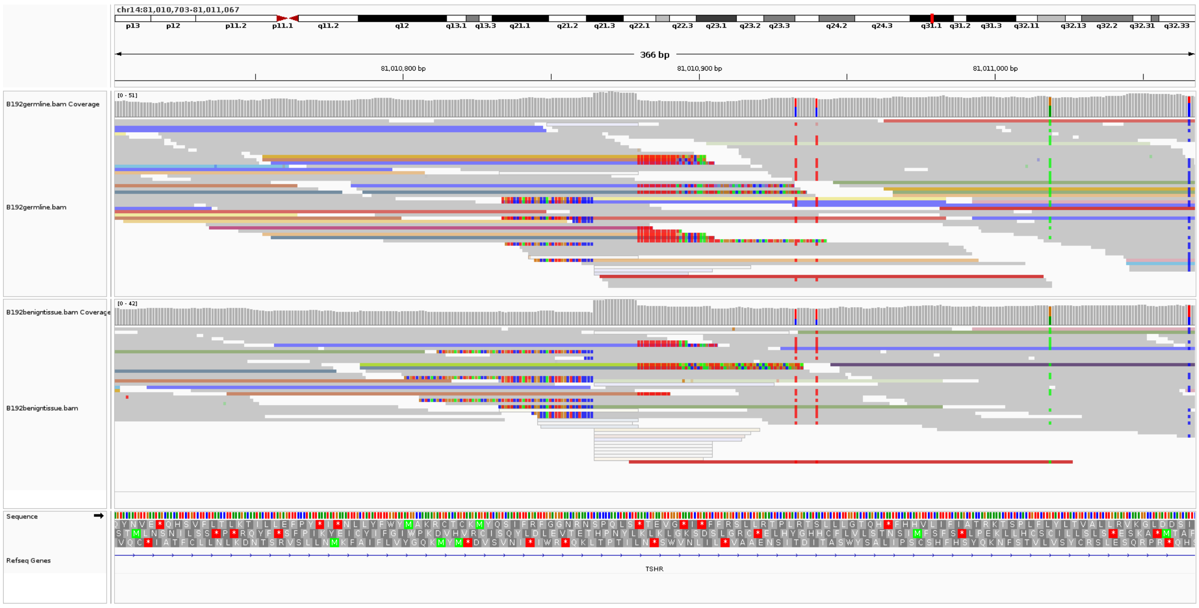

Figure R4-1.2: Manual inspection in IGV indicates that candidate L1 insertions in benign samples were not true benign-specific events, but instead reflected false negative calls in the matched blood blood. A representative locus predicted to harbor an L1 insertion in the benign tissue sample (lower panel) compared to the blood control (upper panel) is shown. Manual IGV analysis shows the characteristic split reads and target site duplication signatures in both samples, suggesting a missed insertion call by MELT in the blood sample (false negative).

Reviewer #4-2: Please provide more QC metrics for different omics measurements. For example, only the mean coverage across all the samples was provided, while not for individual samples. Will the sequencing depth (or accuracy, quality, GC content, etc) influence the CNV and mutation detection? For example, the library with low coverage by long-read WGS will yield a smaller number of variants.

Response: As requested, we have now added standard QC metrics for the ONT data (N50 read length, coverage, median read length and error rate) for each sample in Supplementary Table K. QC metrics are in the range of other recent studies in the field (e.g., PMID: 39406235). We also included the read length, coverage and insert size for the matched paired-end short-read germline data (Supplementary Table J).

While sequencing depth is known to affect the sensitivity of somatic variant detection, a comparison of our consensus-based somatic SNV counts from DeepSomatic and ClairS at different coverage thresholds starts to plateau at whole-genome coverages >= 20x for B42 (**Fig. R4-2.1**) and 22 out of 23 long-read sequenced tumors are above that threshold.

In addition, our use of an ensemble variant caller approach for somatic SNVs (ClairS and DeepSomatic) as well as somatic SV calling (Severus and Delly) was mainly motivated by prioritizing specificity over sensitivity to robustly identify mutational signatures and contributors to genetic instability such as somatic retrotransposition. We acknowledge that such a conservative strategy cannot comprehensively cover all somatic subclonal variants with a long-read whole-genome coverage in the range of 19x - 43x (median 33x) for all samples. We have now added this aspect to the limitations of our study (page 13-14).

Regarding the influence of GC content on mutation detection, we note that ONT sequencing is generally not subject to pronounced GC-dependent coverage bias (e.g., Browne et al., GigaScience, 2020), unlike short-read sequencing. As a result, ONT provides more uniform genome-wide coverage, largely independent of GC content.

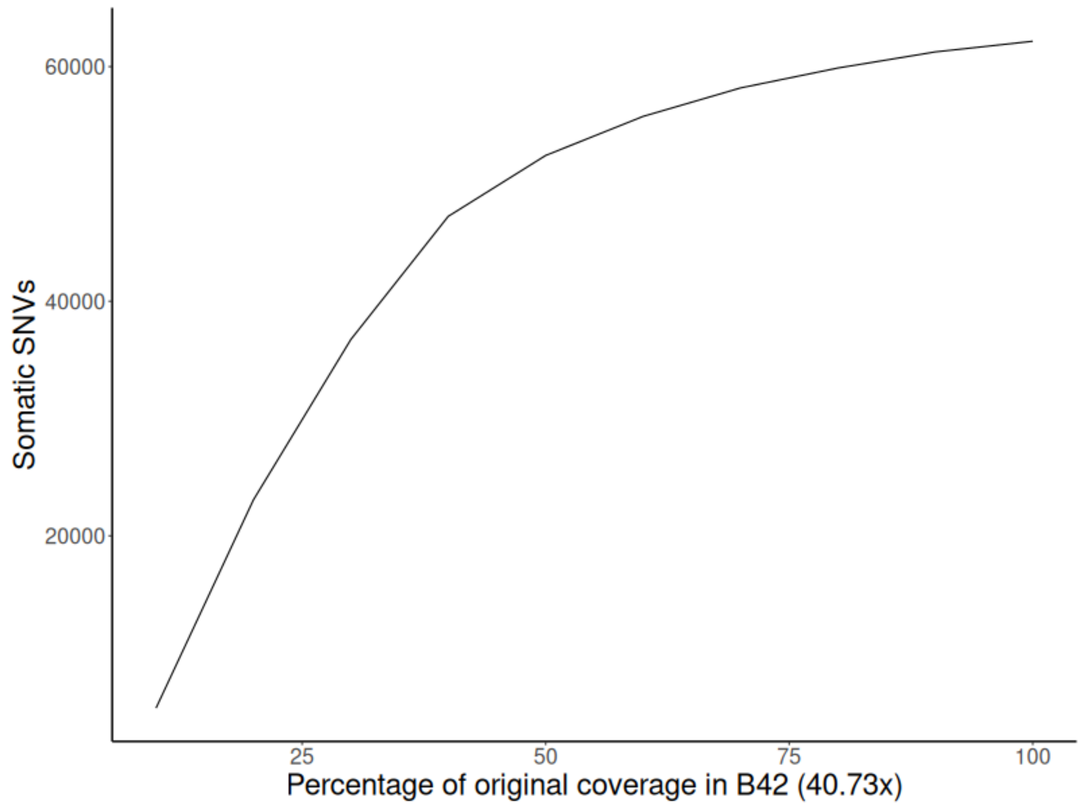

Figure R4-2.1: Count of somatic SNVs called by ClairS and DeepSomatic as a function of the downsampled input coverage for B42 with an original coverage of 40.73x

Reviewer #4-3: The ONT technology has been reported to reach lower sequencing accuracy when compared with short-read sequencing. How did the authors specifically address this concern when calling mutations from long-read data. How to control the false positives? In the method section, it's unclear how the top or high-confident variants were screened out.

Response: We thank the reviewer for raising this important point regarding the accuracy of ONT sequencing and the control of false-positive variant calls.
The impact of sequencing accuracy depends strongly on the variant class being interrogated. For structural variants (SVs), long-read sequencing technologies such as ONT have been shown to outperform short-read sequencing despite a higher sequencing error rate due to their ability to span repetitive regions and complex breakpoints (e.g., Sedlazeck et al., Nat Methods, 2018; Mahmoud et al., Nat Rev Genet, 2019). In addition, we applied a consensus approach of Severus and Delly and such ensemble approaches have been shown in the past to improve specificity by reducing the number of false positives SV calls (e.g., Zarate et al., GigaScience, 2020).
We do acknowledge that small variants, particularly InDels and to a lesser extent SNVs, are more sensitive to lower sequencing accuracy in long-read data. To explicitly address this concern and reduce false positives, we have improved our

small variant calling strategy. Specifically, we now define high-confidence somatic variants as those supported by a consensus between two independent long-read somatic variant callers, DeepSomatic and ClairS. As tumor-only somatic variant calling approaches can overestimate the tumor mutational burden (TMB, e.g., Parikh et al., JAMA Netw Open, 2020), we in addition genotype all called somatic variants in the matched controls and subsequently remove all variants that are present in the matched germline. We have updated the Methods section to clearly describe this consensus-based filtering strategy and to explicitly define the criteria used to retain high-confidence variants.

We also compared our somatic SNV calls to standard short-read approaches for B42 and B125 where we sequenced the tumors using both short-read and long-read approaches. This analysis confirmed that private somatic SNV calls, identified only by DeepSomatic or ClairS, are rarely supported by short-read data, and that genotyping somatic variants in short-read controls eliminates many germline artifacts that are poorly captured by tumor-only approaches with a standard panel-of-normals (**Fig. R4-3.1 and Fig. R4-3.2**).

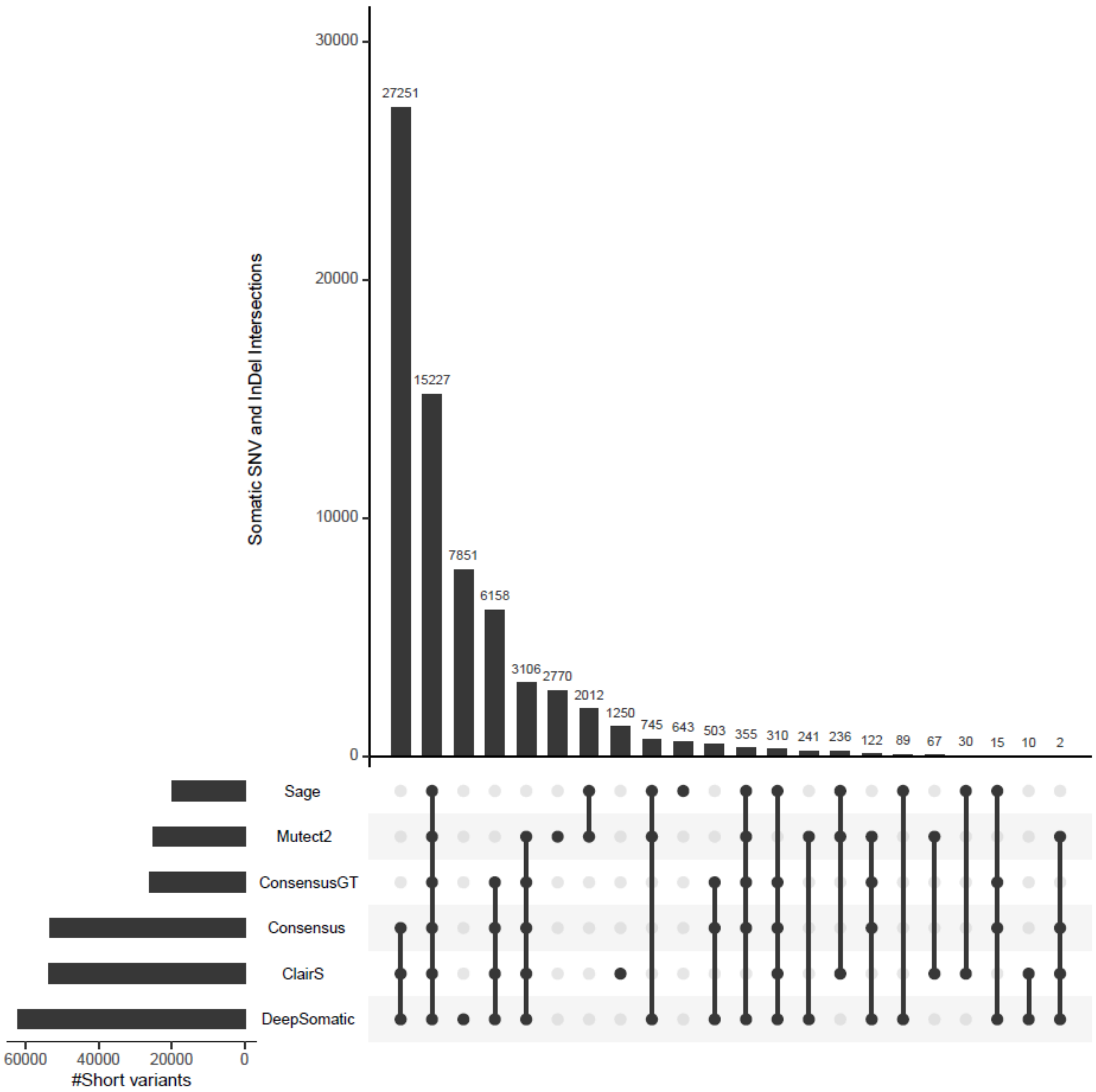

Figure R4-3.1: Bi-allelic somatic short-variant call sets (SNVs and InDels) of ClairS, DeepSomatic, the consensus of ClairS and DeepSomatic (Consensus) and the consensus calls genotyped and filtered against the matched controls (ConsensusGT) in comparison to standard short-read analysis pipelines (Sage - Hartwig Medical Foundation, and Mutect2 - nf-core/sarek tumor-normal workflow) for B42 in Genome in a Bottle (GiaB) high-confidence regions for GRCh38.

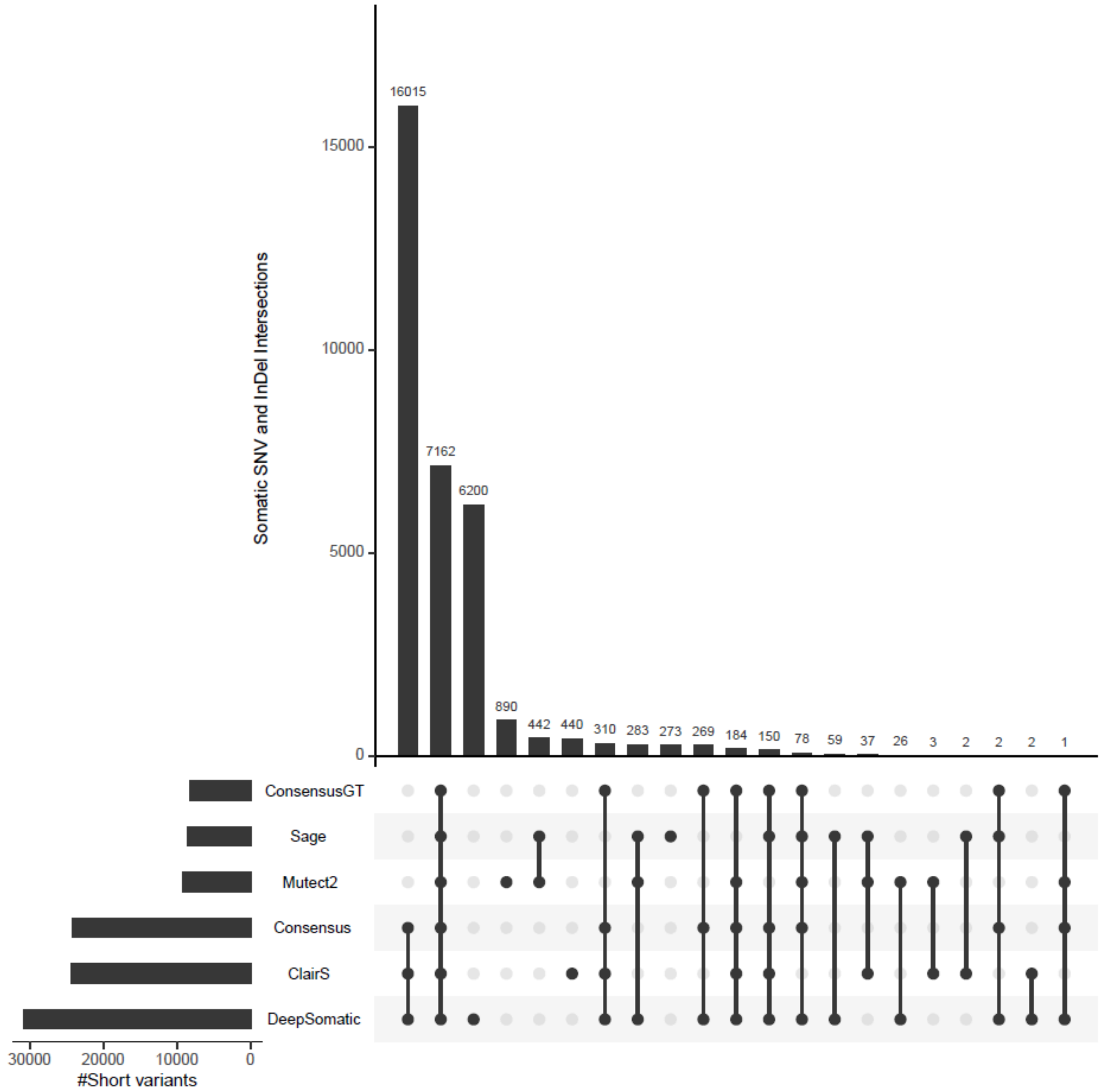

Figure R4-3.2: Bi-allelic somatic short-variant call sets (SNVs and InDels) of ClairS, DeepSomatic, the consensus of ClairS and DeepSomatic (Consensus) and the consensus calls genotyped and filtered against the matched controls (ConsensusGT) in comparison to standard short-read analysis pipelines (Sage - Hartwig Medical Foundation, and Mutect2 - nf-core/sarek tumor-normal workflow) for B125 in Genome in a Bottle (GiaB) high-confidence regions for GRCh38.

Reviewer #4–4: Have the SVs, SNVs and gene fusions been fully or partially validated by experimental molecular approaches, such as PCR, Sanger sequencing, etc? If not, I would highly suggest that the authors choose the top variants in each category to validate. For example, the 2 fusion in Supp Figure 1, the 12 high-impact variants the ones listed in Figure 1b.

Regarding the validation of **SNVs and InDels**, please kindly refer to our response to point #4-3 above, showing the results of additional data and analyses that we performed to address this question.

Regarding the validation of **SVs**, please kindly refer to the response to point #3-2 above: we validated the high number of somatic L1 insertions using comparisons between ONT and newly generated short-read sequencing data, with a validation rate of 93.5%.

For the two **gene fusions** shown in Supplementary Figure 1, we have expanded the figure to include IGV screenshots of reads spanning the fusion breakpoints (see revised Supplementary Figure 1). These breakpoints are supported by high read coverage at both the DNA and RNA levels, providing strong orthogonal evidence for the presence of true fusion events.

In addition, for all gene fusions identified across the entire cohort, we now provide a comprehensive Supplementary Table D. For each fusion, this table reports the precise breakpoint coordinates at both the DNA and RNA levels, the corresponding read support, and the fusion callers that independently detected the event at the given locus.

Reviewer #4–5: Please provide the accession ID of the omics data in the 'Data Availability' section. The authors can keep the data in private status until the manuscript acceptance/ publication.

We have now added the accession ID of the omics data in the "Data Availability" section. The data generated in this study are available via controlled access in the German Human Genome-Phenome Archive (GHGA, data.ghga.de under the GHGA Accession GHGAS23497594176126).

Reviewer #4–6: Microbial infection analysis: other than ONT data, bulk and spatial transcriptomics data can also detect and estimate microbial (bacteria and virus) expression. Given the multi-omics scope of this project, I would highlighly recommend the authors perform comprehensive microbial infection analysis using these omics datasets. They can also validate the finding from the ONT data.

We searched for bacteria from bulk RNA sequencing and confirmed among others the presence of Anaerococcus reads in the transcriptome.
Using Kraken2, 6,890 RNA-Seq reads of B42 (1.56% of all unmapped reads) were classified as Anaerococcus, underscoring the presence of this genus in both DNA and RNA-Seq data for B42. The most abundant genus in the RNA-Seq data was Bradyrhizobium (range 0.1% - 47.9%), which is another microbial species found to

be enriched by Sheng et al. (2025) in recurrent bladder cancer. These new results are now presented in the revised Supplementary Figure 11 c and d.

We also performed a targeted search for viruses previously implicated in bladder cancer (Baker, *et al.,* 2022, PMID: 35194151; Starrett *et al.,* 2023 PMID: 36961501), including human papillomavirus (HPV), Merkel cell polyomavirus (MCPyV), BK polyomavirus (BKPyV), and John Cunningham polyomavirus (JCPyV). To detect potential viral integration events, we applied BreakTracer to tumor genomes of the patient cohort using multiple viral sequence identity thresholds (0.89, 0.70, and 0.65). All viral sequences were retrieved from NCBI database and have following accession numbers: LR215986.1 (BKPyV), KJ194499.1 (HPV), NC_001699.1 (JCPyV), HM011556.1 (MCPyV). However, no evidence of viral integration was detected in any sample of our cohort across all thresholds.

In addition, we searched for the presence of viral sequences by classifying unmapped RNA-seq reads using Kraken2, as described above for the microbial analysis. Specifically, we searched for members of the Polyomaviridae family (BKPyV, JCPyV, MCPyV) and the Papillomaviridae family (HPV). This analysis likewise did not reveal any evidence of these viruses in this patient cohort.

As previously noted by Baker *et al.* (2022), the absence of detectable viral integration does not exclude the presence of episomal viral genomes such as BKPyV , which may evade detection by integration-focused approaches. Furthermore, BKPyV and JCPyV have been most strongly associated with bladder cancer in specific contexts, such as organ transplantation and immunosuppression.

Regarding the use of spatial transcriptomics data to detect and estimate microbial expression:
As Visium for FFPE is a probe-based platform, it is unfortunately not possible to do a microbial infection analysis from these spatial transcriptomics data.

Reviewer #4–7: Figure 4: Spatial clustering was performed, while the biological interpretation of the clusters are not clear. The authors only focus on cluster 0 and 1, but not the others. In addition, 10 spatial transcriptomics were performed. Can their clusters be matched together? Any difference among their clinical features, such as the low vs high grade, MIBC vs NMIBC, tumor number and size, or age?

We have now added details on the biological interpretation of the spatial clusters beyond clusters 0 and 1 (see revised text, page 9 and new Supplementary Figure 17c). In addition, we now provide a new Supplementary Table F with markers associated with each cluster:

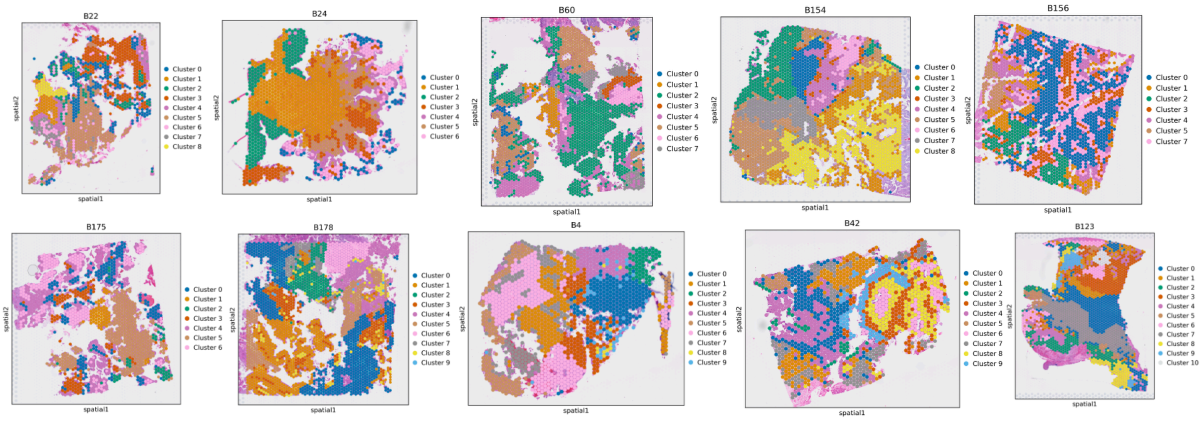

Supplementary Figure 7: SpatialDE2 segments across all the samples.

As suggested, we performed an integration of SpatialDE clusters from all samples:

Transcriptional clusters were first identified with SpatialDE2 within each sample. For each SpatialDE2 cluster we generated pseudobulk expression profiles, but excluding SpatialDE2 clusters with 3 or less spots. Next, pseudobulk profiles from all the samples were integrated using ComBat batch correction. Highly variable genes in the pseudobulk matrix were used for dimensionality reduction by PCA, followed by constructing a neighborhood graph on the first 10 PCs. To identify the number of meta-clusters, we performed Leiden clustering with a sweep on resolution to identify the most stable solution. UMAP was used for visualization of pseudobulk profiles.

To characterize the biological programs associated with each meta-cluster, pathway activities were inferred using the univariate linear model (ULM) implemented in decoupler. Pathway enrichment was performed using MSigDB Hallmark gene sets.

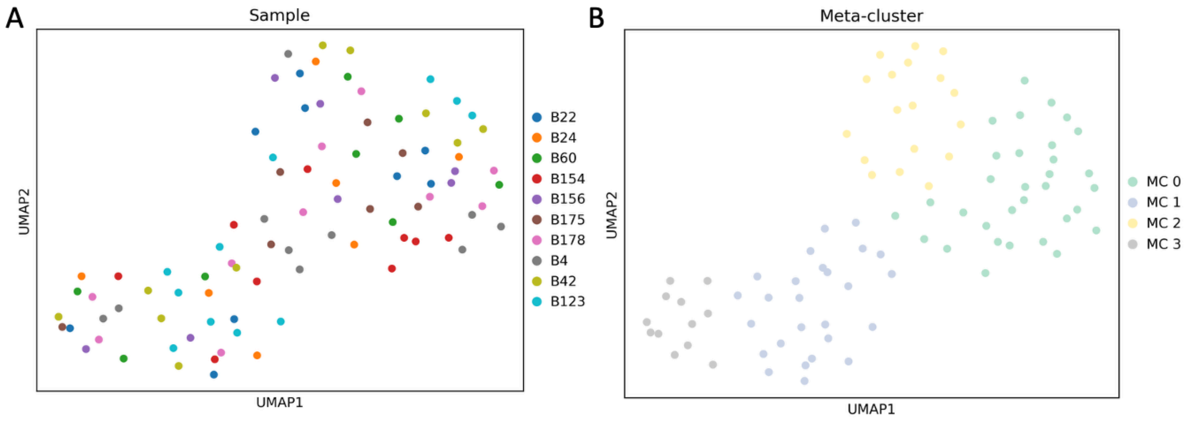

Figure R4-7.1: UMAP after integration of pseudobulk SpatialDE2 segments colored by A) sample and B) assigned meta-cluster.

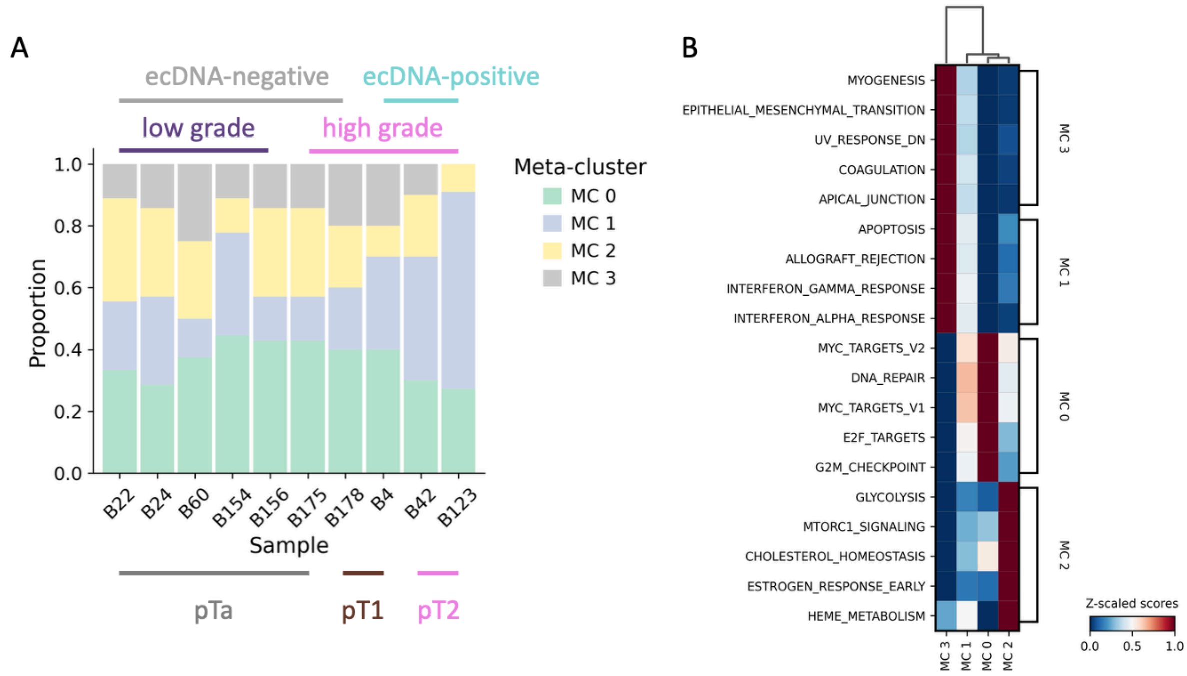

Figure R4-7.2: A) Proportions of meta-clusters per sample. B) Enrichment of Hallmark pathways per meta-cluster.

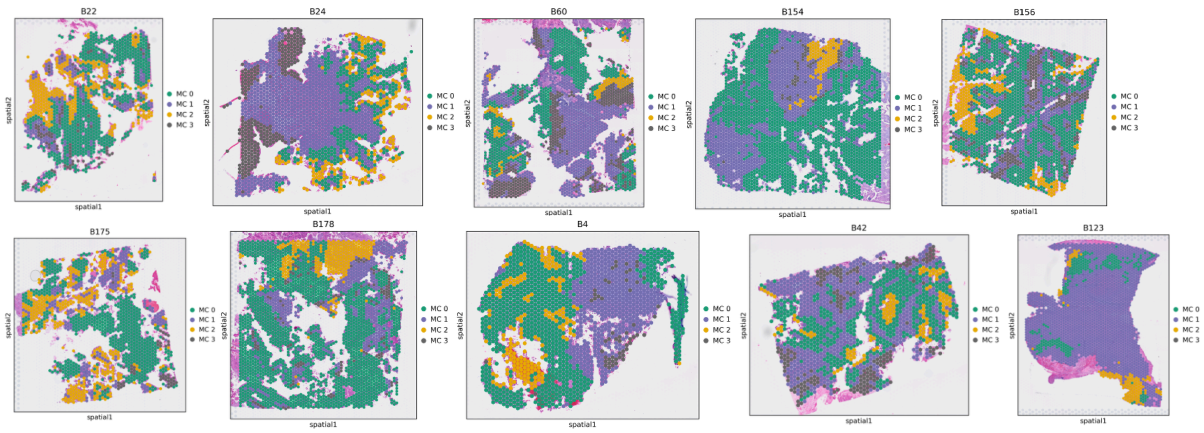

Figure R4-7.3: Meta-clusters across all 10 samples in space.

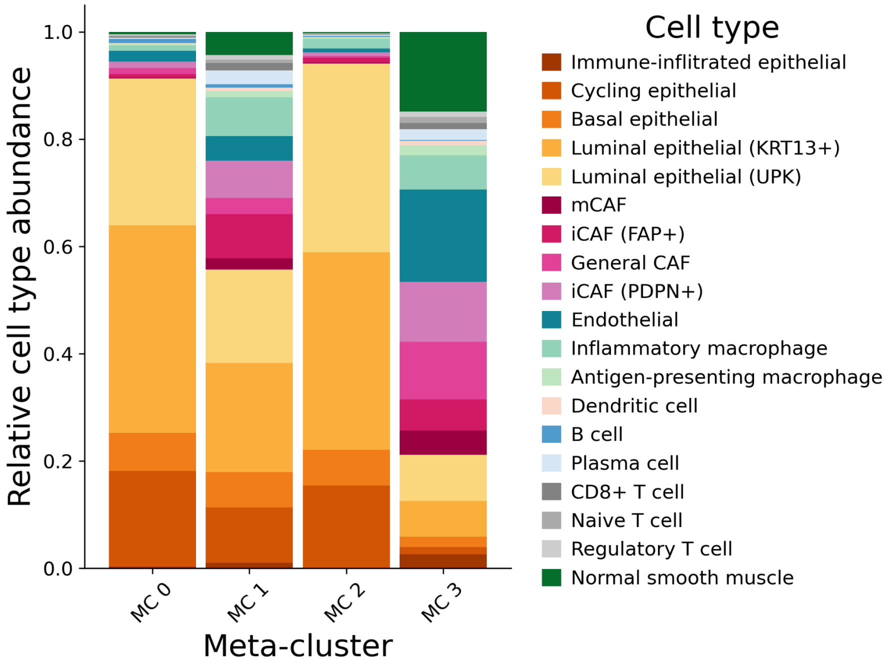

Figure R4-7.4: Cell type composition per meta-cluster across all 10 Visium samples.

This analysis showed that to some extent, clusters can be matched between patients, and the four major meta-clusters (that also show spatially coherent regions) are present across all patients and defined by enrichment of specific hallmarks and cell types. Meta-clusters 0 and 2 represent tumor-rich regions, with meta-cluster 0 characterized by a strong enrichment of cell-cycle and proliferation related pathways and meta-cluster 2 by an enrichment of metabolic pathways. In contrast, meta-cluster 3 is enriched for stromal and mesenchymal programs and shows high proportions of fibroblasts and endothelial cells. Meta-cluster 1 does not display any dominant pathway enrichment and exhibits a mixed cell type composition.

Regarding the correlation of spatial clusters with clinical features, Supplementary figure 6e displays the cell type abundance across samples annotated with clinical features such as tumor grade and segregated by ecDNA content.
In addition, we now show the meta-clusters annotated with clinical features (see R4-7.2) and test whether the spatial heterogeneity (number of segments per spot) correlates with clinical features:

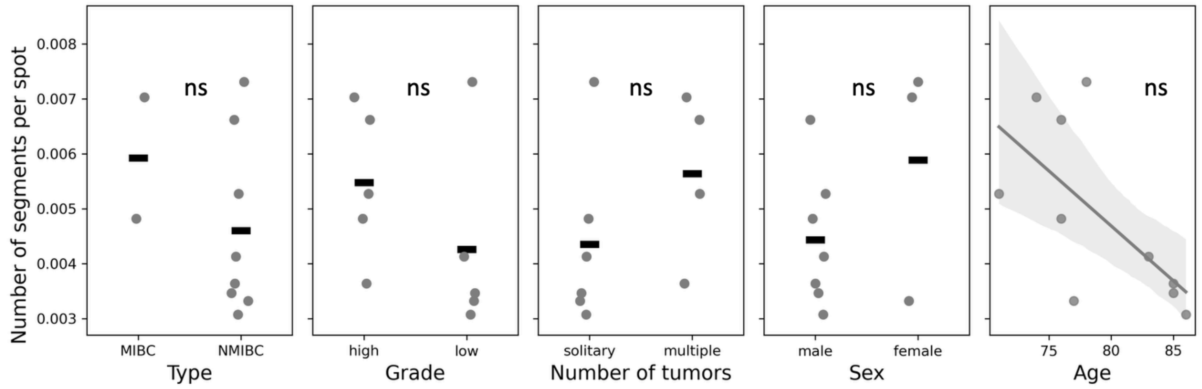

Figure R4-7.1: Associations between transcriptional heterogeneity (the number of SpatialDE2 segments per spot) and clinical features were tested using Mann-Whitney-U test for binary variables and Spearman's rank correlation for age. To account for multiple hypothesis testing across the five clinical features, p-values were adjusted using the Benjamini-Hochberg false discovery rate procedure.

Even though the differences are not significant with this cohort size and after correcting for multiple testing, higher grade and higher number of tumors per patient seem to be potentially associated with higher spatial heterogeneity.

Reviewer #4-8: Figure 4: The transcriptome and multi-omics level analyses are not sufficient. For example, given the spatial Visium and bulk RNA-seq data, the authors can perform cell annotation (Figure 4c has clusters but no cell type annotation), cell type deconvolution, cellular interaction, and trajectory inference. The authors can also integrate the variant calling with the expression level analysis to resolve bladder cancer in a systematic manner.

The cell type annotation and deconvolution analyses are shown in Supplementary Figures 6 e-f, we have now adjusted the main text to clarify this (see revised results section, page 5). We have also added a representative example to revised Figure 2f for clarity.

As suggested, we have performed a cellular interaction analysis. We applied COMMOT on the Visium data. The cell-cell communication analysis identified among others SPP1 signaling as a major pathway active in this bladder cancer cohort, which is involved in cell adhesion and migration as well as immune and inflammatory regulation. Spp1 is involved in bladder cancer progression and aggressiveness, by promoting invasion and metastasis but also tumor microenvironment remodeling. In addition, LINE-1 activation can create conditions that favor SPP1 expression, especially in tumor cells or macrophages.

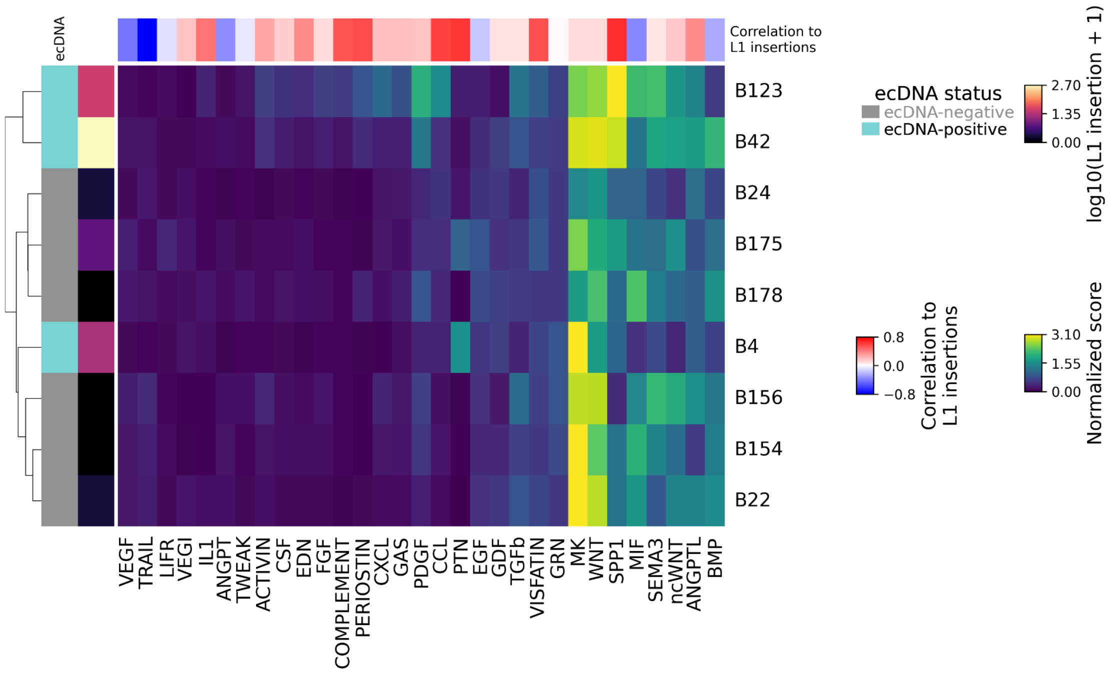

Figure R4-8.1: Normalized cell-cell communication pathway scores calculated using COMMOT. Each row represents one sample and each column represents one pathway. Annotations on the left show the ecDNA status of the sample and the number of L1 insertions. Annotations on the top of the heatmap show Pearson correlation coefficient of each pathway to the number of L1 insertions per sample.

As suggested, we have now performed a trajectory inference analysis on the Visium data using stLearn. This new analysis identifies differences in the differentiation status of cells present in distinct clusters with different pseudotimes and identifies a number of genes (including *APOBEC3B*) as significantly associated with pseudotime (see **Figure R4-8.2** below). However, as these pseudotime and trajectory analyses are debatable without extensive validations, we would prefer keeping these figures as rebuttal figures, at the editor’s discretion.

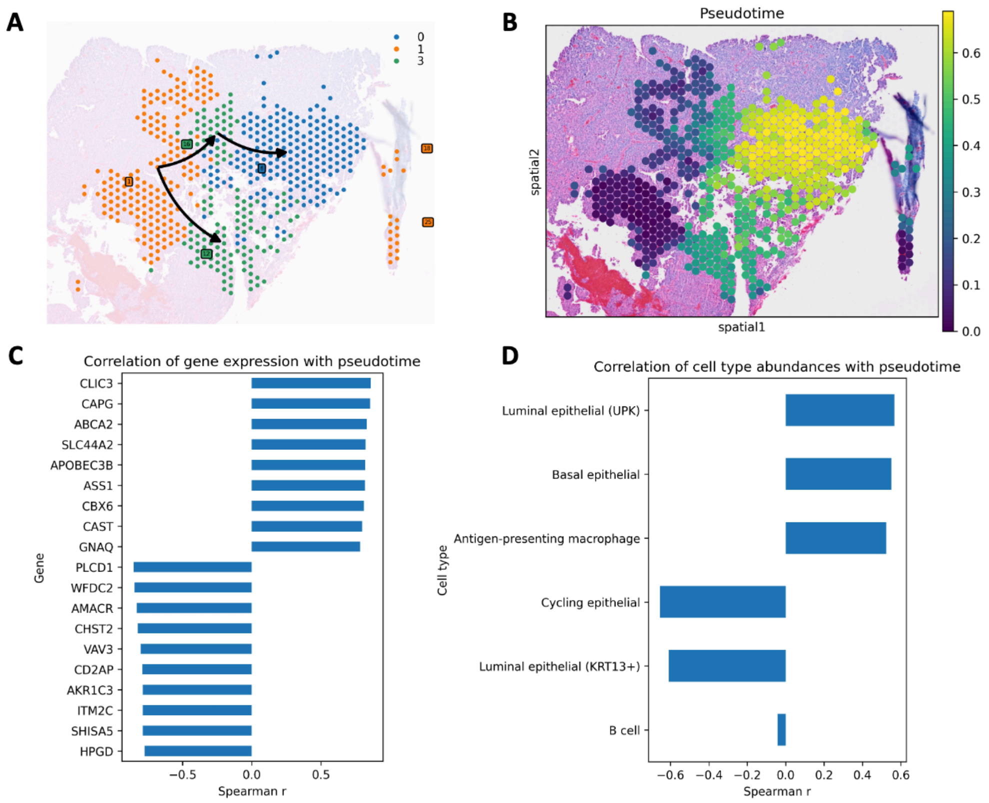

Figure R4-8.2: Trajectory analysis of the sample B4 using stLearn.
a) Trajectory analysis of the region of interest, including two ecDNA-enriched regions and an intermediate cluster. Cluster 1 was selected as the root for the analysis. b) Pseudotime in space. c) Top ten genes showing strongest positive and negative Spearman correlation with pseudotime. d) Top three cell types with the strongest positive and negative Spearman correlation with pseudotime.

As suggested, we have now integrated the variant calling with the expression level analysis. We performed an integrated analysis of haplotype-resolved differential methylation of gene promoters, single-nucleotide variants (SNVs), and RNA expression as described in the added methods section. This analysis identified two samples (B94 and B187) in which one haplotype harbors a variant and exhibits significant hypemethylation of a corresponding promoter ($q < 0.05$) and altered gene expression. These results are presented in Supplementary Figure 15.

In addition, as a further integration of the variant calling with the expression level analysis, we previously reported evidence of enhancer hijacking involving tumor-promoting genes, such as *UCHL1*, based on *pyjacker* analysis (Supplementary Figure 4a–d).

For convenience, we copied the methods section from the main paper that describes the analysis in detail, below.

Integrative analysis of methylation, SNVs and RNA-seq expression

Promoter regions for all genes in the hg38 reference genome were extracted using the GRanges R package and defined as 2,000 bp upstream and 400 bp downstream of the transcription start site (TSS). Only unique promoters mapping to canonical chromosomal regions were retained for downstream analysis. Differential methylation between haplotypes was assessed for each tumor sample using *modkit dmr pair*. Statistical significance for each promoter region was evaluated using a binomial test, followed by Benjamini–Hochberg correction for multiple testing. Promoters were considered significantly differentially methylated if they met the following criteria: $q < 0.05$, modkit score > 50, and absolute haplotype methylation effect size > 0.2.

Significantly differentially methylated promoters were subsequently overlapped with single-nucleotide variant (SNV)calls and matched with corresponding normalized RNA-seq gene expression values. In addition, genes harboring haplotype-specific differentially methylated promoters were annotated with COSMIC cancer gene status (v103)obtained from the COSMIC database.

Reviewer #4–9: This manuscript focuses on the L1 elements. It couldn't be better to show the overall variant distribution, such as 5' UTR, intron, exon, 3' UTR, or other transposable elements, such as SINE or LTR.

We have now added analyses showing the overall variant distribution (e.g. L1, Alu, SINE, SVA…) as well as the L1 somatic insertion distribution across genomic features (e.g. intron, exon, intergenic…). Please note that we did not detect any somatic LTRs in our samples, and no L1s inserted in 5' UTR or 3' UTR regions. These data are presented as Supplementary Figure 8f-i.

Reviewer #4–10: Please discuss the limitations and future works of this project in the Discussion section.
As suggested, we have now added a section on the limitations of the study and potential future directions to the revised discussion.

Minor:
1. Figure 2B: Please highlight the MIBC and NMIBC in the top bar. Do they show differential patterns in SCNA and mutation patterns?
As suggested, we have now highlighted the MIBC and NMIBC tumors in the legend of Figure 1b (we assume the reviewer means Figure 1b and not 2b). The improved visualization reveals distinct genomic patterns, with MIBC samples exhibiting increased SCNA burden and more complex mutation profiles compared to NMIBC tumors.

2. Figure 4c: Why is cluster 8 not available?

All clusters with less than 3 spots per cluster were excluded. We have now added this information to the revised Methods section.

3. Figure 4h and 4k: What do ‘false’ and ‘true’ represent? All the bars are green (true).

As only significantly dysregulated pathways are shown in mentioned figures, as correctly pointed out by the reviewer, we have now removed the ‘false’ category from the legend in revised figure 4.

4. Supplementary Figure 2: I would suggest the authors align the tumor long-read WGS with the cfDNA short-read WGS for the same patient, as it was shown in Fig 2a-b. Otherwise, there is no control for the germline variant calling.

Even though a few cfDNA samples show copy-number changes (e.g. Figure 2, Supplementary Figure 3), when considering the whole cohort, only a minority of cfDNA samples showed such copy-number changes, with evidence for enrichment in high-grade tumors (4/25 high-grade tumors, 0/23 low grade, p=0.06, Fisher’s exact test, one-sided).

Hence, we present the long-read WGS with the matched cfDNA short-read WGS for those samples for which copy-number changes were detected (B4, B67, B169, B123). Regarding controls for germline variant calling: As the coverage is higher in the short-read sequencing data of the white blood cells from which germline DNA was extracted (as compared to the cfDNA data), we used the short-read sequencing data of the white blood cells as germline controls. The coverage plots of these samples did not show any large copy-number variants (**Figure R4-10.1)**.

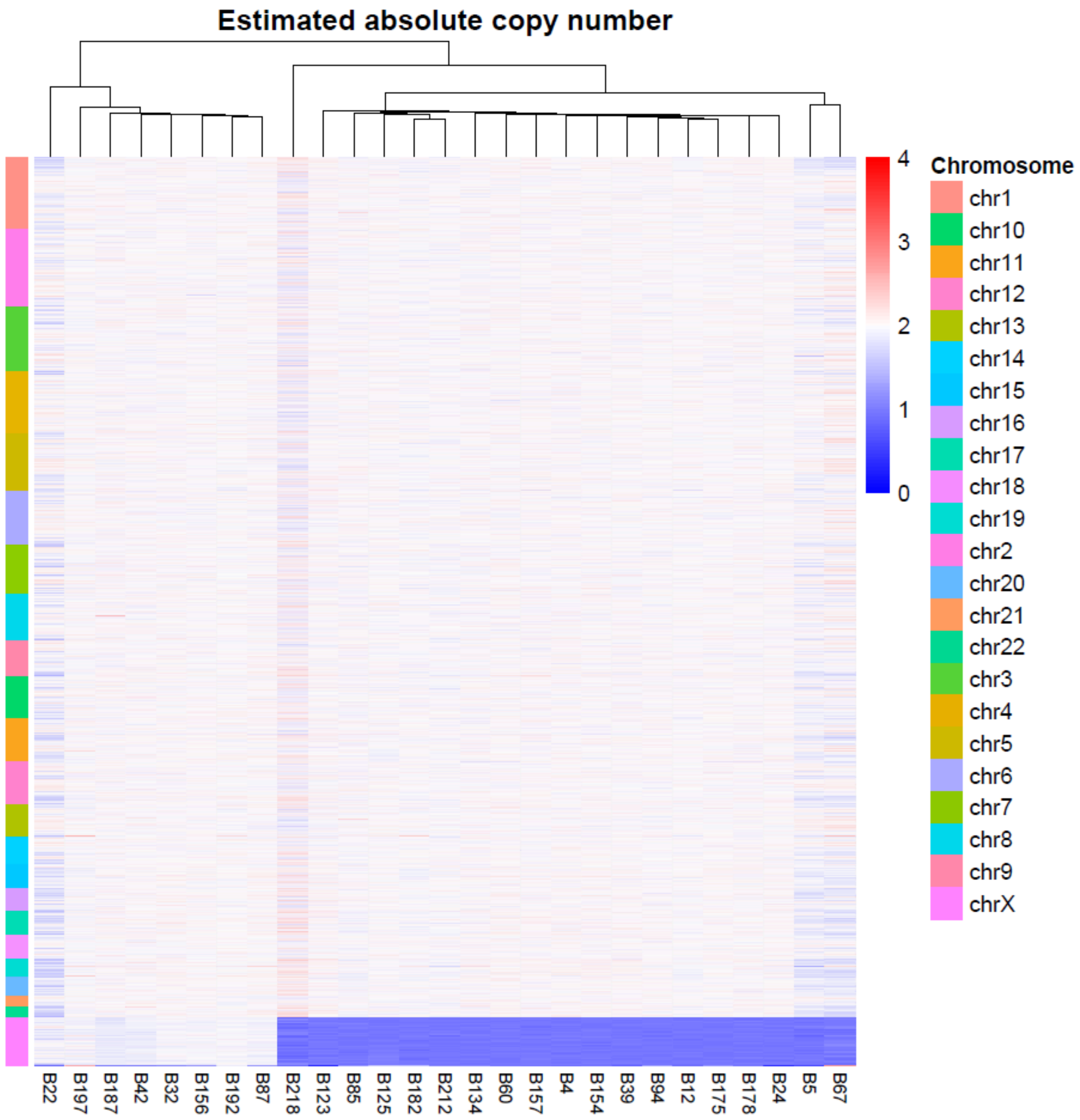

Figure R4-10.1: Heatmap of estimated absolute copy-number of the germline samples (from white blood cells) across all autosomes and chrX, where the male samples show an expected copy-number of one.

In addition, coloring the variant positions with different backgrounds will help highlight the variants.

As suggested, we have now coloured the ONT plots like the cfDNA plots to help highlight the variants (see revised Figure 2a).

In addition to the points raised by the reviewers, we also updated the neoepitope analysis with the following improvements:

Neoepitopes were predicted in the new analysis from (I) all 18 patients in the L1-low and L1-high groups (instead of only the 12 patients with RNAseq data in the previous analysis), (II) by selecting only state-of-the-art prediction tools from pVACtools (instead of the full panel, which includes tools that are no longer maintained), and (III) by allowing also multiple neoepitope candidates from the same mutation.
As in the first version of the manuscript, the number of predicted neoepitopes is still significantly higher in L1-high compared to L1-low patients (revised Figure 5f).

Point by point response

Reviewer #1 (Remarks to the Author)

Dear Editor,
we reviewed the revised manuscript. The Authors addressed satisfactorily all the criticisms raised by our side.
The manuscript is markedly improved and we do not have further comments.(Remarks on code availability)

Reviewer #2 (Remarks to the Author)

I co-reviewed this manuscript with one of the reviewers who provided the listed reports. This is part of the Nature Communications initiative to facilitate training in peer review and to provide appropriate recognition for Early Career Researchers who co-review manuscripts.(Remarks on code availability)

I have send my comments to Roberto Chiarle, and he will submit all in one.

Reviewer #3 (Remarks to the Author)

The authors provided extensive responses to my concerns and comments.(Remarks on code availability)

Reviewer #4 (Remarks to the Author)

I would appreciate the great manuscript revision work! The authors have addressed all my concerns.(Remarks on code availability)

Reviewer #5 (Remarks to the Author)

In the manuscript entitled "Integrative spatial and multi-omic profiling in bladder cancer links L1 retrotransposition to extrachromosomal DNA, genomic instability, and viral mimicry response", Pribus et al profiled samples from 48 bladder cancer patients combining long-read sequencing, spatial transcriptomics, and other genomic approaches. They identified frequent L1 insertions as an early event of bladder cancer, which potentially drives subsequent genome instability, such as APOBEC3B-mediated mutagenesis and ecDNA formation, further promoting tumorigenesis. L1 activation has also been linked to downstream viral-mimicry effects. Overall, this is a comprehensive study using multi-omics to study genome instability in bladder cancer

progression. Despite being somewhat descriptive, the study provides rich genomic resources and infers how genomic instability events are linked to bladder cancer progression. Following the Editor's instructions, my assessment primarily focused on ecDNA.
Response: Thank you for this positive assessment of our work. Please find below our point-by-point response to your comments.

Comment 1. In supplementary figure 4e, the authors mentioned that most of the tumor-promoting genes activated through enhancer hijacking were independent of ecDNA formation. This is true and unsurprising because ecDNAs comprise only a small fraction of the cancer genome. However, according to this figure panel, 13 of the 21 ecDNA genes were involved in enhancer hijacking, if I read it correctly. Therefore, the message that ecDNA is a hotspot for enhancer hijacking should not be overlooked (e.g. PMID: 33199677). If readers do not carefully digest the original sentence, they may misunderstand the message. This is a minor point but needs to be revised.
Response: Thank you for this suggestion. We agree that this result needs to be highlighted more clearly and have included the following sentence and the reference in the revised manuscript: "Enhancer hijacking was frequent among ecDNA-associated overexpressed genes (Fisher test p-value $< 10^{-16}$), consistent with prior work showing that ecDNA co-occur with enhancer hijacking to express oncogenes (PMID: 33199677)."

Comment 2. In supplementary figure 14f, the authors claimed that higher L1 insertion is positively associated with the presence of ecDNAs. However, the P value was 0.0538 even under the one-sided test. I understand the challenge of sample size limitation that reduces statistical power. However, we should still be stringent. The sentence should be rephrased to show the trend by ecDNA stratification while acknowledging the insignificance due to limited power.
The same problem appears in Supplementary figures 14g-h. In 14g, the coefficient was indeed higher in the ecDNA+ cohort than in the ecDNA- cohort. However, whether this difference is significant remains unclear. The 14h panel addressed this question. However, it was insignificant even by the one-sided test. Again, I understand the challenge of a limited sample size. However, we should not send the wrong message to the field.
For 14h, another possible explanation for the enrichment of active L1 loci expression in ecDNA-positive patients is that some L1 elements may reside on ecDNAs and become transcriptionally activated due to the permissive chromatin environment associated with ecDNA. In this scenario, L1 activation would be a consequence rather than a driver of ecDNA formation. Notably, recent reports (PMID: 39282372) have suggested that retroelements located on ecDNA can be transcriptionally active. This possibility should be considered and at least discussed.
Therefore, all L1-ecDNA-related texts should be carefully revised, including in the abstract, which emphasizes the intimate relationship between L1 and ecDNA.
Response: Thank you for your feedback.
We agree that we must draw conservative conclusions. We have revised the text in the results and conclusion to clearly state that the trends we describe did not achieve statistical

significance and thus require further investigation with greater sample numbers to improve statistical power.

With respect to your suggestion that the enrichment of active L1 loci expression in ecDNA-positive patients could be due to L1 element presence on ecDNAs, we did consider this possibility and tried to identify whether the L1 insertions we identified were present on ecDNA rather than linear chromosomes. To do so, we looked at any insertions that overlapped with ecDNA regions: we found one such example in sample B42 and one in sample B123. We then used our haplotype-resolved long-read sequencing data and counted the total reads at each insertion position that supported the insertion versus no insertion. We reasoned that the highly amplified haplotype reads at each position would represent the ecDNA reads; thus, if an insertion was present on an ecDNA, there should be a high ratio of reads supporting the insertion versus not. Otherwise, if the ratio of insertion:no insertion reads was small, this would indicate that there was no insertion on the highly amplified ecDNA. In sample B42, the insertion support ratio was 5:23 (i.e., 5 reads supporting the insertion and 23 not supporting the insertion). In B123, the insertion support ratio was 7:81 (i.e., 7 reads supporting the insertion and 81 reads not supporting the insertion). Thus, we concluded there were no detectable L1 insertions on ecDNA in our samples, or that any such events occurred late in ecDNA evolution as subclonal somatic insertions. We have added these results to the revised text and methods.

With respect to the abstract, we believe the current wording maintains an appropriate balance and does not excessively foreshadow focus on the L1-ecDNA relationship in our study. With that said, we would gladly consider any editorial suggestions for adjustment.

Comment 3. I find it difficult to understand the "sample-specific ecDNA signature" method. To the best of my understanding, was the rationale as below?

Spatial transcriptomic signals were sparse, limiting the sensitivity of ecDNA cluster detection; therefore, the authors extracted gene expression signatures from high-confidence ecDNA clusters, using ecDNA gene-high cells as a surrogate to infer cells that may contain ecDNAs. This method was applied on a case-by-case basis. For example, the B4 signature was only used in the B4 sample, but not in other samples.

Alternatively, were the aggregated gene signatures from 3 samples (B4, B42, and B123) applied to all samples with spatial data?

The authors must explain it clearly to the readers. And if the method was applied in the second way I described above, the authors must present careful benchmark data to demonstrate sensitivity and specificity, using a series of ground-truth positive and negative samples, other than the "training set," as references.

Response: We thank the reviewer for raising this important point. To improve the clarity, we have revised the Methods and Results sections accordingly. Specifically, the sample-specific ecDNA signature was applied on a case-by-case basis, using prior knowledge on ecDNA structures derived from long-read sequencing to predict the presence of known ecDNA structures within Visium spots. For example, the B4 signature was applied exclusively to sample B4, as correctly pointed out by the reviewer.

Comment 4. In Figure 5i and also in the main text, the authors proposed that APOBEC3B-mediated mutagenesis promotes ecDNA formation: "These findings support a model in which

EMT-associated chromatin relaxation permits elevated L1 transcription, leading to increased DNA damage and APOBEC3B activation, thereby promoting genomic fragmentation and circularization events that underlie ecDNA formation.” In a previous report studying the APOBEC3B mutagenesis and ecDNA evolution (PMID: 35140399, also referred by the authors), it proposes that most of the APOBEC3B-mediated katagies mutational events likely occurred after ecDNA formation and clonal amplification, which likely serves as a mechanism to drive ecDNA evolution, rather than formation. In this manuscript, the authors identified APOBEC3B activity upregulation (potentially caused by L1 activation) as an early event, potentially driving ecDNA formation. The differences between this and previous discoveries could be cancer-type specific or due to sampling biases. Alternatively, this might be a novel mechanism specific to L1-mediated ecDNA formation. The authors do discuss their hypothesis in the discussion section, but the conclusions from the previous studies suggesting APOBEC is unlikely to be involved in ecDNA formation are not fully emphasized.
Response: Thank you for this comment. We agree that prior studies, including PMID: 35140399, primarily support a role for APOBEC3B in post ecDNA-formation mutagenesis and evolution of ecDNA. Our findings suggest that APOBEC3B activation may happen prior to ecDNA formation in cancer types with EMT-associated chromatin relaxation and L1 activity. We have revised the text of the manuscript to make this distinction clear and to emphasize that these mechanisms are not mutually exclusive and may be cancer type- or context-dependent.

Reviewer #6 (Remarks to the Author)

I co-reviewed this manuscript with one of the reviewers who provided the listed reports. This is part of the Nature Communications initiative to facilitate training in peer review and to provide appropriate recognition for Early Career Researchers who co-review manuscripts.(Remarks on code availability)
Response: Thank you for co-reviewing the manuscript.
